# Organic Functional Groups and Their Substitution Sites in Natural Flavonoids: A Review on Their Contributions to Antioxidant, Anti‐Inflammatory, and Analgesic Capabilities

**DOI:** 10.1002/fsn3.70191

**Published:** 2025-04-30

**Authors:** Jingxian An, Zhipeng Zhang, Anwen Jin, Muqiu Tan, Shilong Jiang, Yilin Li

**Affiliations:** ^1^ Chemical and Materials Engineering The University of Auckland Auckland New Zealand; ^2^ Jiangxi Copper Technology Institute Co., Ltd. Nanchang China; ^3^ Heilongjiang Feihe Dairy Co., Ltd Beijing China

**Keywords:** analgesic ability, anti‐inflammatory, antioxidant, natural flavonoids, organic functional groups, substitution sites

## Abstract

Natural flavonoids are regularly consumed orally and are known to possess antioxidant, anti‐inflammatory, and analgesic properties. Yet, there is limited understanding of the role of organic functional groups in imparting these properties. This review paper suggests that several organic functional groups, including the hydroxyl, methoxy, glycosyl, prenylated, and flavonoid groups, play crucial roles in determining the antioxidant, anti‐inflammatory, and analgesic abilities of flavonoids. Of particular significance is the contribution of the prenylated group, which notably enhances the anti‐inflammatory and analgesic abilities of flavonoids. Among isoflavones, the prenylated groups are primarily situated at C6. Despite their importance, prenylated flavonoids have not received sufficient attention from researchers. Another crucial class of organic functional groups is glycosyl groups, with C3 being a key substitution site among anthocyanins because monosaccharides are commonly found at this position. Conversely, the presence of trisaccharides or a combination of disaccharides and monosaccharides within flavonoids appears to impede their anti‐inflammatory and analgesic properties. Additionally, the majority of biflavonoids, excluding polymerized flavanols, demonstrate either anti‐inflammatory or analgesic abilities. C8 holds paramount importance among flavanols as the main substitution site for flavonoid substitution. Examination of the significance of substitution sites in flavanones, flavonols, flavones, and chalcones, which possess antioxidant, anti‐inflammatory, and analgesic abilities, revealed the importance of total substitution with diverse organic functional groups. Insights from this review can provide the guiding light to the discovery of flavonoids with antioxidant, anti‐inflammatory, and analgesic abilities in the future.

## Graphical abstract

Analgesic flavonoids exhibit both antioxidant and anti‐inflammatory properties, with prenylated groups potentially enhancing their effects. In contrast, biflavonoids generally demonstrate either anti‐inflammatory or analgesic capabilities. Furthermore, key substitution sites in these compounds can be fully replaced by various organic functional groups, leading to diverse biological activities.

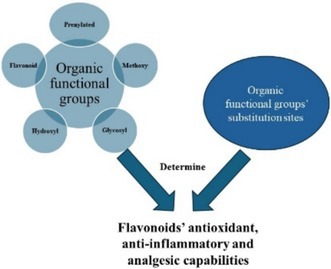

**Abbreviations**
COX: cyclooxygenases
DPPH: 2,2‐Diphenyl‐1‐picrylhydrazyl
IL‐1β: interleukin‐1β
IL‐6: interleukin 6
IL‐8: interleukin 8
iNOS: inducible nitric oxide synthase
LOX: lipoxygenases
LPS: lipopolysaccharide
NF‐κβ: nuclear factor‐κβ
TNF‐α: tumor necrosis factor‐α

## 1 Introduction

Pain is defined as “an unpleasant sensory and emotional experience associated with actual or potential tissue damage” (Lee and Neumeister 2020). Typically, pain and inflammatory complications have posed a significant burden on social and healthcare systems, affecting humans for centuries. Globally, 30% of adults experience pain and inflammatory diseases, with 20% receiving diagnoses of chronic ailments each year (Javed et al. 2020). This escalating incidence and prevalence of pain and associated disorders add to the growing challenges we encounter.

Based on an extensive literature review, a strong correlation has been established between inflammation, pain, and oxidative stress (Adedapo et al. 2015). Inflammation signifies an intricate and crucial defense mechanism exhibited by the host, often triggered by microbial infections and frequently accompanied by pain. Notably, pro‐inflammatory cytokines such as TNF‐α, IL‐6, and IL‐1β can induce sensitization or activation of nociceptors, leading to the perception of pain (Ferraz et al. 2020; Kumar et al. 2015). Consequently, any research pertaining to inflammation must also encompass an analysis of its analgesic effects (Adedapo et al. 2015). Moreover, tissue injury during inflammation gives rise to free radicals, including oxygen‐derived radicals or reactive oxygen species (ROS) and nitrogen‐derived radicals or reactive nitrogen species (RNS), which exert detrimental effects on cell function (Adedapo et al. 2015).

Traditionally, pain and inflammation have been regarded as unpleasant and nonspecific symptoms of numerous diseases. Classical analgesics of natural origin, such as opiates and nonsteroidal anti‐inflammatory drugs (NSAIDs), are commonly utilized. However, they often come with side effects such as gastric lesions, drug tolerance, and drug dependence. Therefore, exploring alternative natural sources to NSAIDs and opiates is imperative (Faujdar et al. 2016). Nowadays, flavonoids have emerged as potential alternatives for their anti‐inflammatory and analgesic properties, and some authors have attempted to summarize the significance of organic functional groups in determining flavonoids' antioxidant and anti‐inflammatory abilities (Hernández‐Rodríguez et al. 2019; Wang et al. 2018). However, to date, few authors have addressed the subclasses of flavonoids with these properties and have rarely focused on the importance of substitution sites within subclasses of flavonoids. This review aims to systematically report and comprehensively study subclass flavonoids with antioxidant, anti‐inflammatory, and analgesic properties (the summarized flavonoids are listed in the Appendix S1 from scientific papers). Furthermore, the review summarizes the importance of organic functional groups and their substitution sites of flavonoids.

Most importantly, the significance of organic functional groups and their substitution sites is further investigated using machine learning algorithms. Machine learning algorithms are computational processes that utilize input data to achieve desired outcomes without being explicitly programmed for specific tasks (El Naqa and Murphy 2015). Among these algorithms, feature selection methods are particularly valuable as they identify relevant features in a dataset while eliminating redundant and irrelevant ones (Jie et al. 2018). In this study, feature selection methods are employed to determine the most critical organic substitution sites influencing the antioxidant, anti‐inflammatory, and analgesic capabilities of flavonoids. Additionally, the types of organic functional groups present at these key substitution sites are examined to elucidate their relationship with bioactivity.

## 2 Flavonoids and Their Properties

Flavonoids, a substantial group of phenolic compounds widely distributed across the plant kingdom, are illustrated with C6‐C3‐C6 rings. As important secondary metabolites of natural origin, these compounds constitute a significant part of the human diet. In Western countries, it is estimated that as much as 200–500 mg of flavonoids are ingested daily (Mascaraque et al. 2015). Flavonoids have been gaining increasing attention in the food industry due to their distinct benefits in enhancing the quality and nutritional value of foods. Moreover, flavonoids have been documented to possess various pharmacological activities, such as antioxidant, anti‐inflammatory, and analgesic abilities (Chen et al. 2019; Jiménez‐Aguilar and Grusak 2017).

Research on flavonoids has primarily focused on exploring their potential antioxidant capacities. Flavonoids exhibit antioxidant behavior through various mechanisms, including the chelation of transition metals involved in radical formation, direct scavenging of ROS, inhibition of enzymes such as xanthine oxidase (XO) responsible for generating superoxide anions, and prevention of peroxidation processes by reducing alkyl and peroxyl radicals (Silva et al. 2021). Therefore, flavonoids with antioxidant action play an important role in food or the human body by neutralizing oxidation processes and preventing chronic diseases related to oxidative stress.

It is important to note that flavonoid's antioxidant properties can also contribute to their anti‐inflammatory effects. For instance, flavonoids demonstrate both antioxidant and pro‐oxidant effects, inhibit the expression of inflammation‐related genes, interact with signaling pathways, and bind to pro‐inflammatory proteins, including specific enzyme inhibitors (Ballard and Junior 2019). Notably, flavonoids can inhibit key pro‐inflammatory enzymes, such as cyclooxygenases (COX), lipoxygenases (LOX), and NO synthase (NOS), which are known to mediate inflammatory responses, thereby providing therapeutic benefits (Ballard and Junior 2019). Among these, COXs play a central role in the biosynthesis of prostaglandins, which contribute to both inflammation and pain. Consequently, the inhibition of COXs by flavonoids may contribute to their dual anti‐inflammatory and analgesic effects (Panche et al. 2016; Pinho‐Ribeiro et al. 2016). As an illustration, naringenin has been demonstrated to inhibit pain‐like behavior induced by various inflammatory stimuli such as phenyl‐p‐benzoquinone, acetic acid, formalin, carrageenan, superoxide anion, and lipopolysaccharide (LPS) (Komakech et al. 2019). LPS serves as a potent monocyte activation signal that significantly affects the production of inflammatory cytokines, including IL‐1β, TNF‐α, IL‐6, IL‐8, prostaglandin D_2_, prostaglandin E_2_, and thromboxane B_2_ (Mayer et al. 2011; Tajima et al. 2008). Additionally, catechin has shown potential anti‐inflammatory and analgesic activities attributed to its modulation of oxidative stress and inflammation through cell signaling pathways such as NF‐κβ and mitogen‐activated protein kinases (Komakech et al. 2019). These observations imply a strong interconnection among the antioxidant, anti‐inflammatory, and analgesic capabilities exhibited by flavonoids.

**FIGURE 1:**
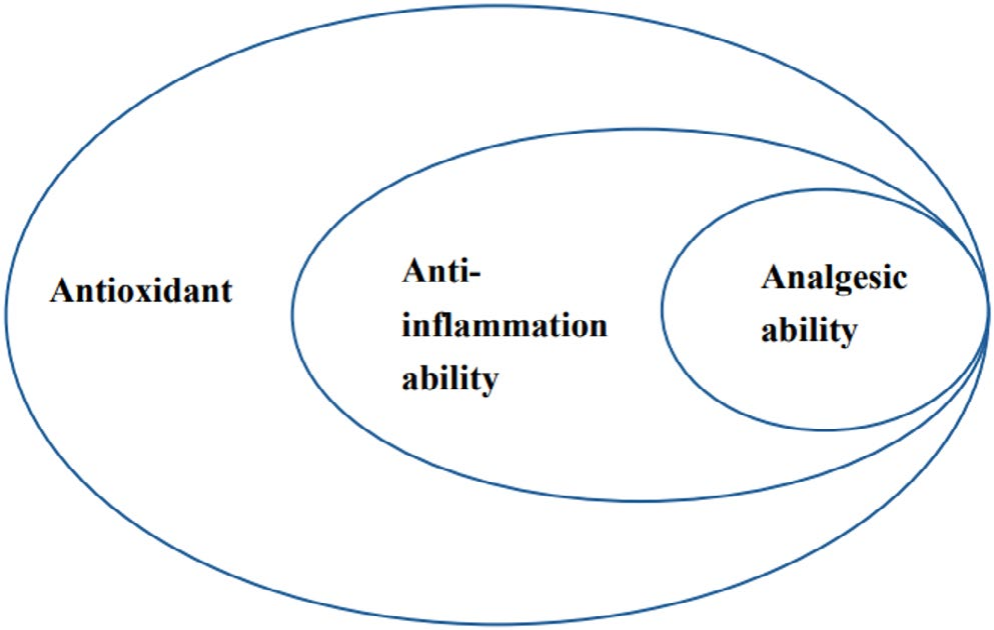
The relationship between antioxidant, anti‐inflammatory, and analgesic abilities of flavonoids.

The summary of flavonoids featuring antioxidant, anti‐inflammatory, and analgesic abilities can be found in Tables S1–S7 of the Appendix S1 with related references. It is noteworthy that certain flavonoids may unveil additional therapeutic potential, including anti‐inflammatory and analgesic abilities, in future research. Based on the data presented in Tables S1–S7, a hierarchical relationship among these properties can be inferred: analgesic abilities→anti‐inflammatory abilities→antioxidant abilities. In other words, flavonoids that typically exhibit analgesic capabilities, alongside essential antioxidants and anti‐inflammatory activities. While flavonoids typically demonstrate anti‐inflammatory capabilities, they also consistently show antioxidant effects. However, it is crucial to recognize that although many flavonoids exhibit significant antioxidant activity, this does not necessarily translate into anti‐inflammatory or analgesic effects. Notably, Figure 1 underscores the intrinsic nature of flavonoids to possess both anti‐inflammatory and antioxidant attributes.

## 3 Basic Skeleton of Flavonoids and Their Antioxidant, Anti‐Inflammatory, and Analgesic Abilities

**FIGURE 2:**
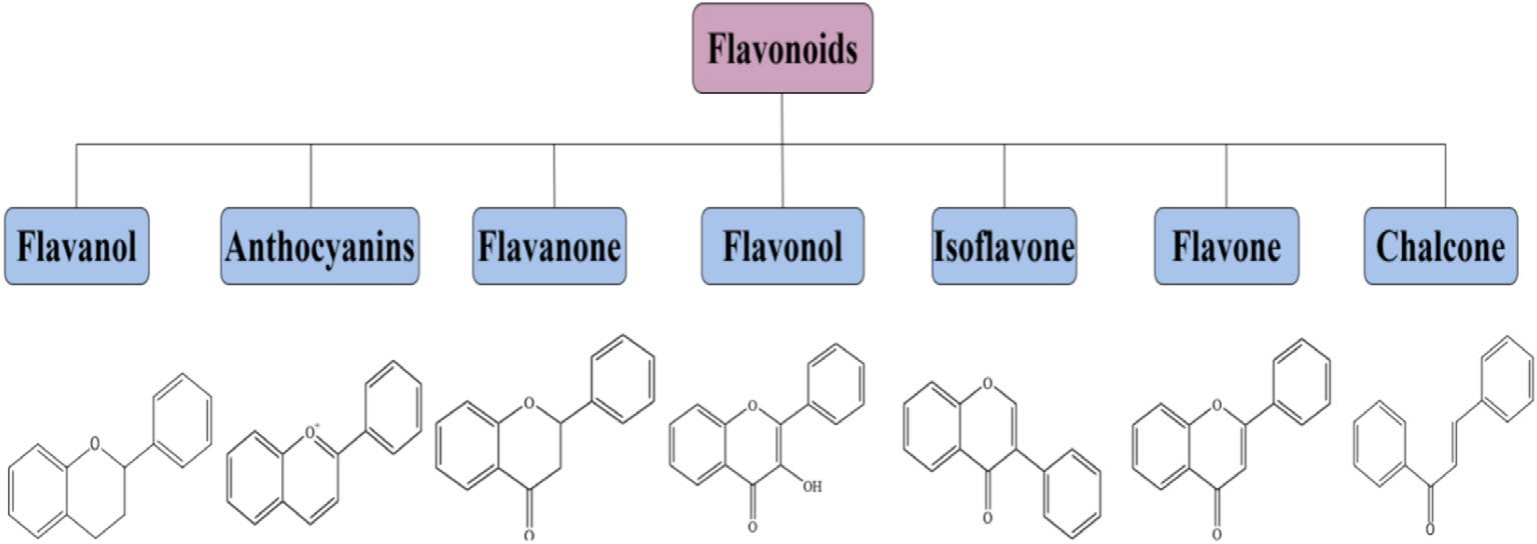
Chemical structures of flavonoids with various carbon skeletons.

**FIGURE 3:**
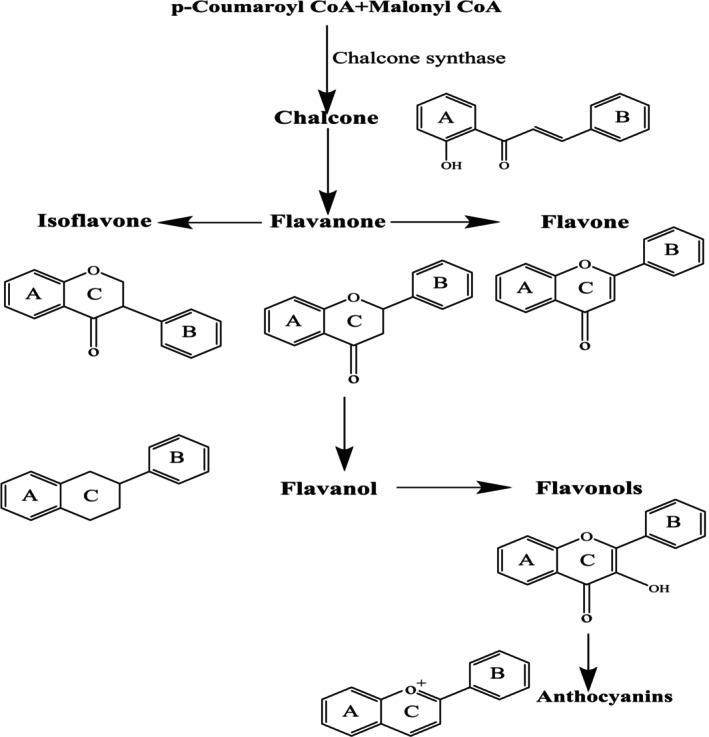
Flavonoids with biosynthesis.

Based on their structures, flavonoids are typically categorized into seven subclasses: flavanols, flavones, isoflavones, anthocyanidins, flavanones, flavanols, and chalcones. This classification methodology is rooted in the oxidation level of the central heterocycle, progressing from low to high as depicted in Figure 2 (Nagula and Wairkar 2019). Moreover, Figure 3 illustrates the relationship between flavonoids belonging to different subclasses in biosynthesis (Dias et al. 2021). This section delves into the discussion of flavonoids possessing antioxidant, anti‐inflammatory, and analgesic properties across various subclasses.

### 3.1 Flavanols

Flavanols are present in significant quantities in widely consumed foods such as tea, grapes, wine, cocoa, and chocolate. However, the chemical structure of flavanols varies among these foods. For instance, flavanols in tea are predominantly found as galloylated compounds and their oligomers, while in grapes, they exist as monomers or in the form of tannins (Perez‐Vizcaino and Fraga 2018). In cocoa, flavanols are present as monomers and oligomers of epicatechin, also known as proanthocyanidins (tannins) (Perez‐Vizcaino and Fraga 2018). Catechin, a type of flavanol, is known for its potent anti‐inflammatory and analgesic activities, which are attributed to its ability to modulate inflammation and oxidative stress through cell signaling pathways, including NF‐κβ and mitogen‐activated protein kinases (Komakech et al. 2019). The chemical structures of flavanols exhibiting antioxidant, anti‐inflammatory, and analgesic abilities are illustrated in Figure S1.

Figure S1 indicates that the hydroxyl group is the most prevalent organic functional group in flavanols. Generally, flavanols exhibit a total number of hydroxyl groups above five, with each aromatic ring typically containing at least two hydroxyl groups, which might potentially undergo polymerization reactions in the future. Tannins, which are polymerized flavanols, not only contribute to the bitter taste but also possess anti‐inflammatory properties (Komakech et al. 2019). However, Figure S1 implies that individual polymerized flavanols (condensed tannins) possess only antioxidant abilities. The anti‐inflammatory and analgesic abilities of tannins may arise from certain hydrolyzed tannins, such as gallic acid, which has been demonstrated to possess anti‐inflammatory and analgesic abilities (Bai et al. 2021; Kaur and Muthuraman 2019).

### 3.2 Anthocyanins (And Anthocyanidins)

Anthocyanins are predominantly found in berry‐type fruits and red wines, contributing to the blue, red, and purple colors of fruits and vegetables, while also possessing antioxidative and anti‐inflammatory functions (Ballard and Junior 2019; Nagula and Wairkar 2019; Zhang et al. 2016). These compounds exhibit antioxidant activity by acting as either exogenous or intrinsic antioxidants, capable of scavenging ROS or nitrogen species, thereby mitigating oxidative stress and preventing cellular damage (Nagula and Wairkar 2019; Zhang et al. 2016). Additionally, anthocyanins extracted from Korean black soybeans have been found to reduce neuroinflammation by inhibiting specific inflammatory markers and NF‐κβ (Spagnuolo et al. 2018). These properties are also exhibited by anthocyanidins and are not exclusive to anthocyanins (anthocyanins are in the form of glycosides while anthocyanidins are known as the aglycone). Notably, anthocyanidins such as cyanidin and delphinidin have been identified for their abilities to inhibit oxidative stress and NF‐κβ activation (Daveri et al. 2018). The antioxidant, anti‐inflammatory, and analgesic properties of anthocyanins and anthocyanidins are showcased in Figure S2.

Figure S2 indicates that anthocyanidins with hydroxyl groups may generate analgesic effects. Typically, these compounds exist as glycosylated forms, with glycosylation occurring at the C3 substitution site (Spagnuolo et al. 2018). Additionally, those with monosaccharides might possess anti‐inflammatory properties, as observed with Figure S2 and Table S2. These findings are supported by relevant references (Huang et al. 2014; Vezza et al. 2016; Zhao et al. 2019). However, when anthocyanins have two monosaccharides, they are more likely to lose their anti‐inflammatory effects and exhibit only antioxidant properties. This shift occurs because anthocyanidins inherently possess positive charges, rendering them more hydrophilic compared to other flavonoid aglycones (Dudek et al. 2022). When two monosaccharides are connected to anthocyanidins, they become highly hydrophilic and, as a result, cannot be readily absorbed orally by the human body.

### 3.3 Flavanones

Flavanones are predominantly found in citrus fruits, such as oranges and lemons, contributing to their bitter tastes and offering various health benefits (Pinho‐Ribeiro et al. 2015). Common flavanones, such as naringin and naringenin, exhibit notable antioxidant properties and potential anti‐inflammatory effects, alleviating associated pain (Dong et al. 2019; Ge et al. 2019; Hernández‐Rodríguez et al. 2019; Khan et al. 2020; Komakech et al. 2019; Silva et al. 2021). Specifically, naringenin, a bitter and colorless monomeric flavanone, belongs to a class of flavonoids with potent anti‐inflammatory activity due to its ability to reduce pro‐inflammatory cytokines (Komakech et al. 2019; Pinho‐Ribeiro et al. 2015). Furthermore, naringin is capable of modulating the activity of antioxidant enzymes such as catalase (CAT), superoxide dismutase (SOD), glutathione peroxidase (GPx), and paraoxonase (PON), and also exhibits significant antioxidant properties through its free radical scavenging capacity (Eom et al. 2021; Liu et al. 2017; Mamdouh and Monira 2004).

A comprehensive summary of flavanones showcasing antioxidant, anti‐inflammatory, and analgesic abilities is presented in Figure S3. According to the figure, prenylated groups at the C6 position are commonly found in flavanones. Among these, flavanones characterized by C6 prenylated groups have attracted significant interest due to their strong bioactive potential, such as inhibiting bacterial growth (Chen et al. 2012; Osorio et al. 2021). Additionally, prenylated flavanones have been reported to inhibit nitric oxide (NO) production by interfering with inducible nitric oxide synthase (iNOS) expression (Chen et al. 2012).

### 3.4 Flavonols

Flavonols are primarily found in the epidermal cells of plant tissues and serve as a protective barrier against harmful solar wavelengths, particularly UV rays, to safeguard DNA integrity (Nagula and Wairkar 2019). Flavonols can be broadly classified into several types such as kaempferol, quercetin, myricetin, and galangin, which are abundant in certain vegetables and fruits like broccoli, onions, asparagus, and apples. Quercetin, a prominent member of the flavonol subclass, represents a significant proportion (60%–75%) of total dietary intake of flavonoids (Spagnuolo et al. 2018). It exhibits beneficial effects by inhibiting ROS‐mediated hepatocarcinogenesis through the upregulation of enzymatic antioxidants such as SOD, GPx, CAT, as well as nonenzymatic antioxidants like reduced glutathione (GSH) and total glutathione (Spagnuolo et al. 2018).

The summarized flavonols with antioxidant, anti‐inflammatory, and analgesic abilities are presented in Figure S4. As shown in Figure S4, the presence of a C3‐hydroxyl group (OH) in flavonols is associated with potential anti‐inflammatory effects. For instance, quercetin, distinguished by a C3‐OH group, has been reported to enhance the inhibition of LPS‐stimulated cytokines (Zaragozá et al. 2020). The significance of the free hydroxyl group at the C3 substitution site of quercetin is noteworthy, contributing to the inhibition of COX‐2. In contrast, quercetin‐3‐glucuronide, with glucuronide at the same substitution site, exhibits reduced effectiveness (Silva et al. 2021). Additionally, modifications to the hydroxyl groups between the rings provide insights into specific molecular changes influencing the activity of inflammatory mediators (Chen et al. 2019). Hydroxyl groups at substitution sites C3, C5, and C6, for instance, are crucial for interactions with phospholipase A_2_ (PLA_2_), a significant target in inflammatory mediation. Moreover, the role of monosaccharides in contributing to flavonols' analgesic abilities is noteworthy. Quercitrin, identified as quercetin‐3‐rhamnoside, has demonstrated a significant inhibitory effect on acetic acid‐induced visceral pain in mice (Zhao et al. 2018).

### 3.5 Isoflavones

The primary sources of isoflavones are soy and soy‐derived products, with genistein and daidzein being the major isoflavones found in these sources (Perez‐Vizcaino and Fraga 2018). Isoflavones exhibit potent antioxidant properties by scavenging free radicals, protecting against lipid peroxidation, and reducing oxidative stress caused by UV damage (Nagula and Wairkar 2019). For anti‐inflammatory activity, Yu et al. (2016) demonstrated that genistein effectively inhibits LPS‐induced inflammation and reduces the production of IL‐1β, IL‐6, and PGE2. Furthermore, genistein, a specific isoflavone, has been found to possess local anesthetic properties and may offer relief from trigeminal nociceptive pain without causing side effects (Yamaguchi et al. 2021). Similar support is provided by Xu et al. (2019), who demonstrated that genistein and daidzein exhibit the ability to block voltage‐gated sodium channels, thereby attenuating chemically and heat‐induced acute pain. Figure S5 provides a concise summary of the antioxidant, anti‐inflammatory, and analgesic properties of isoflavones. By referring to Figure S5, the majority of identified isoflavones are likely to exhibit anti‐inflammatory and analgesic properties.

According to Figure S5, prenylated groups in isoflavones are commonly found at the C6 and C8 positions. This observation aligns with the findings of van Dinteren et al. (2021), who reported that prenylated groups are frequently present in isoflavones. Jheng et al. (2023) further demonstrated that the C8‐prenylated group exhibits stronger anti‐inflammatory properties compared to other substitutions. Meanwhile, C6‐prenylated groups have been shown to possess strong bioactive abilities, such as antibacterial abilities (Kalli et al. 2020).

### 3.6 Flavones

Flavones, known for their distinct yellow pigmentation, are predominantly present in citrus fruits (He et al. 2016). Luteolin, the most abundant flavone in plants, is prominently found in celery, parsley, green pepper, chamomile tea, and perilla leaf. Scientific studies have demonstrated its potent anti‐inflammatory effects, characterized by the suppression of inducible nitric oxide synthase (iNOS) and cyclooxygenase‐2 (COX‐2) expression. Additionally, luteolin downregulates pro‐inflammatory cytokines and mitigates the production of nitric oxide (NO) and prostaglandin E_2_ in BV2 microglia cells induced by LPS (Spagnuolo et al. 2018). Figure S3 offers a comprehensive overview of flavones showcasing antioxidant, anti‐inflammatory, and analgesic properties.

Figure S6 suggests that the anti‐inflammatory activity of citrus flavonoids is closely related to their individual flavonoid structures. For instance, flavones with 4′‐OH groups, or with 3′,4′‐hydroxyl substituents on the B ring, are selective lipoxygenase inhibitors, while flavones with five or more methoxy substituents exhibit greater inhibitory activity for phosphodiesterase (Chen et al. 2017). Another example, apigenin, a type of flavone containing a methoxy group, is known for its ability to scavenge free radicals, which are reactive molecules that can damage cells and contribute to inflammation. By acting as a free radical scavenger, apigenin helps reduce oxidative stress and protect pancreatic cells from damage. It also plays a role in regulating the antioxidant defense system in these cells, further enhancing their ability to combat inflammation (Komakech et al. 2019). In the context of neuroinflammation, apigenin has been shown to inhibit the production of pro‐inflammatory cytokines such as IL‐1β and TNF‐α. These cytokines are key mediators of inflammation and can contribute to the progression of neurodegenerative diseases. By inhibiting their production, apigenin helps to reduce inflammation and potentially protects against neuroinflammatory conditions (Komakech et al. 2019).

### 3.7 Chalcones

Chalcones are a class of compounds with an open C ring structure, and they serve as precursors for the synthesis of flavonoids and isoflavones. These compounds are widely distributed in tomatoes, pears, strawberries, bearberries, and certain wheat products (Panche et al. 2016). Figure S7 summarizes the antioxidant, anti‐inflammatory, and analgesic abilities of chalcones.

As shown in Figure S7, the majority of chalcones possess anti‐inflammatory or analgesic abilities. For example, 2′,4′‐dihydroxychalcone, characterized by hydroxyl groups, has been investigated for its anti‐inflammatory properties. Research indicates its ability to impede the production of NO, a pro‐inflammatory molecule crucial in immune response and inflammation. The anti‐inflammatory effects of 2′,4′‐dihydroxychalcone are attributed to its inhibitory action on NO production (Zhang et al. 2020). Moreover, hesperidin methyl chalcone (HMC), classified as a chalcone and featuring a disaccharide, has been shown to inhibit the production of cytokines (IL‐1β, IL‐6, IL‐10, and TNF‐α), mitigating oxidative stress, and restraining NF‐κβ activation in carrageenan‐induced inflammation. Additionally, HMC exhibits the capability to alleviate inflammatory pain by modulating the activity of the TRPV1 receptor (Pinho‐Ribeiro et al. 2015).

## 4 Organic Functional Groups in Flavonoids

**FIGURE 4:**
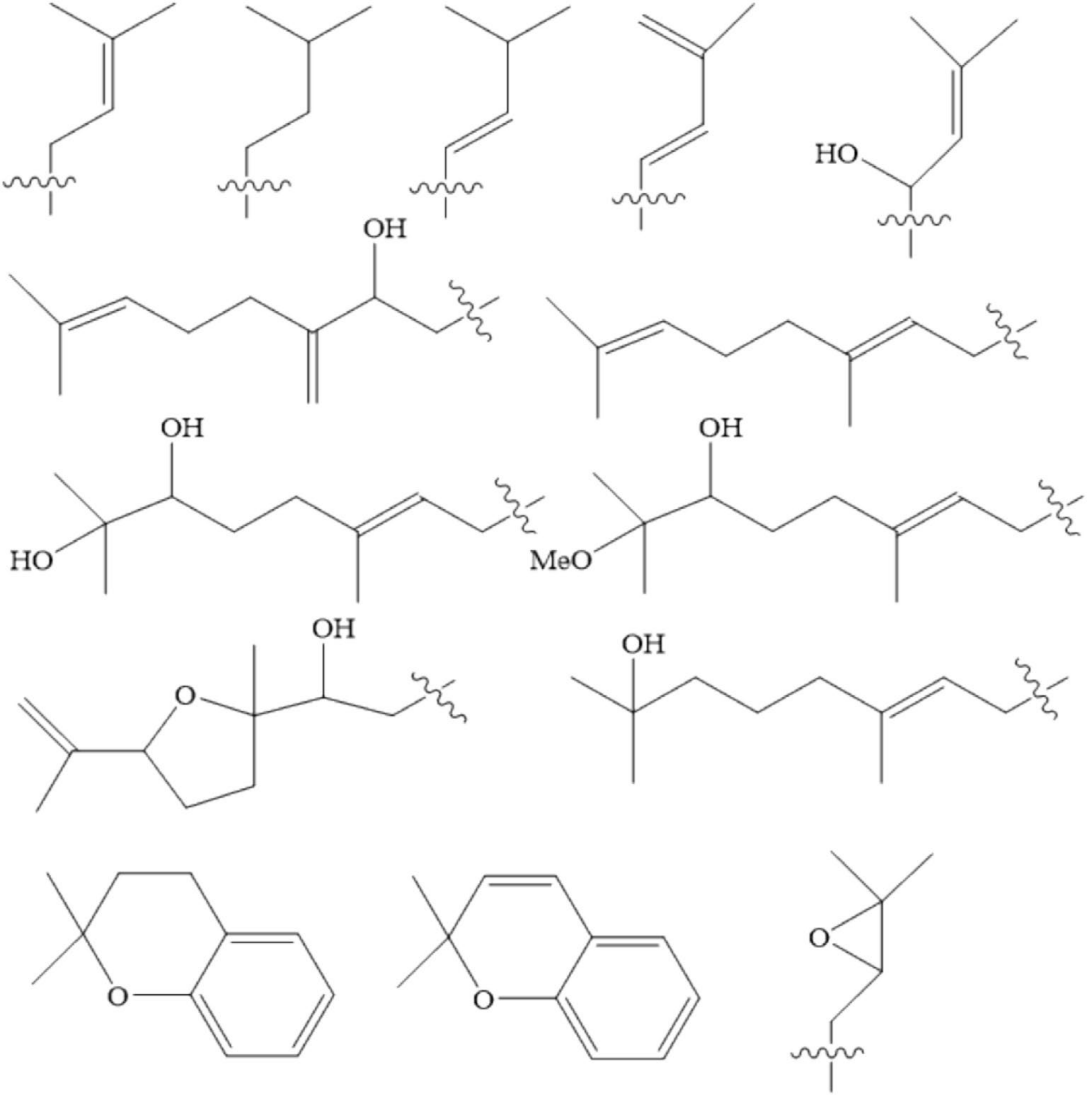
Prenylation patterns occurred on flavonoids (Yang et al. 2015).

Figures S1–S7 collectively indicate that hydroxyl, methoxy, prenylated, glycosyl, and flavonoid groups contribute to the antioxidants, anti‐inflammatory, and analgesic abilities of flavonoids. Table 1 summarizes various organic functional groups found in different subclasses of flavonoids with these beneficial properties. Among different subclasses of flavonoids, the hydroxyl group emerges as the predominant organic functional group, particularly evident in flavanols (excluding polymerized forms). Prenylated groups are present in five of the seven flavonoid subclasses, with isoflavones exhibiting the highest proportion of prenylated compounds. The significance of organic functional groups in determining the antioxidant, anti‐inflammatory, and analgesic properties of flavonoids is discussed in the following sections (Figure 4).

**TABLE 1:** Various subclasses of flavonoids and their functional groups.

Flavonoid subclass	Organic functional groups					Organic functional groups on medicinal effects
	Hydroxyl	Methoxy	Prenylated	Glycosyl	Flavonoid	
Flavanol	√				√	Prenylated and flavonoids groups (except polymerized flavanols) enhance anti‐inflammatory and analgesic abilities. Excessive glycosyl groups diminish anti‐inflammatory and analgesic abilities.
Anthocyanin and anthocyanidin	√	√		√		
Flavanone	√	√	√	√	√	
Flavonol	√	√	√	√	√	
Isoflavone	√	√	√		√	
Flavone	√	√	√	√	√	
Chalcone	√	√	√	√		

*Note:* √ indicates presence of organic functional groups in the flavonoid subclass.

### 4.1 Hydroxyl Group

Flavonoids are characterized by the presence of two or more aromatic rings, typically containing at least one aromatic hydroxyl group attached to a heterocyclic pyran. These compounds exhibit an aromatic structure and a highly conjugated system, featuring multiple hydroxyl groups. This configuration enables them to serve as efficient donors of electrons or hydrogen atoms, neutralizing free radicals and ROS (Kim et al. 2020). Several researchers have observed a significant correlation between the number of phenolic hydroxyl groups and antioxidant activity of flavonoid aglycones (Parcheta et al. 2021). The observed correlation can be attributed to several factors: (1) an increase in the number of phenolic hydroxyl groups leads to more H^+^ combined with free radicals; (2) the phenolic hydroxyl has strong electron‐donating effect, which facilitates the reaction with free radicals (3) a higher number of phenolic hydroxyl groups promotes more hydrogen bonding, thereby significantly enhancing antioxidant activity (Zuo et al. 2018). Nevertheless, the correlation between the number of hydroxyl groups and antioxidant activity is not proportional when sugar moieties are present in flavonoids. An illustrative case is seen in quercetin 3‐glycosides, where the antioxidant activity was found to be stronger than that of quercetin alone (Zhang et al. 2016). In terms of antioxidant activities, the hierarchy from high to low is observed as follows: cyanidin 3‐glycosides > cyanidin 3,5‐diglycosides > cyanidin 3‐(6″‐malonyl)‐glucopyranoside > delphindin 3,5‐glycosides (Zhang et al. 2016). Regarding the anti‐inflammatory effect, polyhydroxylated flavonoids have demonstrated a greater ease in inhibiting COX and LOX (Zuo et al. 2018). Quercetin and luteolin, featuring C3′ and C4′ hydroxylation groups, show the highest inhibition of TNF‐α, surpassing genistein, kaempferol, apigenin, diosmetin, and hesperetin, which possess only one hydroxyl group on the B ring (Zeinali et al. 2017).

### 4.2 Methoxy Group

Replacing hydroxyl groups with methoxy groups has been observed to reduce cytotoxicity. However, it is crucial to highlight that such substitution may potentially diminish the antioxidant activity of the compounds. Conversely, the methylation process significantly enhances the anti‐inflammatory properties of flavonoids, possibly facilitated by the ionization of hydroxyl groups and a more pronounced inhibition of the NF‐κβ signaling pathway (Chen et al. 2018). O‐methylation of chrysin, for instance, may result in metabolically more stable flavonoids with increased bioavailability and higher tissue distribution compared to their unmethylated forms (Chen et al. 2018). Importantly, the methoxy group in the C3 substitution site reduces cytotoxicity (Chen et al. 2019).

### 4.3 Prenylated Group

Prenylated flavonoids have been overlooked by researchers due to their low abundance and limited presence in plant species (Peng et al. 2024; Yang et al. 2015). These flavonoids are predominantly present in the Moraceae family, with sporadic occurrences in other plant families such as Fabaceae, Guttiferae, Canabaceae, Rutaceae, Umbelliferae, Euphorbiaceae Asteraceae, and Thymelaeaceae. Prenylated flavonoids contain a lipophilic prenylated sidechain, incorporating alky‐substituent groups, such as prenyl, geranyl, and farnesyl. The variations of these alkyl groups depend on their hydroxylation, cyclization, and oxidation (Shi et al. 2021). The addition of a prenylated group to specific carbon substitution sites on the molecular structure (C‐prenylation) is frequently observed at substitution sites C‐6/C‐8 on ring A and C‐3′ and C‐5′ on ring B, typically substitution sites ortho to a phenolic hydroxyl group. In natural prenylated flavonoids, C‐prenylation on ring C is relatively uncommon (Yang et al. 2015).

At present, the prenylated group has been detected in various flavonoid subclasses, including flavanols, flavanones, isoflavones, isoflavone, chalcone, and flavones. Prenylated flavonoids exhibit enhanced lipophilicity in comparison to their non‐prenylated counterparts (Chen et al. 2014). This heightened lipophilicity enhances their affinity for cell membrane, significantly amplifying their biological activities and pharmacological effects (Shi et al. 2021). Specifically, prenylated flavanones have been reported to exhibit superior antioxidant activity when compared to their non‐prenylated counterparts (Chen et al. 2014).

Most prenylated flavonoids exhibit moderate anti‐inflammatory activity in LPS‐stimulated RAW 264.7 cells by down‐regulating COX‐2 induction at concentrations of 10–25 μM (Shi et al. 2021). Due to their ability to inhibit COX and LOX, prenylated flavonoids have garnered interest as potential anti‐inflammatory agents. Exploration of prenylated flavonones is currently limited, yet studies indicate that the C‐6 prenylation of the flavanone skeleton plays a crucial role in their in vitro inhibitory potential against both COX‐1 and COX‐2 (Hanáková et al. 2017).

### 4.4 Glycosyl Group (Sugar Moiety)

Sugars are linked to flavonoid skeletons through two primary mechanisms, resulting in either C or O‐glycosides. In C‐glycosides, sugar moieties are directly attached to the nuclei through a stable C‐C bond that is resistant to hydrolysis. On the other hand, O‐glycosides involve sugar moieties connected to aglycones through an acid‐hydrolysable O‐C bond. Generally, C‐glycosides are less prevalent than O‐glycosides and are found exclusively in specific plant groups, often with single or double substitutions occurring at the C‐6 and/or C‐8 substitution sites (Seo et al. 2019). Common glycosyl groups found in flavonoids include monosaccharides such as glucoside, rhamnoside, glucuronide, and arabinoside. The presence of different sugar residues on aglycones has been observed to influence bioavailability (Wang et al. 2018). For example, the absorption rate of quercetin glucoside was more than 10 times faster than that of quercetin rutinoside (Hollman 2004). This resulted in an approximately 20 times higher quercetin concentration in the plasma compared to the intake of quercetin rutinoside (Wang et al. 2018).

Glycosylation of flavonoids has been reported to increase water solubility, reduce toxicity, and in some cases, modify the original pharmacological and biological activities (Seo et al. 2019). An illustrative example is observed in quercetin 3‐O‐rhamnoside, which exhibits a pronounced inhibitory effect on the formation of advanced glycation end products (AGEs) formation. AGEs are well‐known for their close association with inflammatory processes (Seo et al. 2019). Meanwhile, flavonoid O‐glucosides have been found to exhibit comparable binding affinities to COX‐2 when compared to flavonoid C‐glycosides (Chen et al. 2019). Moreover, quercitrin, hyperoside, tiliroside, astragalin, and kaempferol 3‐O‐rutinoside display varying degrees of anti‐inflammatory activity, with tiliroside demonstrating stronger efficacy than the others. The anti‐inflammatory effects of tiliroside are suggested to be mediated through the reduction of NO and the suppression of pro‐inflammatory cytokines (TNF‐α, iNOS, and IL‐6) production (Zhang et al. 2015).

**FIGURE 5:**
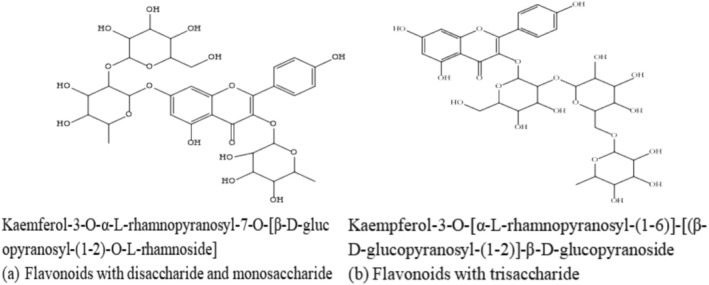
Chemical structures of flavonoids with three sugar moieties.

It is crucial to note that flavonoids with disaccharide and monosaccharide, or trisaccharide, as shown in Figure 5 (as of now, no flavonoids with three monosaccharides have been identified), impede their anti‐inflammatory activity, rendering them incapable of exerting pain inhibition. The reduced anti‐inflammatory efficacy observed in flavonoids featuring glycosides of low lipophilicity can be attributed to their reduced hydrophobicity, leading to decreased membrane permeability (Wang et al. 2018).

### 4.5 Flavonoid Group

Biflavonoids, characterized by their unique dimeric structure formed through various chemical combinations, such as flavanone‐flavone, flavone‐flavone, and flavone‐flavonol, exhibit two primary bond connections: C‐C bond or C‐O‐C bond (Kim et al. 2008). Biflavonoids demonstrate diverse biological properties, including anticancer, antimicrobial, antiviral, anti‐inflammatory, and analgesic abilities (Kim et al. 2008). By referring to Figure S4 and Figures S6–S7, it can be seen that the majority of biflavonoids (except polymerized flavanols) display anti‐inflammatory and analgesic capabilities. These compounds showcase their anti‐inflammatory activity through the inhibition of phospholipase A_2_ or the regulation of pro‐inflammatory gene expression, both in vitro and in vivo (Šamec et al. 2022).

## 5 Unraveling the Significance of Substitution Site in Flavonoids

Compared to other natural chemical products, flavonoids exhibit relatively similar chemical structures. The crucial substitution sites of flavonoids (C2–C8 and C'2–C'5), which influence their antioxidant, anti‐inflammatory, and analgesic capabilities, can be identified using feature selection methods in machine learning (Please check Methodology Section in Appendix S1 for details). These techniques include extra tree classifiers, gradient boost classifiers, random forest, and extreme gradient boost. Implementation of these methods is carried out using in‐house developed Python codes. The input dataset comprises organic functional groups, including hydroxyl, methoxy, glycosyl (monosaccharide, disaccharide, and trisaccharide), prenylated, and flavonoid groups. The resulting output data delineate the medicinal effects of flavonoids, specifically in terms of their antioxidant, anti‐inflammatory, or analgesic abilities. The significance of substitution sites in flavonoids is visually depicted in Figures S8–S14, while Tables S8–S14 summarize the organic functional groups in various substitution sites across different flavonoids.

Building upon the insights from Figures S8–14 and Tables S8–S14, two primary factors emerge as determinants influencing the crucial substitution sites of flavonoids with antioxidant, anti‐inflammatory, and analgesic abilities. The first factor revolves around the abundance of biological organic functional groups in specific substitution sites. For instance, flavonoid substitution predominantly occurs at C8 in flavanols, monosaccharide is mainly found at C3 in anthocyanins, and prenylated groups are predominantly situated at C6 in isoflavones. Previous studies, including the work by Jing et al. (2015) have indicated that glycosylation at C3 of the anthocyanin skeleton significantly enhances oxygen radical absorbance capacity, signifying a robust antioxidant indicator. Concurrently, the presence of the prenylated group at C6 is known to produce isoflavones with potent activity. In contrast, research by Mukne et al. (2011) has demonstrated that the removal of prenylated groups or their relocation to C8 leads to a decrease in activity.

The second factor involves the substitution of sites by organic functional groups, resulting in a greater diversity of organic functional groups, as exemplified by flavanones, flavonols, flavones, and chalcones (Table 2). In the context of flavanones, C7 emerges as a crucial substitution site, with O‐glycosylation at C7 exhibiting a strong affinity for the cell membrane (Zhang et al. 2019).

**TABLE 2:** Distribution of organic functional groups in key substitution sites across various flavonoids.

Flavonoid subclass	Important substitution site	Organic functional groups
Flavanones	C7	Hydroxyl, glycosyl (including monosaccharide and disaccharide), and prenylated
Flavonols	C3	Hydroxyl, methoxy, glycosyl (including monosaccharides, disaccharides, trisaccharides), and prenylated
Flavones	C7	Hydroxyl, methoxy, glycosyl (including monosaccharides and disaccharides), and prenylated
Chalcones	C3′	Hydroxyl, glycosyl (including monosaccharides and disaccharides), and prenylated

Additionally, C7‐OH is recognized as the most reactive hydroxyl group in flavanones (Khan et al. 2010). For flavonols, the hydroxyl group at C3 is essential for their antioxidant properties, particularly in inhibiting H_2_O_2_, as elucidated in the study by Krol et al. (1994). Section 3.4 further discusses the significant roles played by C3‐OH in determining the antioxidant and anti‐inflammatory abilities of flavonols. In the case of flavones, glycosylation of C7 in luteolin has been shown to noticeably decrease its antiradical activity in the DPPH assay (Spiegel et al. 2020). In contrast, flavones with C7‐OH exhibit strong inhibition of xanthine oxidation (Zhang et al. 2022). A study by Zhou et al. (2021) corroborates that prenylated groups are predominantly attached to C3′. Upon closer inspection of Figure S8 and Table S14, it becomes evidence that C3′ has the highest number of prenylated groups.

## 6 Conclusion and Future Trends

This paper primarily focuses on the organic functional groups and their substitution sites in flavonoids rather than stereochemical structures. Catechin and epicatechin are presented as isomers, revealing that they share identical antioxidant, anti‐inflammatory, and analgesic abilities. Isomelacacidin and melacacidin are also isomers. Isomers such as astilibin, neoastilibin, neoisoastilibin, and isoastilibin also exhibit identical antioxidant and anti‐inflammatory abilities. While astilibin exhibits additional analgesic abilities, this should not be interpreted as an indication that the other three natural compounds lack analgesic properties because the data presented constitute a summary based on current literature. It is essential to highlight that, in this context, stereochemical structures appear to exert minimal influence compared to the impact of organic functional groups and their substitution sites in determining the antioxidant, anti‐inflammatory, and analgesic abilities of flavonoids. Nonetheless, intriguing researchers may explore the importance of stereochemical structures further.

Another crucial point to be emphasized in this paper is the growing interest in prenylated flavonoids, which have been analyzed extensively, underscoring the significance of this organic functional group in determining the anti‐inflammatory and analgesic properties of flavonoids. However, due to the limited discovery of prenylated flavonoids, further investigation into their biological capabilities has been restricted. In the future, it will be essential to either synthesize prenylated flavonoids or enhance extraction methods. This may include employing techniques such as supercritical fluid extraction, ultrasound‐assisted extraction, microwave‐assisted extraction, and separation methods like membrane separation techniques.

## Author Contributions

Conceptualization, methodology, and writing‐original draft: J.A.; Data acquisition: Z.Z., A.J., and M.T.; Funding acquisition and project administration: S.J.; Project administration, writing‐review and editing: Y.L. All authors have read and agreed to the published version of the manuscript.

## Conflicts of Interest

The authors declare no conflicts of interest.

## Supporting information


Appendix S1.

## Data Availability

The data that supports the findings of this study are openly available in Supporting Information.
