# Supplementary material for: Organic Functional Groups and Their Substitution Sites in Natural Flavonoids: A Review on Their Contributions to Antioxidant, Anti‐Inflammatory, and Analgesic Capabilities
Source: Food Sci Nutr. 2025 Apr 30;13(5):e70191. doi: 10.1002/fsn3.70191 (PMC12041660; doi:10.1002/fsn3.70191)
Supplement: Supplementary file 1 — Appendix S1. [file FSN3-13-e70191-s001.docx]

**Supplementary Materials**

**Methodology Section**

Extra Trees Classifier, Gradient Boosting Classifier, Extreme Gradient Boost (XGB), and Random Forest (RF) Classifier are among the most well-known feature selection methods (An et al., 2023). In this paper, feature selection methods were utilized to understand which substitution sites in flavonoids could characterize their antioxidant , anti-inflammatory, and analgesic abilities using in-house developed codes based on Python. The in-house developed codes employed the following open-source libraries: pandas, numpy, matplotlib, and scikit-learn (An et al., 2023).

During experiments, input data were sourced from Tables S8-S14, respectively, containing diverse organic functional groups such as hydroxyl, methoxy, prenylated, flavonoids, and glycosyl groups (monosaccharide, disaccharide, and trisaccharide), while output data were sourced from the same tables, containing diverse medicinal properties such as antioxidant, anti-inflammatory, or analgesic properties. Taking Table S8 as an example, to understand which substitution site (C3-C8 and C2’-C5’) is important for flavanols, the input data from catechin consisted of hydroxyl group, none, hydroxyl group, none, hydroxyl group, none, none, hydroxyl group, hydroxyl group, none, while the output data indicated analgesic ability. The results regarding the important substitution sites for flavonoids with diverse skeletons are listed in Figures S8-S14.

Table S1. The antioxidant, anti-inflammatory and analgesic abilities of flavanols.

| Name | Antioxidant (AO) | Anti-inflammatory ability (AI) | Analgesic ability (AG) |
| --- | --- | --- | --- |
| Catechin | ABTS, DPPH assay reaction (Lee et al., 2016) | COX, PGs, NO inhibition (Spagnuolo, Moccia, & Russo, 2018) | Inflammation-pain inhibition  (Komakech et al.,2019) |
| Epicatechin | DPPH assay reaction (Hernández-Rodríguez et al., 2019) | COX-2, iNOS and NF-κβ inhibition (Bouyahya et al., 2022) | Neuropathic pain inhibition  (Lim et al., 2022) |
| Gallocatechin | Oxidative stress inhibition (Someya et al., 2002) | NF-κβ inhibition (Vendidandala et al., 2021) | Chronic pain inhibition (Chopade et al., 2015) |
| Isomelacacidin | DPPH assay reaction (Hernández-Rodríguez et al., 2019) |  |  |
| Melacacidin | DPPH assay reaction (Hernández-Rodríguez et al., 2019) |  |  |
| Proanthocyanidin A1 | DPPH assay reaction (Hernández-Rodríguez et al., 2019) |  |  |
| Proanthocyanidin A6 | DPPH assay reaction (Hernández-Rodríguez et al., 2019) |  |  |
| Proanthocyanidin B2 | DPPH assay reaction (Hernández-Rodríguez et al., 2019) |  |  |
| Procyanidin A2 | ABTS+, DPPH assay reaction (Hernández-Rodríguez et al., 2019) |  |  |

*Note: AO=Antioxidant ability; AI=Anti-inflammatory ability; AG=Analgesic ability*

Table S2. The antioxidant, anti-inflammatory and analgesic abilities of anthocyanins (and anthocyanidins).

| Name | Antioxidant (AO) | Anti-inflammatory ability (AI) | Analgesic ability (AG) |
| --- | --- | --- | --- |
| Apigeninidin | Pro-oxidant activity inhibition (Carbonneau et al., 2014) | COX-2 and PGE_2_ inhibition (Makanjuola+ et al., 2018) |  |
| Callistephin | ABTS^+^, DMPD, DPPH and Fe^2+^ assay reaction (Huyut, Beydemir, & Gülçin, 2017) | TNF-α inhibition (Zhao et al., 2019) |  |
| Cyanidin | ABTS, DPPH, FRAP assay reaction (ZHANG, Peng, Xu, Lü, & Wang, 2016) | NF-κβ inhibition (Daveri et al., 2018) | Pain inhibition (Xiao et al., 2016) |
| Cyanidin-3-glucoside | ABTS, DPPH, FRAP assay reaction (ZHANG, Peng, Xu, Lü, & Wang, 2016) | COX-2 inhibition (Vezza et al., 2016) |  |
| Cyanidin-3,5-diglucosides | ABTS, DPPH, FRAP assay reaction (ZHANG, Peng, Xu, Lü, & Wang, 2016) | IL-6 inhibition (Mansoor et al., 2023) |  |
| Cyanidin-3-(6’’-malonylglucoside) | ABTS, DPPH, FRAP assay reaction (ZHANG, Peng, Xu, Lü, & Wang, 2016) |  |  |
| Delphinidin | ABTS, DPPH, FRAP assay reaction (ZHANG, Peng, Xu, Lü, & Wang, 2016) | iNOS, NO, IL-6, TNF-α, NF-κβ inhibition (Nagula & Wairkar, 2019) | Pain inhibition (Sauer et al., 2021) |
| Delphinidin 3,5-diglucosides | ABTS, DPPH, FRAP assay reaction (ZHANG, Peng, Xu, Lü, & Wang, 2016) |  |  |
| Malvin | ABTS^+^, DMPD, DPPH and Fe^2+^ assay reaction (Huyut, Beydemir, & Gülçin, 2017) |  |  |
| Malvidin-3-glucoside (Oenin) | ABTS^+^, DMPD, DPPH and Fe^2+^ assay reaction (Huyut, Beydemir, & Gülçin, 2017) | Pro-inflammatory genes inhibition (Huang et al., 2014) |  |

*Note: AO=Antioxidant ability; AI=Anti-inflammatory ability; AG=Analgesic ability*

Table S3. The antioxidant, anti-inflammatory and analgesic abilities of flavanones.

| Name | | Antioxidant (AO) | Anti-inflammatory ability (AI) | | Analgesic ability (AG) |
| --- | --- | --- | --- | --- | --- |
| Astilbin | ABTS^+^, DPPH and FRAP assay reaction (Zhao et al., 2020) | | IL-1β, IL-6, NO and NF-κβ inhibition (Zhao et al., 2020) | | Pain inhibition (Bi et al., 2019) |
| Neoastilbin | ABTS^+^, DPPH and FRAP assay reaction (Zhao et al., 2020) | | IL-1β, IL-6, NO and NF-κβ inhibition (Zhao et al., 2020) | |  |
| Neoisoastilbin | ABTS^+^, DPPH and FRAP assay reaction (Zhao et al., 2020) | | IL-1β, IL-6, NO and NF-κβ inhibition (Zhao et al., 2020) | |  |
| Isoastilbin | ABTS^+^, DPPH and FRAP assay reaction (Zhao et al., 2020) | | IL-1β, IL-6, NO and NF-κβ inhibition (Zhao et al., 2020) | |  |
| Brutieridin | Radical Scavenging activity (Bartella et al., 2022) | |  | |  |
| Dihydrokaempferol  (Aromadendrin) | DPPH assay (Hernández-Rodríguez et al., 2019) | | NF-κβ inhibition (Lee et al., 2013) | |  |
| Dihydromyricetin | ABTS+, DPPH assay inhibition (Zuo et al., 2018) | |  | |  |
| Eriodictyol | Oxidative stress inhibition (Zhu et al., 2015) | | IL-6, TNF-α inhibition (Silva et al., 2021) | |  |
| 3,7,8,3,4-pentahydroxydihydroflavone | ABST^+^ assay (Hernández-Rodríguez et al., 2019) | |  | |  |
| 3,4’5,7-tetrahydroxyflavanone | ABST^+^ assay reaction (Hernández-Rodríguez et al., 2019) | |  | |  |
| Hesperetin | ROS inhibition (Ginwala et al., 2019) | | COX, LOX inhibition (Nagula & Wairkar, 2019) | | Neuropatic pain inhibition (Aswar et al., 2014) |
| Hesperidin | Oxidative stress inhibition (Ahmadi & Shadboorestan, 2016) | | COX-2, iNOS, and NF-κβ inhibition (Spagnuolo et al., 2018) | | Acetic acid-induced model inhibition (Xiao et al., 2016) |
| Isocarthamidin-7-O-glucuronide | ABTS^+^, DPPH assay inhibition (Hernández-Rodríguez et al., 2019) | | Inflammation inhibition (Oracz et al., 2023) | |  |
| Japonicasins A | DPPH assay inhibition (Hernández-Rodríguez et al., 2019) | |  | |  |
| Japonicasins B | DPPH assay inhibition (Hernández-Rodríguez et al., 2019) | |  | |  |
| Kurarinone | Oxidative stress inhibition | | Inflammation inhibition Inflammation inhibition (Shi et al., 2021) | |  |
| 5-Methylsophoraflavanone | Oxidative stress inhibition | | Inflammation inhibition Inflammation inhibition (Shi et al., 2021) | |  |
| Melitidin | Radical Scavenging activity (Bartella et al., 2022) | |  | |  |
| Dihydromyricetin | Oxidative stress inhibition (Kirschweng et al., 2016) | | Inflammatory disease inhibition (Sun et al., 2022) | | Neuropathic pain inhibition (Ge et al., 2019) |
| Naringenin | DPPH assay inhibition (Hernández-Rodríguez et al., 2019) | | IL-6, TNF-α inhibition (Silva et al., 2021) | | Exerting analgesic ability by inhibiting inflammation (Komakech et al., 2019) |
| Naringin | ABTS^+^, DPPH assay inhibition (Mamdouh et al., 2004) | | Neuroinflammation inhibition (Liu et al., 2004) | | Neuropathic pain inhibition (Eom et al., 2021) |
| Naringenin-7-O-glucopyranoside | DPPH assay inhibition (Hernández-Rodríguez et al., 2019) | |  | |  |
| Neoeriocitrin | Radical scavenging activity (Bartella et al., 2022) | | Inflammation inhibition (Denaro et al., 2021) | |  |
| Neohesperidin | Radical scavenging activity (Bartella et al., 2022) | | Inflammation inhibition (Denaro et al., 2021) | | Neuropathic pain inhibition (Wang et al., 2023) |
| Peripolin | Radical scavenging activity (Bartella et al., 2022) | |  | |  |
| Name | Antioxidant (AO) | | Anti-inflammatory ability (AI) | | Analgesic ability (AG) |
| Pinocembrin | ABTS^+^ assay inhibition (Hernández-Rodríguez et al., 2019) | | LPS-induced inflammation inhibition (Soromou et al., 2012) | | Hip fracture-induced pain inhibition (Xing et al., 2022) |
| Pinobanksin | ROS inhibition (Xu et al., 2022) | | NF-κβ inhibition (Xu et al., 2022) | |  |
| Pinocembrin-7-O-β-D-glucopyranoside | Xanthine oxidase (XO) inhibition (Nile, Keum, Nile, Jalde, & Patel, 2018) | |  | |  |
| Sophoraflavanone G | Oxidative stress inhibition (Chen et al., 2014) | | Inflammation inhibition (Shi et al., 2021) | | Neuropathic pain inhibition (Calderon-Rivera, Loya-Lopez, Gomez, & Khanna, 2022) |
| Geranyl-5,7-dihydroxyl-3,4’-dimethoxyflavanone | Oxidative stress inhibition (Chen et al., 2014) | | Inflammation inhibition (Shi et al., 2021) | |  |
| 3’-O-Methyldiplacone | Oxidative stress inhibition (Chen et al., 2014) | | Inflammation inhibition (Shi et al., 2021) | |  |
| Diplacone | Oxidative stress inhibition (Chen et al., 2014) | | Inflammation inhibition (Shi et al., 2021) | |  |
| Tomentodiplacone N | Oxidative stress inhibition (Chen et al., 2014) | | Inflammation inhibition (Shi et al., 2021) | |  |
| Nymphaeol C | Oxidative stress inhibition (Chen et al., 2014) | | Inflammation inhibition (Shi et al., 2021) | |  |
| Isonymphaeol B | Oxidative stress inhibition (Chen et al., 2014) | | Inflammation inhibition (Shi et al., 2021) | |  |
| 3’-Geranylnaringenin | Oxidative stress inhibition (Chen et al., 2014) | | Inflammation inhibition (Shi et al., 2021) | |  |
| Kuwanon E | Oxidative stress inhibition (Chen et al., 2014) | | Inflammation inhibition (Shi et al., 2021) | |  |
| Paulownione A | Oxidative stress inhibition (Chen et al., 2014) | | Inflammation inhibition (Shi et al., 2021) | |  |
| Paulownione B | Oxidative stress inhibition (Chen et al., 2014) | | Inflammation inhibition (Shi et al., 2021) | |  |
| Sophoratonin F | Oxidative stress inhibition (Chen et al., 2014) | | Inflammation inhibition (Shi et al., 2021) | |  |
| Lonchocarpol A | Oxidative stress inhibition (Chen et al., 2014) | | Inflammation inhibition (Shi et al., 2021) | |  |
| Euchrenone A | Oxidative stress inhibition (Chen et al., 2014) | | Inflammation inhibition (Shi et al., 2021) | |  |
| Taxifolin | ABTS^+^ and DPPH assay inhibition (Hernández-Rodríguez et al., 2019) | | IL-6, TNF-α inhibition (Silva et al., 2021) | | Neuropathic pain inhibition (Alay et al., 2022) |
| Tomentodiplacone O | Oxidative stress inhibition (Hanáková et al., 2017) | | COX-1, COX-2, 5-LOX inhibition (Hanáková et al., 2017) | |  |
| 3',4'-O-dimethyl-5'-hydroxy-diplacone | Oxidative stress inhibition (Hanáková et al., 2017) | | COX-2 inhibition (Hanáková et al., 2017) | |  |
| Mimulone | Oxidative stress inhibition (Hanáková et al., 2017) | | COX-2 inhibition (Hanáková et al., 2017) | |  |
| 3'-O-methyl-5'-hydroxydiplacone | Oxidative stress inhibition (Hanáková et al., 2017) | | COX-2 inhibition (Hanáková et al., 2017) | |  |
| Fukugetin | Oxidative stress inhibition (Acuña et al., 2008) | | Inflammation inhibition (Luzzi et al., 1997) | | Formalin test model (Xiao et al., 2016) |
| Fukugeside | DPPH assay inhibition (Gontijo et al., 2012) | | | Inflammation inhibition (Luzzi et al., 1997) | Formalin test mode (Xiao et al., 2016) |
| Volkensiflavone | FRAP assay inhibition (Carrillo-Hormaza et al., 2016) | | | Inflammation inhibition (Luzzi et al., 1997) | Formalin test model (Xiao et al., 2016) |

*Note: AO=Antioxidant ability; AI=Anti-inflammatory ability; AG=Analgesic ability*

Table S4. The antioxidant, anti-inflammatory and analgesic abilities of flavonols

| Name | Antioxidant (AO) | Anti-inflammatory ability (AI) | Analgesic ability (AG) |
| --- | --- | --- | --- |
| Broussonol D | Oxidative stress inhibition (Chen et al., 2014) | Inflammation inhibition (Shi et al., 2021) |  |
| Cudraflavone B | Oxidative stress inhibition (Chen et al., 2014) | Inflammation inhibition (Shi et al., 2021) |  |
| 5,4'-Dihydroxy-3,7,3'-trimethoxyflavone | ABTS^+^, DPPH assay reaction (Hernández-Rodríguez et al., 2019) |  |  |
| 3,4'-Dimethoxy-5,7-dihydroxyflavone | ABTS^+^ assay reaction (Hernández-Rodríguez et al., 2019) |  |  |
| 5,4'-Dihydroxy-3,6,7,8,3'-pentamethoxyflavone | ABTS^+^, DPPH assay reaction (Hernández-Rodríguez et al., 2019) |  |  |
| 5,4'-Dihydroxy-3,6,7,8-tetramethoxyflavone | ABTS+, DPPH assay reaction (Hernández-Rodríguez et al., 2019) |  |  |
| 5,4'-Dihydroxy-3,6,7-trimethoxyflavone | ABTS^+^, DPPH assay reaction (Hernández-Rodríguez et al., 2019) |  |  |
| 5,6,3',4'-Tetrahydroxy-3,7-dimethoxyflavone | ABTS^+^, DPPH assay reaction (Hernández-Rodríguez et al., 2019) |  |  |
| 5,7,4'-Trihydroxy-3-(3-hydroxymethylbutyl)-3,6-dimethoxyflavone | ABTS^+^ assay reaction (Hernández-Rodríguez et al., 2019) |  |  |
| 5,7-Dihydroxy-3'-(4''-acetoxy-3''-methylbutenyl)-3,6,4'-trimethoxy-flavone | ABTS^+^ assay reaction (Hernández-Rodríguez et al., 2019) |  |  |
| 5,7-Dihydroxy-3'-(2-hydroxy-3-methyl-3-butenyl)-3,6,4-trimethoxy-flavone | ABTS^+^ assay reaction (Hernández-Rodríguez et al., 2019) |  |  |
| 5,7-Dihydroxy-3'-(3-hydroxymethylbutyl)-3,6,4-trimethoxyflavone | ABTS^+^ assay reaction (Hernández-Rodríguez et al., 2019) |  |  |
| 5-Hydroxy-3,6,7,8,3',4'-hexamethoxyflavone | ABTS^+^, DPPH assay reaction (Hernández-Rodríguez et al., 2019) |  |  |
| 7,4'-Dihydroxy-flavonol-3-O-α-glucoside | ABTS^+^, DPPH assay reaction (Hernández-Rodríguez et al., 2019) |  |  |
| 5,7,4'',5'',3''',4'''-Hexahydroxy-3''-O-β-glucosyl-3',7''-O-biflavone | DPPH assay inhibition reaction (Hernández-Rodríguez et al., 2019) |  |  |
| Falandioside B | ABTS+, DPPH assay reaction (Hernández-Rodríguez et al., 2019) |  |  |
| Fisetin | ROS inhibition (Ginwala et al., 2019) | COX-2, IL-6, IL-1β, iNOS, PEG_2_, TNF-α inhibition (Ginwala et al., 2019) | Pain inhibition (Xiao et al., 2016) |
| Galangin | ROS inhibition (Xu et al., 2022) | NF-κβ inhibition (Xu et al., 2022) |  |
| Gossypin | SOD inhibition (Song et al., 2023) | IL-1β, IL-6, TNF-α inhibition (Song et al., 2023) | Acetic acid-induced model (Xiao et al., 2016) |
| Icariin | Oxidative stress inhibition (Amanat et al., 2022) | TNF-α inhibition (Jucá et al., 2020) | Pain inhibition (Li et al., 2021) |
| Isorhamnetin | Radical scavenging activity (Filannino et al., 2016) | IL-1β, IL-6 inhibition (Silva et al., 2021) |  |
| Isorhamnetin 3-O-rutinoside | ABTS+, DPPH assay reaction (Chen et al., 2019) | COX-2, IL-1β and IL-6, NOS, PGE_2_ and TNF-α inhibition (Chen et al., 2019) |  |
| Kaempferol | DPPH assay reaction (Hernández-Rodríguez et al., 2019) | NF-κβ, IL-1β, IL-6 inhibition (Nagula & Wairkar, 2019; Silva et al., 2021) | Acetic acid-induced model (Xiao et al., 2016) |
| Kaempferol-3-methylether | ABTS^+^ assay reaction (Hernández-Rodríguez et al., 2019) |  |  |
| Kaempferol-3-O-[α-L-rhamnopyranosyl-(1-6)]-[(β-D-glucopyranosyl-(1-2)]-β-D-glucopyranoside | DPPH assay reaction (Hernández-Rodríguez et al., 2019) |  |  |
| Name | Antioxidant (AO) | Anti-inflammatory ability (AI) | Analgesic ability (AG) |
| Kaempferol-3-O-D-glucopyranosyl-(1→2)-L-rhamnopyranosyl-(1→6)-L-rhamnopyranoside | ABTS^+^, DPPH assay reaction (Hernández-Rodríguez et al., 2019) |  |  |
| Kaempferol-3-O-α-L-rhamnopyranosyl-(1-6)-β-D-glucopyranosyl-(1-2)-β-D-glucopyranoside | DPPH assay reaction (Hernández-Rodríguez et al., 2019) |  |  |
| Kaempferol 3-O-β-glucopyranosyl(1 → 2)-β-galactopyranoside-7-O-α-rhamnopyranoside | DPPH assay reaction (Hernández-Rodríguez et al., 2019) |  |  |
| Kaemferol-3-O-α-L-rhamnopyranosyl-7-O-[β-D-glucopyranosyl-(1-2)-O-L-rhamnoside] | ABTS^+^, DPPH assay reaction (Hernández-Rodríguez et al., 2019) |  |  |
| Kaempferol-3-O -glucose-7-O- rhamnoside | ABTS^+^, DPPH assay (Hernández-Rodríguez et al., 2019) |  |  |
| Kaempferol-3-O-α-rhamnoside | DPPH assay (Hernández-Rodríguez et al., 2019) |  |  |
| Kaemferol-3-O-β-D-glucoside (Astragalin) | DPPH assay reaction (Hernández-Rodríguez et al., 2019) | NO inhibition (Zhang et al., 2015) | Neuropathic pain inhibition (Wang et al., 2021) |
| Kaempferol-3-O-glucuronate  (Kamepferol-3-O-glucuronide) | Oxidative stress inhibition (Deng et al., 2021) | Carrageenan induced paw edema (Dogan et al., 2022) |  |
| Kaemferol-3-O-β-D-(6’’-methylglucuronide) | Oxidative stress inhibition (Zhao et al., 2021) |  |  |
| Kaempferol 3-O-robinobioside | ABTS^+^, DPPH assay reaction (Chen et al., 2019) | COX-2, IL-1β and IL-6, NOS, PGE_2_ and TNF-α inhibition (Chen et al., 2019) |  |
| 6,7-Dimethylkaempferol | ABTS^+^ assay inhibition (Hernández-Rodríguez et al., 2019) |  |  |
| Linarin | Oxidative stress inhibition (Mottaghipisheh et al., 2021) | IL-6, NO, PEG_2_, TNF-α inhibition (Mottaghipisheh et al., 2021) | Hot plate test model (Xiao et al., 2016) |
| Melanoxetin | DPPH assay inhibition (Lin et al., 2018) |  |  |
| Morin | Oxidative stress inhibition (Issac et al., 2021) | IL-6, IL-1β, iNOS and TNF-α (Rabidas et al., 2023) | Neuropathic pain inhibition (Basu & Basu, 2020) |
| Myricetin | DPPH assay reaction (Hernández-Rodríguez et al., 2019) | IL-1β, IL-8 inhibition (Silva et al., 2021) | Neuropathic pain inhibition (Hagenacker et al., 2010) |
| Myricetin-3-O-β-D-glucopyranoside | Oxidative stress inhibition (Wu et al., 2015) | Inflammation inhibition (Mangla et al., 2021) | Acetic acid-induced model (Xiao et al., 2016) |
| Paucatalinone E | DPPH assay inhibition (Hernández-Rodríguez et al., 2019) |  |  |
| Penduletin | ABTS^+^ assay inhibition (Hernández-Rodríguez et al., 2019) | LPS inhibition (Moscatelli et al., 2006) |  |
| Pectolinarin | Oxidative stress inhibition (Yoo et al., 2008) | IL-1β, IL-6 inhibition (Wang et al., 2019) | Hot plate test (Xiao et al., 2016) |
| Quercetin | ABTS^+^ assay, DPPH assay inhibition (Silva et al., 2021) | COX, LOX, IL-1β, IL-6, TNF-α and NO inhibition (Silva et al., 2021) | Hot plate test inhibition (Bouyahya et al., 2022) |
| Quercetin-3-O-arabinoside | ABTS^+^ assay, DPPH assay and FRAP assay inhibition (Zhu et al., 2015) |  |  |
| Quercetin-3-O-glucopyranoside  (Isoquercitrin) | ABTS^+^, DPPH assay reaction (Chen et al., 2019) | LOX inhibition (Silva et al., 2021) |  |
| Quercetin-3-O-β-D-glucuronide | ABTS^+^, DPPH assay reaction (Hernández-Rodríguez et al., 2019) |  |  |
| Quercetin-3-O-xyloside | PMS/NADH-NBT assay reaction (Dong et al., 2019) | NF-κβ inhibition (Lee et al., 2016) |  |
| Name | Antioxidant (AO) | Anti-inflammatory ability (AI) | Analgesic ability (AG) |
| Quercimeritrin | DPPH assay inhibition (Ahmed et al., 2016) | NO inhibition (Anuradha & Sukumar, 2013) |  |
| Quercetin-3-O-D-glucopyranosyl-(1→2)-L-rhamnopyranosyl-(1→6)-L-rhamnopyranoside | ABTS^+^, DPPH assay reaction (Hernández-Rodríguez et al., 2019) |  |  |
| Quercetin-3-methoxy-4-glucosyl-7-glucoside | Oxidative stress inhibition (Mondal et al., 2019) | Carrageenan induced paw edema (Mondal et al., 2019) | Acetic acid-induced writhing model (Mondal et al., 2019) |
| Quercitrin | DPPH assay reaction (Hernández-Rodríguez et al., 2019) | Carrageenan induced paw edema (Zhao et al., 2018) | Acetic acid test (Zhao et al., 2018) |
| Quercetin-3-O-rutinoside (Rutin) | ABTS^+^, DPPH, FRAP assay reaction (Lee et al., 2016) | TNF-α inhibition (Silva et al., 2021) | Glutamate-induced pain model (Xiao et al., 2016) |
| 5-O-Methylquercetin  (Azaleatin) | Oxidative stress inhibition (Tsai et al., 2022) | Inflammation inhibition (Tsai et al., 2022) | Acetic acid-induced model (Xiao et al., 2016) |
| Quercetin 3,7-O-α-dirhamnoside | Oxidative stress inhibition (Toker et al., 2004) | Carrageenan induced paw edema (Toker et al., 2004) | Writhing test model (Xiao et al., 2016) |
| α-Rhamnoisorobin | H_2_O_2_-induced cell damage inhibition (Jantas et al., 2021) | NO inhibition (Rho et al., 2011) | Acetic acid-induced writhing test model (Xiao et al., 2016) |
| Santin | ABTS^+^ assay reaction (Hernández-Rodríguez et al., 2019) |  |  |
| Transilitin | DPPH assay reaction (Hernández-Rodríguez et al., 2019) |  |  |
| Viscosine | ABTS^+^ assay reaction (Hernández-Rodríguez et al., 2019) | iNOS inhibition (Ali et al., 2016) | Pain inhibition (Amir et al., 2014) |

*Note: AO=Antioxidant ability; AI=Anti-inflammatory ability; AG=Analgesic ability*

Table S5. The antioxidant, anti-inflammatory and analgesic abilities of isoflavones.

| Name | Antioxidant (AO) | Anti-inflammatory ability (AI) | Analgesic ability (AG) |
| --- | --- | --- | --- |
| Biochanin-A-7-O-glucoside | ABTS^+^, DPPH assay inhibition (Hernández-Rodríguez et al., 2019) |  |  |
| Daidzein | Oxidative stress inhibition (Shimoda et al., 2011) | IL-6 and iNOS inhibition (Spagnuolo et al., 2018) | Paclitaxel-induced neuropathic pain inhibition (Zafar et al., 2023) |
| Formononetin | SOD inhibition (Ma, Ji, Fu, & Ma, 2013) | IL-6 and TNF-α inhibition (Ma, Ji, Fu, & Ma, 2013) | Inflammation-pain inhibition (Wang et al., 2019) |
| Genistein | ROS inhibition (Zielonka, Gębicki, & Grynkiewicz, 2003) | IL-6, COX-2 inhibition (Silva et al., 2021) | Inhibition of voltage-gated Na+channels in the nociceptive nerve terminals of trigeminal ganglion (Spagnuolo et al., 2018). |
| Glabridin | Oxidative stress inhibition (Kang et al., 2015) | NO inhibition (Vezza et al., 2016) | Pain inhibition (Ali et al., 2020) |
| Glycitein | Oxidative stress inhibition (Gutierrez-Zepeda et al., 2005) | LPS, NF-κβ inhibition (Lee et al., 2010) | Pain inhibition (Glabska et al., 2017) |
| Glyurallin B | Oxidative stress inhibition (Fu et al., 2013) |  |  |
| Orobol-7-O- glucoside | ABTS+, DPPH assay inhibition (Hernández-Rodríguez et al., 2019) | Inflammation inhibition (Morel et al., 2017) |  |
| Scandenone (warangalone) | Oxidative stress inhibition (Yao et al., 2021) | Carrageenan induced paw edema inhibition (Kupeli et al., 2006) | ρ-benzoquinone-induced writhing model inhibition (Xiao et al., 2016) |
| Echinoisoflavanone | Oxidative stress inhibition (Chen et al., 2014) | Inflammation inhibition (Shi et al., 2021) |  |
| Echinoisosophoranone | Oxidative stress inhibition (Chen et al., 2014) | Inflammation inhibition (Shi et al., 2021) |  |
| Isosophoranone | Oxidative stress inhibition (Chen et al., 2014) | Inflammation inhibition (Shi et al., 2021) |  |
| 5,7,4’-Trihydroxy-6,8-diprenylisoflavone | Oxidative stress inhibition (Chen et al., 2014) | Inflammation inhibition (Shi et al., 2021) |  |
| Warangalone | Oxidative stress inhibition (Chen et al., 2014) | Inflammation inhibition (Shi et al., 2021) |  |
| Gancaonin A | Oxidative stress inhibition (Chen et al., 2014) | Inflammation inhibition (Shi et al., 2021) |  |
| Isoerysenegalensein E | Oxidative stress inhibition (Chen et al., 2014) | Inflammation inhibition (Shi et al., 2021) |  |
| 7-methoxyebenosin | Oxidative stress inhibition (Chen et al., 2014) | Inflammation inhibition (Shi et al., 2021) |  |
| Ficucaricone D | Oxidative stress inhibition (Chen et al., 2014) | Inflammation inhibition (Shi et al., 2021) |  |
| 4’-Hydroxy-5,7-dimethoxy-6-(3-methyl-2-butenyl)-isoflavone | Oxidative stress inhibition (Chen et al., 2014) | Inflammation inhibition (Shi et al., 2021) |  |
| Gancaonin N | Oxidative stress inhibition (Chen et al., 2014) | Inflammation inhibition (Shi et al., 2021) |  |
| Isopiscerythrone | Oxidative stress inhibition (Chen et al., 2014) | Inflammation inhibition (Shi et al., 2021) |  |
| Viridiflflorin | Oxidative stress inhibition (Chen et al., 2014)` | Inflammation inhibition (Shi et al., 2021) |  |

*Note: AO=Antioxidant ability; AI=Anti-inflammatory ability; AG=Analgesic ability*

Table S6. The antioxidant, anti-inflammatory and analgesic abilities of flavones

| Name | Antioxidant (AO) | Anti-inflammatory ability (AI) | Analgesic ability (AG) |
| --- | --- | --- | --- |
| Acacetin | Oxidative stress inhibition (Khan et al., 2021) | Anti-inflammatory (Ginwala et al., 2019) | Analgesic ability (Ginwala et al., 2019) |
| Amentoflavone | Oxidative stress inhibition (Bajpai et al., 2019) | IL-1β, IL-6 and PGE_2_ inhibition (Abdallah et al., 2015) | Acetic acid-induced writhing test inhibition (Xiao et al., 2016) |
| Apiin | Antioxidant (Ginwala, Bhavsar, Chigbu, Jain, & Khan, 2019) | NO inhibition (Mencherini et al., 2007) |  |
| Apigenin | DPPH assay inhibition (Hernández-Rodríguez et al., 2019) | iNOS, COX-2, LOX and NF-κβ inhibition (Nagula & Wairkar, 2019; Silva et al., 2021) | Exerting analgesic ability by inhibiting inflammation (Komakech et al.,2019) |
| Apigenin-7-O-β-D- (6''-ρ-coumaroly) glucopyranoside (APG) | Oxidative stress inhibition (Khan et al., 2021) | IL-1β, IL-6 and PGE_2_ inhibition (Abdallah et al., 2015) |  |
| Apigenin-7-O-glucoside | ABTS^+^, DPPH assay inhibition (Hernández-Rodríguez et al., 2019) | IL-6 and TNF-α inhibition (Wang et al., 2020) |  |
| Apigenin-7-O-glucuronide | ABTS^+^, DPPH assay inhibition (Hernández-Rodríguez et al., 2019) | IL-1β, IL-6, NO and TNF-α inhibition (Feng et al., 2019) | Acetic acid-induced writing and formalin-induced test inhibition (Feng et al., 2019) |
| Apigenin-8-C-glucoside  (Vitexin) | ABTS^+^, DPPH assay inhibition (Lee et al., 2016) | COX-2, IL-1β, IL-6, IL-8, IL-17, IL-33, TNF-α, NF-κβ, iNOS, NO, PGE_2_ inhibition (He et al., 2016) | Inflammatory pain inhibition (Borghi et al., 2013) |
| Artogomezianone | ABTS^+^, DPPH assay inhibition (Hernández-Rodríguez et al., 2019) |  |  |
| Baicalein | ABTS^+^, DPPH assay inhibition (Hernández-Rodríguez et al., 2019) | NF-κβ (Chen et al., 2018) | Neuropathic pain inhibition (Lai et al., 2018) |
| Baicalein-7-O-gentiobioside  (Oroxin B; Baicalin-7-diglucoside) | DPPH assay inhibition (Hernández-Rodríguez et al., 2019) | COX-2, IL-6, IL-1β, iNOS and TNF-α,  (Lu et al., 2022) |  |
| Baicalein-7-O-glucoside | DPPH assay inhibition (Yan, Cao, & Yang, 2014) |  |  |
| Baicalin | DPPH assay inhibition (Hernández-Rodríguez et al., 2019) | Carrageenan induced paw edema (Xiao et al., 2016) | Neuropathic pain inhibition (Cherng et al., 2014) |
| Bilobetin | SOD inhibition (Negm, El-Kadem, Hussein, & Alqahtani, 2022) | Inflammation inhibition ((Negm et al., 2022) |  |
| Carambolaflavone | ABTS^+^, DPPH assay inhibition (Hernández-Rodríguez et al., 2019) |  |  |
| Chrysin | ABTS^+^, DPPH assay inhibition (Silva et al., 2021) | IL-6, IL-12, NF-κβ, TNF-α inhibition (Zeinali et al., 2017) | Formalin test (Zakaria et al., 2015) |
| Chrysin-7-O-glucuronide | ABTS^+^ assay inhibition (Hernández-Rodríguez et al., 2019) |  |  |
| Chrysoeriol | H_2_O_2_-induced oxidative stress inhibition (Kim, Kwon, Woo, Seo, & Kim, 2021) | NF-κβ inhibition (Wu et al., 2020) |  |
| Cosmosiin | ABTS^+^ assay inhibition (Hernández-Rodríguez et al., 2019) |  |  |
| Diosmetin | H_2_O_2_-induced oxidative stress inhibition (Wójciak et al., 2022) | TNF-α, IL-1β, IL-6, iNOS, and NF-κβ inhibition (Ribeiro et al., 2015) | Analgesic ability inhibition (Adamante et al., 2019) |
| Diosmetin 7-O-glucopyranoside | ABTS^+^ assay inhibition (Hernández-Rodríguez et al., 2019) |  |  |
| 3-C-glucopyranosylapigenin | ABTS^+^, DPPH assay inhibition  (Hernández-Rodríguez et al., 2019) |  |  |
| Vitexin-2-O-xyloside | ROS inhibition (Ninfali, Antonini, Frati, & Scarpa, 2017) | COX-2 inhibition (Ninfali, Antonini, Frati, & Scarpa, 2017) |  |
| Vitexin-2’’-O-rhamnoside | FRAP assay inhibition (Alirezalu et al., 2018) |  |  |
| Name | Antioxidant (AO) | Anti-inflammatory ability (AI) | Analgesic ability (AG) |
| 5,4'-Dihydroxy-6,7,3'-trimethoxylflavone  (Cirsillineol) | ABTS^+^, DPPH assay inhibition (Hernández-Rodríguez et al., 2019) |  |  |
| 5,6, 4',-Trihydroxy-7, 3'-dimethoxyflavone | ROS inhibition (Wang et al., 2016) | COX-2, NF-κβ inhibition (Wang et al., 2016) |  |
| Isoscoparin 2''-O-(6'''-(E)-feruloyl)-glucopyranoside | DPPH assay inhibition (Yang et al., 2016) | NO inhibition (Yang et al., 2016) |  |
| Isoscoparin 2''-O-(6''''-(E)-ρ-coumaroyl)-glucopyranoside | DPPH assay inhibition (Yang et al., 2016) | NO inhibition (Yang et al., 2016) |  |
| Isocycloartobiloxanthone | ABTS^+^, DPPH assay inhibition (Lan, Tzeng, Lin, Yen, & Ko, 2013) |  |  |
| Isoorientin | ABTS^+^, DPPH assay inhibition (Lee et al., 2016) | COX inhibition (Chen et al., 2019) |  |
| Isovitexin | ABTS^+^, DPPH assay inhibition (Lee et al., 2016) | COX-2, IL-1β, IL-6, IL-8, IL-17, IL-33, TNF-α, NF-κβ, iNOS, NO, PGE_2_ inhibition (He et al., 2016) |  |
| Isovitexin-2''-O-α-L-rhamnopyranoside | ABTS^+^, DPPH assay inhibition (Lee et al., 2016) |  |  |
| Isovitexin 2''-O-(6'''-(E)-feruloyl)-glucopyranoside | DPPH assay inhibition (Yang et al., 2016) | NO inhibition (Yang et al., 2016) |  |
| GB-1a | DPPH assay inhibition (Aravind et al., 2016) | Carrageenan induced paw edema (Michel et al., 2016) | Acetic acid-induced writhing test (Xiao et al., 2016) |
| GB-2a | DPPH assay inhibition (Aravind et al., 2016) | Carrageenan induced paw edema (Michel et al., 2016) | Acetic acid-induced writhing test (Xiao et al., 2016) |
| Ginkgetin | Oxidative stress inhibition (Zhang et al., 2017) | Prostaglandin D_2_ inhibition (Chen et al., 2018) | Acetic acid-induced writhing test (Xiao et al., 2016) |
| Hispidulin | ABTS^+^, FRAP assay inhibition (Kut et al., 2022) | IL-1β, IL-6, and TNF-α inhibition (Rabidas et al., 2023) |  |
| Homoplantaginin | ROS inhibition (Zhang & Tsao, 2016) | IL-1β inhibition (Zhang & Tsao, 2016) |  |
| Hyperoside | Oxidative stress inhibition (Khan et al., 2021) | NF-κβ inhibition (Kim, Um, Hong, & Lee, 2011) |  |
| Luteolin | DPPH assay inhibition (Kiani et al., 2021) | IL-6, IL-1β inhibition (Silva et al., 2021) | Acetic acid-induced writhing and formalin tests (Zakaria et al., 2015) |
| Luteolin 6-C-glucoside | ABTS^+^, DPPH assay inhibition (Chen et al., 2019) | COX-2, IL-1β and IL-6, NOS, PGE_2_ and TNF-α inhibition (Chen et al., 2019) |  |
| Luteoloside | Oxidative stress inhibition (Sanghyun et al., 2012) | NF-κβ inhibition (Li et al., 2019) |  |
| 6-OH-luteolin-7-O-β-D-glucoside | ABTS^+^, DPPH assay inhibition (Hernández-Rodríguez et al., 2019) |  |  |
| Luteolin-7-O-glucoside | ABTS^+^, DPPH assay inhibition (Hernández-Rodríguez et al., 2019) | NF-κβ inhibition (Park & Song, 2019) | ρ-benzoquinone-induced abdominal constriction test model inhibition (Xiao et al., 2016) |
| Luteolin-7-O-glucopyranoside | ABTS^+^, DPPH assay inhibition (Hernández-Rodríguez et al., 2019) |  |  |
| Luteolin 7-O-neohesperidoside | ABTS+, DPPH assay (Chen et al., 2019) | COX-2, IL-1β and IL-6, NOS, PGE_2_ and TNF-α inhibition (Chen et al., 2019) |  |
| Luteolin 3'-O-glucoside | DPPH assay inhibition (Xu, Wang, Jiang, Yang, & Wu, 2022) | Carrageenan induced paw edema (Tatli, Akdemir, Yesilada, & Küpeli, 2008) | ρ-benzoquinone-induced abdominal constriction test model inhibition (Xiao et al., 2016) |
| Name | Antioxidant (AO) | Anti-inflammatory ability (AI) | Analgesic ability (AG) |
| Luteolin 4'-O-neohesperidoside | ABTS^+^ assay inhibition (Choucry et al., 2021) | Carrageenan induced paw edema (Ramesh et al., 1998) | ρ-benzoquinone-induced abdominal constriction test model inhibition (Xiao et al., 2016) |
| Morusin | DPPH assay inhibition (Zoofishan et al., 2018) | COX-2 inhibition (Jia et al., 2020) | Acetic acid-induced pain inhibition (Wang et al., 2017) |
| Nobiletin | SOD inhibition (Zhang et al., 2016) | NO inhibition (Chen et al., 2017) |  |
| Orientin | ABTS^+^, DPPH assay inhibition (Lee et al., 2016) | IL-1β, NF-κβ, TNF-α (Lee et al., 2014) | Acetic acid induced pain inhibition (Lam et al., 2016) |
| Oroxylin A | Oxidative stress inhibition (Li et al., 2016) | IL-1β, TNF-α inhibition (Li et al., 2016) |  |
| Paucatalinone C | DPPH assay inhibition (Hernández-Rodríguez et al., 2019) |  |  |
| Paucatalinone D | DPPH assay inhibition (Hernández-Rodríguez et al., 2019) |  |  |
| Rhamnetin | Oxidative stress inhibition (Ramešová et al., 2017) | Thromaboxane B_2_ inhibition (Chen et al., 2018) |  |
| Rhoifolin | Oxidative stress inhibition (Peng et al., 2020) | Inflammation inhibition (Ginwala et al., 2019) |  |
| Robustaflavone | DPPH assay inhibition (Hernández-Rodríguez et al., 2019) | IL-1β, IL-6, COX-2, iNOS, NF-κβ inhibition (Jo et al., 2019) |  |
| Schaftoside | Oxidative stress inhibition (Chen et al., 2015) | IL-1β, IL-6 and NF-κβ inhibition (Rabidas et al., 2023) |  |
| Scutellarein | DPPH assay inhibition (Ahmed et al., 2016) | NF-κβ inhibition (Park et al., 2022) |  |
| Scutellarin | Oxidative stress inhibition (Khan et al., 2021) | NF-κβ inhibition (Peng et al., 2020) | Cancer-induced bone pain inhibition (Liu et al., 2019) |
| Scutellarein-7-O-gentiobioside | DPPH assay inhibition (Hernández-Rodríguez et al., 2019) |  |  |
| Scutellarein-7-O-glucoside | DPPH assay inhibition (Hernández-Rodríguez et al., 2019) | Lung inflammation inhibition (Dinda et al., 2015) |  |
| Tangeritin | Oxidative stress inhibition (Khan et al., 2021) | NO inhibition (Chen, Tait, & Kitts, 2017) |  |
| Tricin | DPPH assay (Yang et al., 2016) | NO inhibition (Yang et al., 2016) |  |
| Tricin-5-O-β-D-glucopyranoside | DPPH assay (Yang et al., 2016) | NO inhibition (Yang et al., 2016) |  |
| Tricin-7-O-β-D-glucopyranoside | DPPH assay (Yang et al., 2016) | NO inhibition (Yang et al., 2016) |  |
| Tricin 7-rutinoside | DPPH assay (Yang et al., 2016) | NO inhibition (Yang et al., 2016) |  |
| Wogonin | Oxidative stress inhibition (Khan et al., 2021) | IL-1β, NF-κβ, TNF-α inhibition (Rabidas et al., 2023) | Neuropathic pain inhibition (Chen et al., 2015) |

*Note: AO=Antioxidant ability; AI=Anti-inflammatory ability; AG=Analgesic ability*

Table S7. The antioxidant, anti-inflammatory and analgesic abilities of chalcones

| Name | Antioxidant (AO) | Anti-inflammatory ability (AI) | Analgesic ability (AG) |
| --- | --- | --- | --- |
| Carambolasides A | ABTS^+^, DPPH assay inhibition (Hernández-Rodríguez et al., 2019) |  |  |
| Carambolasides C | ABTS^+^, DPPH assay inhibition (Hernández-Rodríguez et al., 2019) |  |  |
| Cardamomin (Cardamonin) | Oxidative stress inhibition by inducting antioxidant enzyme (De Spirt et al., 2016) | NF-κβ inhibition (Vezza et al., 2016) |  |
| 2’,4’-Dihydroxychalcone | Oxidative stress inhibition (Jiang, Zhan, Liu, & Jiang, 2008) | NO inhibition (Zhang et al., 2020) |  |
| Echinatin | ABTS^+^, DPPH assay inhibition (Hernández-Rodríguez et al., 2019) | Liver inflammation inhibition (Xu et al., 2021) |  |
| Hesperidin methyl chalcone (HMC) | Oxidative stress inhibition (Pinho-Ribeiro et al., 2015) | IL-1β, IL-6, IL-10, NF-κβ, TNF-α inhibition (Pinho-Ribeiro et al., 2015) | Inflammatory pain inhibition (Pinho-Ribeiro et al., 2015) |
| Kuraridin | Oxidative stress (Hyun et al., 2008) | IL-1β, IL-6, TNF-α (Chan et al., 2023) |  |
| Licochalcone A | ABTS^+^ and DPPH assay inhibition (Hernández-Rodríguez et al., 2019) | LPS induced inflammation inhibition (Chu et al., 2012) | Neuropathic pain inhibition (Li et al., 2021) |
| Licochalcone B | ABTS^+^ and DPPH assay inhibition (Hernández-Rodríguez et al., 2019) | NF-κβ inhibition (Furusawa et al., 2009) |  |
| 5-(1,1-Dimethylallyl)-3,4,4-trihydroxy-2-methoxychalcone | ABTS^+^, DPPH assay inhibition (Hernández-Rodríguez et al., 2019) | IL-6, NO, PGE_2_ inhibition (Fu et al., 2013) |  |
| Okanin | DPPH assay inhibition (Hernández-Rodríguez et al., 2019) | NF-κβ inhibition (Hou et al., 2017) |  |
| Phloretin rutinoside | Oxidation inhibition (Wang et al., 2019) | Inflammation inhibition (Habtemariam, 2023) |  |
| Isoliquiritigenin | ROS inhibition (Khan et al., 2021) | NF-κβ, TNF-α inhibition (Choy et al., 2019) | Pain inhibition (Shi et al., 2012) |
| Sophoradochromene | Oxidative stress inhibition (Chen et al., 2014) | Inflammation inhibition (Shi et al., 2021) |  |
| Isobacachalcone | Oxidative stress inhibition (Chen et al., 2014) | Inflammation inhibition (Shi et al., 2021) |  |
| Morachalcone A | Oxidative stress inhibition (Chen et al., 2014) | Inflammation inhibition (Shi et al., 2021) |  |
| Xanthohumol | ROS inhibition (Schempp et al., 2010) | COX-2, iNOS inhibition (Fernández et al., 2021) | Neuropathic pain inhibition (Jiang et al., 2016) |

*Note: AO=Antioxidant ability; AI=Anti-inflammatory ability; AG=Analgesic ability*

Table S8. The organic functional groups in flavanols’ substitution sites.

| Name | C3 | C4 | C5 | C6 | C7 | C8 | C2’ | C3’ | C4’ | C5’ | Medicinal Properties |
| --- | --- | --- | --- | --- | --- | --- | --- | --- | --- | --- | --- |
| Catechin | OH | None | OH | None | OH | None | None | OH | OH | None | AO, AI, AG |
| Epicatechin | OH | None | OH | None | OH | None | None | OH | OH | None | AO, AI, AG |
| Gallocatechin | OH | None | OH | None | OH | None | None | OH | OH | OH | AO, AI, AG |
| Isomelacacidin | OH | OH | 0 | None | OH | OH | None | OH | OH | None | AO |
| Melacacidin | OH | OH | 0 | None | OH | OH | None | OH | OH | None | AO |
| Proanthocyanidin B2 | OH | 0 | OH | None | OH | Flavonoid | None | OH | OH | None | AO |
| Proanthocyanidin A6 | OH | 0 | OH | Flavonoid | OH | Flavonoid | None | OH | OH | None | AO |
| Procyanidin trimer C1 | OH | Flavonoid | OH | None | OH | Flavonoid | None | OH | OH | None | AO |

Table S9. The organic functional groups in anthocyanins’ substitution sites

| Name | C3 | C4 | C5 | C6 | C7 | C8 | C2’ | C3’ | C4’ | C5’ | Medicinal Properties |
| --- | --- | --- | --- | --- | --- | --- | --- | --- | --- | --- | --- |
| Apigenindin | None | None | OH | None | OH | None | None | None | OH | None | AO, AI |
| Callistephin | Monosaccharide | None | OH | None | OH | None | None | None | OH | None | AO, AI |
| Cyanidin | OH | None | OH | None | OH | None | None | OH | OH | None | AO, AI. AG |
| Cyanindin-3-glucoside | Monosaccharide | None | OH | None | OH | None | None | OH | OH | None | AO, AI |
| Cyanindin-3,5-diglucosides | Monosaccharide | None | Monosaccharide | None | OH | None | None | OH | OH | None | AO, AI |
| Cyanidin-3-(6’’-malonylglucoside) | Monosaccharide | None | OH | None | OH | None | None | OH | OH | None | AO, AI, AG |
| Delphinidin | OH | None | OH | None | OH | None | None | OH | OH | OH | AO, AI, AG |
| Delphinidin-3,5-diglucosides | Monosaccharide | None | Monosaccharide | None | OH | None | None | OH | OH | OH | AO |
| Malvin | Monosaccharide | None | Monosaccharide | None | OH | None | None | OMe | OH | OMe | AO |
| Malvidin-3-glucoside | Monosaccharide | None | OH | None | OH | None | None | OMe | OH | OMe | AO |

Table S10. The organic functional groups in flavanones’ substitution sites

| Name | C3 | C5 | C6 | C7 | C8 | C2’ | C3’ | C4’ | C5’ | Medicinal Properties |
| --- | --- | --- | --- | --- | --- | --- | --- | --- | --- | --- |
| Astilbin | Monosaccharide | OH | None | OH | None | None | OH | OH | None | AO, AI, AG |
| Neoastilbin | Monosaccharide | OH | None | OH | None | None | OH | OH | None | AO, AI |
| Neoisoastilbin | Monosaccharide | OH | None | OH | None | None | OH | OH | None | AO, AI |
| Isoastilbin | Monosaccharide | OH | None | OH | None | None | OH | OH | None | AO, AI |
| Aromadendrin | OH | OH | None | OH | None | None | None | OH | None | AO |
| Brutieridin | None | OH | None | Monosaccharide | None | None | OH | OMe | None | AO, AI |
| Eriodictyol | None | OH | None | OH | None | None | OH | OH | None | AO, AI |
| 3,7,8,3’,4’-pentahydroxydihydroflavone | OH | None | None | OH | OH | None | None | OH | None | AO |
| 3, 4’ 5, 7-tetrahydroxyflavanone | OH | OH | None | OH | None | None | None | OH | None | AO |
| Hesperetin | None | OH | None | OH | None | None | OH | OMe | None | AO, AI, AG |
| Hesperidin | None | OH | None | Disaccharide | None | None | OH | OMe | None | AO, AI, AG |
| Isocarthamidin-7-O-glucuronide | None | OH | None | Monosaccharide | None | None | None | OH | None | AO, AI |
| Japonicasins A | None | OH | Prenyl | OH | None | None | OH | None | OH | AO |
| Japonicasins B | None | OH | Prenyl | OH | None | None | None | OH | None | AO |
| Kurarinone | None | OMe | None | OH | Prenyl | OH | None | OH | None | AO, AI |
| 5-Methylsophoraflavanone B | None | OMe | None | OH | Prenyl | None | None | OH | None | AO, AI |
| Meltidin | None | OH | None | Disaccharide | None | None | None | OH | None | AO |
| Dihydromyricetin | OH | OH | None | OH | None | None | OH | OH | OH | AO, AI, AG |
| Naringenin | None | OH | None | OH | None | None | None | OH | None | AO, AI, AG |
| Naringin | None | OH | None | Disaccharide | None | None | None | OH | None | AO, AI, AG |
| Naringenin-7-O-glucopyranoside | None | OH | None | Monosaccharide | None | None | None | OH | None | AO |
| Neoeriocitrin | None | OH | None | Disaccharide | None | None | OMe | OH | None | AO, AI |
| Neohesperidin | None | OH | None | Disaccharide | None | None | OMe | OH | None | AO, AI, AG |
| Peripolin | None | OH | None | Disaccharide | None | None | OH | OH | None | AO |
| Pinocembrin | None | OH | None | OH | None | None | None | None | None | AO, AI, AG |
| Pinobanksin | OH | OH | None | OH | None | None | None | None | None | AO, AI |
| Pinocembrin-7-O-glucopyranoisde | None | OH | None | Monosaccharide | None | None | None | None | None | AO |
| Sophoraflavanone G | None | OH | None | OH | Prenyl | OH | None | OH | None | AO, AI |
| Geranyl-,5,7-dihydroxyl-3’,4’-dimethoxyflavanone | None | OH | Prenyl | OH | None | None | OMe | OMe | None | AO, AI |
| 3’-O-methyldiplacone | None | OH | Prenyl | OH | None | None | OMe | OH | None | AO, AI |
| Diplacone | None | OH | Prenyl | OH | None | None | OH | OH | None | AO, AI |
| Taxifolin | OH | OH | None | OH | None | None | OH | OH | None | AO, AI, AG |
| Tomentodiplacone O | None | OH | Prenyl | Prenyl | None | None | OMe | OH | None | AO, AI |
| 3’,4’-dimethyl-5’-hydroxy-diplacone | None | OH | Prenyl | OH | None | None | OMe | OMe | OH | AO, AI |
| Tomentodiplacone N | None | OH | Prenyl | Prenyl | None | None | OMe | OH | None | AO, AI |
| Paulownione C | None | OH | Prenyl | Prenyl | None | None | OMe | OH | OH | AO, AI |
| Nymphaeol A | None | OH | Prenyl | OH | None | None | OH | OH | None | AO, AI |
| Nymphaeol B | None | OH | None | OH | None | Prenyl | OH | OH | None | AO, AI |
| Nymphaeol C | None | OH | Prenyl | OH | None | Prenyl | OH | OH | None | AO, AI |
| Isonymphaeol B | None | OH | None | OH | None | None | Prenyl | OH | OH | AO, AI |
| 3’-Geranylnaringenin | None | OH | None | OH | None | None | Prenyl | OH | None | AO, AI |
| Name | C3 | C5 | C6 | C7 | C8 | C2’ | C3’ | C4’ | C5’ | Medicinal Properties |
| Kuwanon E | None | OH | None | OH | None | OH | None | OH | Prenyl | AO, AI |
| Paulowinone A | OH | OH | Prenyl | OH | Prenyl | None | Prenyl | OH | Prenyl | AO, AI |
| Sophoratonin F | None | None | Prenyl | OH | Prenyl | None | None | OH | Prenyl | AO, AI |
| Lonchocarpol A | None | OH | Prenyl | OH | Prenyl | None | None | OH | None | AO, AI |
| Euchrenone A | None | OH | None | OH | Prenyl | OH | None | OH | None | AO, AI |
| Mimulone | None | OH | Prenyl | OH | None | None | None | OH | None | AO, AI |
| 3’-O-methyl-5’-hydroxydiplacone | None | OH | Prenyl | OH | None | None | OMe | OH | OMe | AO, AI |
| Fukugetin | None | OH | None | OH | Flavonoid | None | OH | OH | None | AO, AI, AG |
| Fukugeside | None | OH | None | Monosaccharide | Flavonoid | None | OH | OH | None | AO, AI, AG |
| Volkensiflavone | None | OH | None | OH | Flavonoid | None | None | OH | None | AO, AI |
| Neougonin A | None | OH | Prenyl | OH | None | None | OH | OH | None | AO, AI |

Table S11. The organic functional groups in flavonols’ substitution sites

| Name | C3 | C5 | C6 | C7 | C8 | C2’ | C3’ | C4’ | C5’ | Medicinal Properties |
| --- | --- | --- | --- | --- | --- | --- | --- | --- | --- | --- |
| Broussonol D | OH | OH | None | OH | Prenyl | None | OH | OH | Prenyl | AO, AI |
| Cudraflavone B | Prenyl group | OH | Prenyl | Prenyl | None | OH | None | OH | None | AO, AI |
| 5,4’-dihydroxy-3,7,3’-trimethoxyflavaone | OMe | OH | None | OMe | None | None | OMe | OH | None | AO |
| 3,4’-Dimethoxy-5,7-dihydroxyflavone | OMe | OH | None | OH | None | None | None | OMe | None | AO |
| 5,4’-Dihydroxy-3,6,7,8,3’-pentamethoxyfavone | OMe | OH | OMe | OMe | OMe | None | OMe | OH | None | AO |
| 5,4’-Dihydroxy-3,6,7,8-tetramethoxyfavone | OMe | OH | OMe | OMe | OMe | None | None | OH | None | AO |
| 5,4’-Dihydroxy-3,6,7-trimethoxyfavone | OMe | OH | OMe | OMe | None | None | None | OH | None | AO |
| 5,6,3’,4’-Tetrahydroxy-3,7-dimethoxyflavone | OMe | OH | OH | OMe | None | None | OH | OH | None | AO |
| 5,7,4’-Trihydroxy-3-(hydromethylbutyl),3,6-dimethoxyfavone | OMe | OH | OMe | OH | None | None | Prenyl | OH | None | AO |
| 5,7-Dihydroxy-3’-(4’’-acetoxy-3;-methylbutenyl)  -3,6,4’-trimethoxylflavone | OMe | OH | OMe | OH | None | None | Prenyl | OMe | None | AO |
| 5,7-Dihydroxy-3’-(3-hydroxymethxylbutyl)  -3,6,4-trimethoxyflavone | OMe | OH | OMe | OH | None | None | Prenyl | OMe | None | AO |
| 5-Hydroxy-3,6,7,8,3’,4’-hexamethoxyflavone | OMe | OH | OMe | OMe | OMe | None | OMe | OMe | None | AO |
| 7,4’-Dihydroxy-flavanol-3-O-glucoside | Monosaccharide | None | None | OH | None | None | None | OH | None | AO |
| 5,7,4’’,5’’,3’’’,4’’’-hexahydroxy-3’’-O-glucosyl-3’,7’-biflavone | Monosaccharide | OH | OH | Flavonoid | None | None | OH | OH | None | AO |
| Falandioside B | Monosaccharide | OH | None | OH | None | None | None | OH | None | AO |
| Fisetin | OH | None | None | OH | None | None | OH | OH | None | AO, AI, AG |
| Galangin | OH | OH | None | OH | None | None | None | None | None | AO, AI |
| Gossypin | OH | OH | None | OH | Monosaccharide | None | OH | OH | None | AO, AI, AG |
| Icarrin | Monosaccharide | None | None | Monosaccharide | Prenyl | None | None | OMe | None | AO, AI, AG |
| Isorhamnetin | OH | OH | None | OH | None | None | OMe | OH | None | AO, AI |
| Isorhamnetin-3-O-rutinoside | Monosaccharide | OH | None | OH | None | None | OMe | OH | None | AO, AI |
| Kaempferol | OH | OH | None | OH | None | None | None | OH | None | AO, AI, AG |
| Kaempferol-3-methylether | OMe | OH | None | OH | None | None | None | OH | None | AO |
| Kaempferol-3-O-[α-L-rhamnopyranosyl-(1-6)]  -[(β-D-glucopyranosyl-(1-2)]-β-D-glucopyranoside | Trisaccharide | OH | None | OH | None | None | None | OH | None | AO |
| Kaempferol-3-O-D-glucopyranosyl-(1→2)-L-  rhamnopyranosyl-(1→6)-L-rhamnopyranoside | Trisaccharide | OH | None | OH | None | None | None | OH | None | AO |
| Kaempferol-3-O-rhamnopyranosyl-(1-6)-D-  glucopyranosyl-(1-2)-B-D-glucopyranoside | Disaccharide | OH | None | OH | None | None | None | OH | None | AO |
| Kaempferol-3-O-α-L-rhamnopyranosyl-(1-6)-β-D-  glucopyranosyl-(1-2)-β-D-glucopyranoside | Disaccharide | OH | None | OH | None | None | None | OH | None | AO |
| Kaempferol 3-O-β-glucopyranosyl(1 → 2)-β-  galactopyranoside-7-O-α-rhamnopyranoside | Disaccharide | OH | None | OH | None | None | None | OH | None | AO |
| Kaemferol-3-O-α-L-rhamnopyranosyl-7-O-  [β-D-glucopyranosyl-(1-2)-O-L-rhamnoside | Monosaccharide | OH | None | Disaccharide | None | None | None | OH | None | AO |
| Kaempferol-3-O-glucose-7-0-rhamnoside | Monosaccharide | OH | None | Monosaccharide | None | None | None | OH | None | AO |
| Kaempfero-3-O-rhamnoside | Monosaccharide | OH | None | OH | None | None | None | OH | None | AO |
| Astragalin | Monosaccharide | OH | None | OH | None | None | None | OH | None | AO |
| Name | C3 | C5 | C6 | C7 | C8 | C2’ | C3’ | C4’ | C5’ | Medicinal Propertiess |
| Kamepferol-3-O-glucuronide | Monosaccharide | OH | None | OH | None | None | None | OH | None | AO, AI |
| Kaemferol-3-(6''-methylgucuronide) | Monosaccharide | OH | None | OH | None | None | None | OH | None | AO |
| Kaempferol 3-O-robinobioside | Disaccharide | OH | None | OH | None | None | None | OH | None | AO, AI |
| 6,7-Dimethylkaempferol | OH | OH | OMe | OMe | None | None | None | OH | None | AO |
| Linarin | OH | OH | None | Disaccharide | None | None | None | OMe | None | AO, AI, AG |
| Melanoxetin | OH | None | None | OH | OH | None | OH | OH | None | AO |
| Morin | OH | OH | None | OH | None | OH | None | OH | None | AO, AI, AG |
| Myricetin | OH | OH | None | OH | None | None | OH | OH | OH | AO, AI, AG |
| Myricetin-3-O-glucopyranoside | Monosaccharide | OH | None | OH | None | None | OH | OH | OH | AO, AI, AG |
| Paucatalinone E | OH | OH | Prenyl | OH | None | None | OMe | OH | OMe | AO |
| Penduletin | OH | OMe | OMe | OMe | None | None | OMe | OH | OMe | AO, AI |
| Pectolinarin | OH | OH | OMe | Disaccharide | None | None | None | OH | None | AO, AI, AG |
| Quercetin | OH | OH | None | OH | None | None | OH | OH | None | AO, AI, AG |
| Quercetin-3-O-xyloside | Monosaccharide | OH | None | OH | None | None | OH | OH | None | AO, AI |
| Isoquercitin | Monosaccharide | OH | None | OH | None | None | OH | OH | None | AO, AI |
| Quercetin-3-O-arabinoside | Monosaccharide | OH | None | OH | None | None | OH | OH | None | AO |
| Quercimeritrin | OH | OH | None | Monosaccharide | None | None | OH | OH | None | AO, AI |
| Quercetin-3-O-D-glucopyranosyl-(1→2)-L-  rhamnopyranosyl-(1→6)-L-rhamnopyranoside | Trisaccharide | OH | None | OH | None | None | OH | OH | None | AO |
| Quercetrin | Monosaccharide | OH | None | OH | None | None | OH | OH | None | AO, AI, AG |
| Rutin | Disaccharide | OH | None | OH | None | None | OH | OH | None | AO, AI, AG |
| Quercetin-3-O-D-glucuronide | Monosaccharide | OH | None | OH | None | None | OH | OH | None | AO |
| Isoquercitrin | Monosaccharide | OH | None | OH | None | None | OH | OH | None | AO, AI |
| Quercetin-3-methoxy-4-O-glucoside | OMe | OH | None | Monosaccharide | None | None | OH | Monosaccharide | None | AO, AI, AG |
| Azaleatin | OH | OMe | None | OH | None | None | OH | OH | None | AO, AI, AG |
| Quercetin 3,7-O-dirhamnoside | Monosaccharide | OH | None | Monosaccharide | None | None | OH | OH | None | AO, AI, AG |
| α-Rhamnoisorobin | OH | OH | None | Monosaccharide | None | None | None | OH | None | AO, AI, AG |
| Santin | OMe | OH | OMe | OH | None | None | None | OMe | None | AO |
| Transilitin | OMe | None | None | OH | OH | None | OH | OH | None | AO |
| Viscosine | OMe | OH | OMe | OH | None | None | None | OH | None | AO, AI, AG |

Table S12. The organic functional groups in isoflavones’ substitution sites

| Name | C3 | C5 | C6 | C7 | C8 | C2’ | C3’ | C4’ | C5’ | Medicinal Properties |
| --- | --- | --- | --- | --- | --- | --- | --- | --- | --- | --- |
| Biochanin-A-7-O-glucoside | None | OH | None | Monosaccharide | None | None | None | OMe | None | AO |
| Daidzein | None | None | None | OH | None | None | None | OH | None | AO, AI, AG |
| Formononetin | None | None | None | OH | None | None | None | OMe | None | AO, AI, AG |
| Genistein | None | OH | None | OH | None | None | None | OH | None | AO, AI, AG |
| Glabridin | None | None | None | Prenyl | Prenyl | OH | None | OH | None | AO, AI, AG |
| Glycitein | None | None | OMe | OH | None | None | None | OH | None | AO, AI, AG |
| Glyurallin B | None | OH | None | OH | Prenyl | None | Prenyl | OH | OH | AO, AI, AG |
| Orobol-7-O-glucoside | None | OH | None | Monosaccharide | None | None | OH | OH | None | AO, AI, AG |
| Scabdenone | None | OH | Prenyl | Prenyl | Prenyl | None | None | OH | None | AO, AI, AG |
| Echinoisoflavanone | OH | OH | None | OH | None | OMe | Prenyl | OMe | None | AO, AI |
| Echinoisophoranone | None | OH | None | OMe | None | OMe | None | OH | Prenyl | AO, AI |
| Isosophoranone | None | OH | Prenyl | OH | None | OMe | Prenyl | None | None | AO, AI |
| 5,7,4'-Trihydroxy-6,8-diprenylisoflavone | None | OH | Prenyl | OH | Prenyl | None | None | OH | None | AO, AI |
| Warangalone | None | OH | Prenyl | OH | Prenyl | None | None | OH | None | AO, AI |
| Gancaonin A | None | OH | Prenyl | OH | Prenyl | None | None | OH | None | AO, AI |
| Isoerysenegalensein E | None | OH | Prenyl | OMe | Prenyl | None | None | OMe | None | AO, AI |
| 7-Methoxyebenosin | None | None | Prenyl | OMe | Prenyl | None | None | OMe | None | AO, AI |
| Ficucaricone D | None | OMe | Prenyl | OMe | None | None | None | OMe | None | AO, AI |
| 4'-Hydroxy-5,7-dimethoxy-6(3-methyl-2-buteny)isoflavone | None | OMe | Prenyl | OMe | None | None | None | None | OH | AO, AI |
| Gancaonin N | None | OH | Prenyl | OH | None | OH | None | OMe | None | AO, AI |
| Isopiscerythrone | None | OH | Prenyl | OH | None | OH | None | OH | OMe | AO, AI |
| Viridiflflorin | None | OH | Prenyl | OH | None | OMe | None | OH | OMe | AO, AI |

Table S13. The organic functional groups in flavones’ substitution sites

| Name | C3 | C5 | C6 | C7 | C8 | C2’ | C3’ | C4’ | C5’ | C6’ | Medicinal Properties |
| --- | --- | --- | --- | --- | --- | --- | --- | --- | --- | --- | --- |
| Acacetin | None | OH | None | OH | None | None | None | OMe | None | None | AO, AI, AG |
| Apigenin | None | OH | None | OH | None | None | None | OH | None | None | AO, AI, AG |
| Apigenin-7-O-β-D-(6’’-ρ-coumaroly)glucopyranoside | None | OH | None | Monosaccharide | None | None | None | OH | None | None | AO, AI |
| Apigenin-7-glucoside | None | OH | None | Monosaccharide | None | None | None | OH | None | None | AO, AI |
| Apigenin-7-glucuronide | None | OH | None | Monosaccharide | None | None | None | OH | None | None | AO, AI |
| Vitexin | None | OH | None | OH | Monosaccharide | None | None | OH | None | None | AO, AI, AG |
| 3-C-glucopyranosylapigenin | Monosaccharide | OH | None | OH | None | None | None | OMe | None | None | AO |
| Vitexin-2-O-xyloside | None | OH | None | OH | disaccharide | None | None | OH | None | None | AO, AI |
| Vitexin-2''-O-rhamnoside | None | OH | None | OH | disaccharide | None | None | OH | None | None | AO |
| Amentoflavone | None | OH | None | OH | None | None | None | OH | Flavonoid | None | AO, AI, AG |
| Apiin | None | OH | None | Disaccharide | None | None | None | OH | None | None | AO, AI |
| Artogomezianone | Prenyl | OH | Prenyl | OMe | None | OH | None | OH | None | None | AO |
| Atalantoflavone | None | OH | None | Prenyl | Prenyl | None | None | OH | None | None | AO, AI |
| Baicalein | None | OH | OH | OH | None | None | None | None | None | None | AO, AI, AG |
| Oroxin B | None | OH | OH | Disaccharide | None | None | None | None | None | None | AO, AI |
| Balcalein-7-O-glucoside | None | OH | OH | Monosaccharide | None | None | None | None | None | None | AO |
| Baicalin | None | OH | OH | Monosaccharide | None | None | None | None | None | None | AO, AI, AG |
| Bilobetin | None | OH | None | OH | None | None | Flavonoid | OMe | None | None | AO, AI |
| Carambolflavone | None | OH | Disaccharide | OH | None | None | None | OH | None | None | AO |
| Chrysin | None | OH | None | OH | None | None | None | None | None | None | AO, AI, AG |
| Chrysin-7-glucuronide | None | OH | None | Monosaccharide | None | None | None | None | None | None | AO |
| Chrysoeriol | None | OH | None | OH | None | None | OMe | OH | None | None | AO, AI |
| Cosmosiin | None | OH | None | Monosaccharide | None | None | None | OH | None | None | AO |
| Diosmetin | None | OH | None | OH | None | None | OH | OMe | None | None | AO, AI, AG |
| Diosmetin 7-O-glucopyranoside | None | OH | None | Monosaccharide | None | None | OH | OMe | None | None | AO |
| Cirsillineol | None | OH | OMe | OMe | None | None | OMe | OH | None | None | AO |
| 5,6,4'-Trihydroxy-7,3'-dimethoxyflavone | None | OH | OH | OMe | None | None | OMe | OH | None | None | AO, AI |
| Isoscoparin 2''-O-(6'''-(E)-feruloyl)-glucopyranoside | None | OH | Disaccharide | OH | None | None | OMe | OH | None | None | AO, AI |
| Isoscoparin 2''-O-(6''''-(E)-ρ-coumaroyl)-glucopyranoside | None | OH | Disaccharide | OH | None | None | OMe | OH | None | None | AO, AI |
| Isocycloartobiloxanthone | Prenyl | OH | Prenyl | Prenyl | None | OH | None | OH | Prenyl | Prenyl | AO, AI |
| Isoorientin | None | OH | Monosaccharide | OH | None | None ne | None | OH | OH | None | AO, AI |
| Isovitexin | None | OH | Monosaccharide | OH | None | None | None | OH | None | None | AO, AI |
| Isovitexin-2''-O-α-L-rhamnopyranoside | None | OH | Disaccharide | OH | None | None | None | OH | None | None | AO |
| Isovitexin 2''-O-(6'''-(E)-feruloyl)-glucopyranoside | None | OH | Disaccharide | OH | None | None | None | OH | None | None | AO, AI |
| GB-1a | None | OH | None | OH | Flavonoid | None | None | OH | None | None | AO, AI, AG |
| Name | C3 | C5 | C6 | C7 | C8 | C2’ | C3’ | C4’ | C5’ | C6’ | Medicinal properities |
| GB-2a | None | OH | None | OH | Flavonoid | None | None | OH | OH | None | AO, AI, AG |
| Ginkgetin | None | OH | None | OMe | None | None | Flavonoid | OMe | None | None | AO, AI, AG |
| Hispidulin | None | OH | OMe | OH | None | None | None | OH | None | None | AO, AI |
| Homoplantaginin | None | OH | OMe | Monosaccharide | None | None | None | OH | None | None | AO, AI |
| Hyperoside | Monosaccharide | OH | None | OH | None | None | OH | OH | None | None | AO, AI |
| Luteolin | None | OH | None | OH | None | None | OH | OH | None | None | AO, AI, AG |
| Luteolin 6-C-glucoside | None | OH | None | OH | None | None | OH | OH | None | None | AO, AI |
| Luteoloside | None | OH | None | Monosaccharide | None | None | None | OH | OH | None | AO, AI |
| 6-OH-luteolin-7-O-glucoside | None | OH | OH | Monosaccharide | None | None | OH | OH | None | None | AO |
| Luteolin-7-O-glucoside | None | OH | None | Monosaccharide | None | None | OH | OH | None | None | AO, AI, AG |
| Luteolin-7-O-glucopyranoside | None | OH | None | Monosaccharide | None | None | OH | OH | None | None | AO |
| Luteolin-7-O-neohesperidoside | None | OH | None | Disaccharide | None | None | OH | OH | None | None | AO, AI |
| Luteolin3'-O-glucoside | None | OH | None | OH | None | None | Monosaccharide | OH | None | None | AO, AI, AG |
| Luteolin 4'-O-neohesperidoside | None | OH | None | OH | None | None | OH | Disaccharide | None | None | AO, AI, AG |
| Morusin | Prenyl | OH | None | Prenyl | Prenyl | OH | None | OH | None | None | AO, AI |
| Nobiletin | None | OMe | OMe | OMe | OMe | None | OMe | OMe | None | None | AO, AI |
| Orientin | None | OH | None | OH | Monosaccharide | None | OH | OH | None | None | AO, AI, AG |
| Oroxylin A | None | OH | OMe | OH | None | None | None | None | None | None | AO, AI |
| Paucatalinone C | None | OH | Prenyl | OH | None | None | OMe | OH | OH | None | AO |
| Paucatalinone D | None | OH | Prenyl | OH | OH | None | OMe | OH | OH | None | AO, AI |
| Rhamnetin | None | OH | None | OMe | None | None | OH | OH | None | None | AO, AI |
| Rhoifolin | None | OH | None | Disaccharide | None | None | None | OH | None | None | AO, AI |
| Robustaflavone | None | OH | None | OH | None | None | None | Flavonoid | OH | None | AO, AI |
| Scutellarein | None | OH | OH | OH | None | None | None | OH | None | None | AO, AI |
| Scutellarin | None | OH | OH | Monosaccharide | None | None | None | OH | None | None | AO, AI, AG |
| Scutellarein-7-O-glucoside | None | OH | OH | Monosaccharide | None | None | None | OH | None | None | AO, AI |
| Scutellarein-7-O-gentiobioside | None | OH | OH | Disaccharide | None | None | None | OH | None | None | AO |
| Schaftoside | None | OH | Monosaccharide | OH | Monosaccharide | None | None | OH | None | None | AO, AI |
| Tangeretin | None | OMe | OMe | OMe | OMe | None | None | OH | None | None | AO, AI |
| Tricin | None | OH | None | OH | None | None | OMe | OH | OMe | None | AO, AI |
| Tricin-7-O-glucopyranoisde | None | OH | None | Monosaccharide | None | None | OMe | OH | OMe | None | AO, AI |
| Tricin-5-O-glucopyranoside | None | Monosaccharide | None | OH | None | None | OMe | OH | OMe | None | AO, AI |
| Tricin-7-rutinoside | None | OH | None | Monosaccharide | None | None | OMe | OH | OMe | None | AO, AI |
| Wogonin | None | OH | None | OH | OMe | None | None | None | None | None | AO, AI, AG |

Table S14. The organic functional groups in chalcones’ substitution sites

| Name | C2 | C3 | C4 | C5 | C2’ | C3’ | C4’ | C5’ | C6’ | Medicinal Properties |
| --- | --- | --- | --- | --- | --- | --- | --- | --- | --- | --- |
| Carambolasides A | None | None | OH | None | OH | Monosaccharide | OH | None | OH | AO, |
| Carambolasides C | None | None | OH | None | OH | Disaccharide | OH | None | OH | AO |
| Cardamomin | None | None | None | None | OH | None | OH | None | OMe | AO, AI |
| 2’,4’-dihydroxychalcone | None | None | None | None | OH | None | OH | None | None | AO, AI |
| Echinatin | OMe | None | None | None | None | None | OH | None | None | AO, AI |
| Hesperidin methyl chalcone | None | OH | OMe | None | OMe | None | Disaccharide | None | OH | AO, AI, AG |
| Kuraridin | OH | None | OH | None | OMe | None | OH | Prenyl | OH | AO, AI |
| Licochalcone A | OMe | None | OH | Prenyl | None | None | OH | None | None | AO, AI, AG |
| Licochalcone B | OMe | OH | OH | None | None | None | OH | None | None | AO, AI |
| 5-(1,1-Dimethylallyl)-3,4,4-trihydroxy-2-methoxychalcone | OMe | OH | OH | Prenyl | None | None | OH | None | None | AO, AI |
| Okanin | None | OH | OH | None | OH | OH | OH | None | None | AO, AI |
| Isoliquiritigenin | None | None | OH | None | OH | None | OH | None | None | AO, AI, AG |
| Sophoradochromene | None | Prenyl | Prenyl | Prenyl | OH | Prenyl | OH | None | None | AO, AI |
| Isobacachalcone | None | None | OH | None | OH | Prenyl | OH | None | None | AO, AI |
| Morachalcone A | OH | None | OH | None | OH | Prenyl | OH | None | None | AO, AI |
| Xanthohumol | None | None | OH | None | OH | Prenyl | OH | None | OMe | AO, AI, AG |
| Phloretin rutinoside | None | None | OH | None | Disaccharide | None | OH | None | OH | AO, AI |


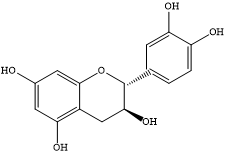

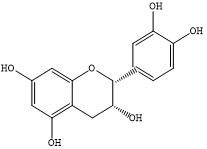

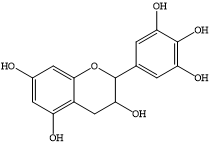

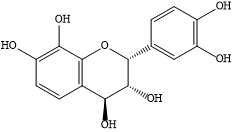


Catechin (AO, AI, AG) Epicatechin (AO, AI, AG) Gallocatechin (AO, AI, AG) Isomelacacidin (AO)


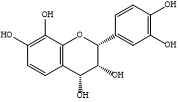

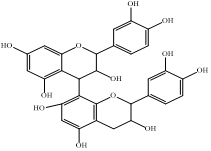

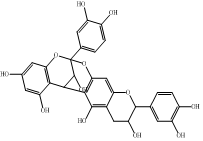

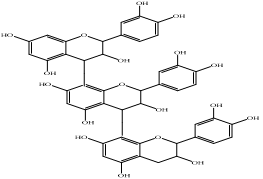


Melacacidin (AO) Proanthocyanidin B2 (AO) Proanthocyanidin A6 (AO) Procyanidin trimer C1 (AO)

Figure S1. Chemical structure of flavanols exhibiting antioxidant, anti-inflammatory, and analgesic abilities.

*Note: AO=Antioxidant ability; AI=Anti-inflammatory ability; AG=Analgesic ability*

Apigenindin (AO) Callistephin (AO, AI) Cyanindin (AO, AI, AG) Cyanidin-3-glucoside(AO, AI)

Cyanidin-3,5-diglucosides (AO, AI) Cyanidin 3-(6’’-malonylglucoside) (AI) Delphinidin (AO, AI, AG)

Delphinidin-3,5-diglucosides (AO) Malvin (AO) Malvidin-3-glucoside (oenin) (AO, AI)

Figure S2. Chemical structure of anthocyanins (and anthocyanidins) with antioxidant, anti-inflammatory, and analgesic abilities.

*Note: AO=Antioxidant ability; AI=Anti-inflammatory ability; AG=Analgesic ability*


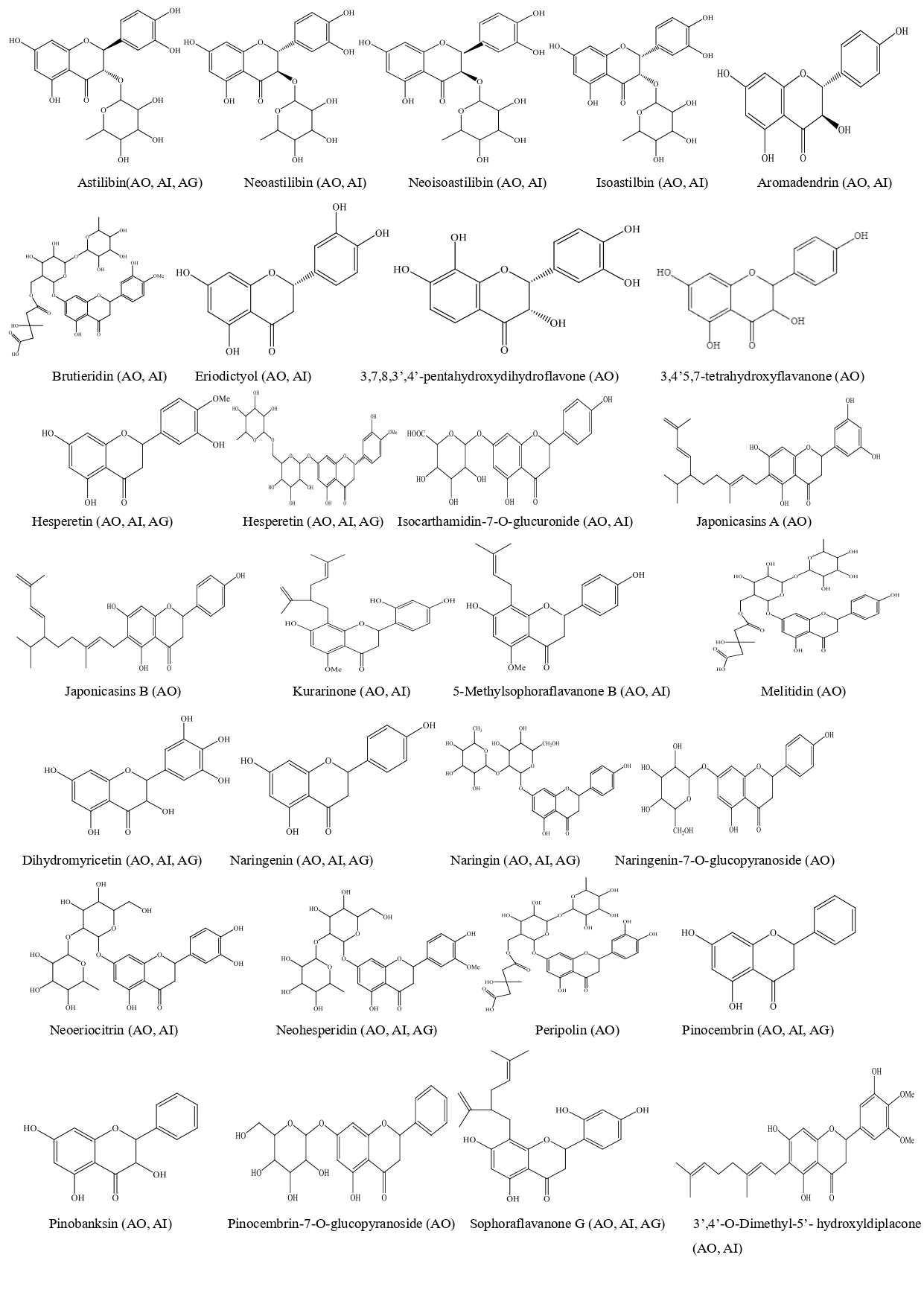


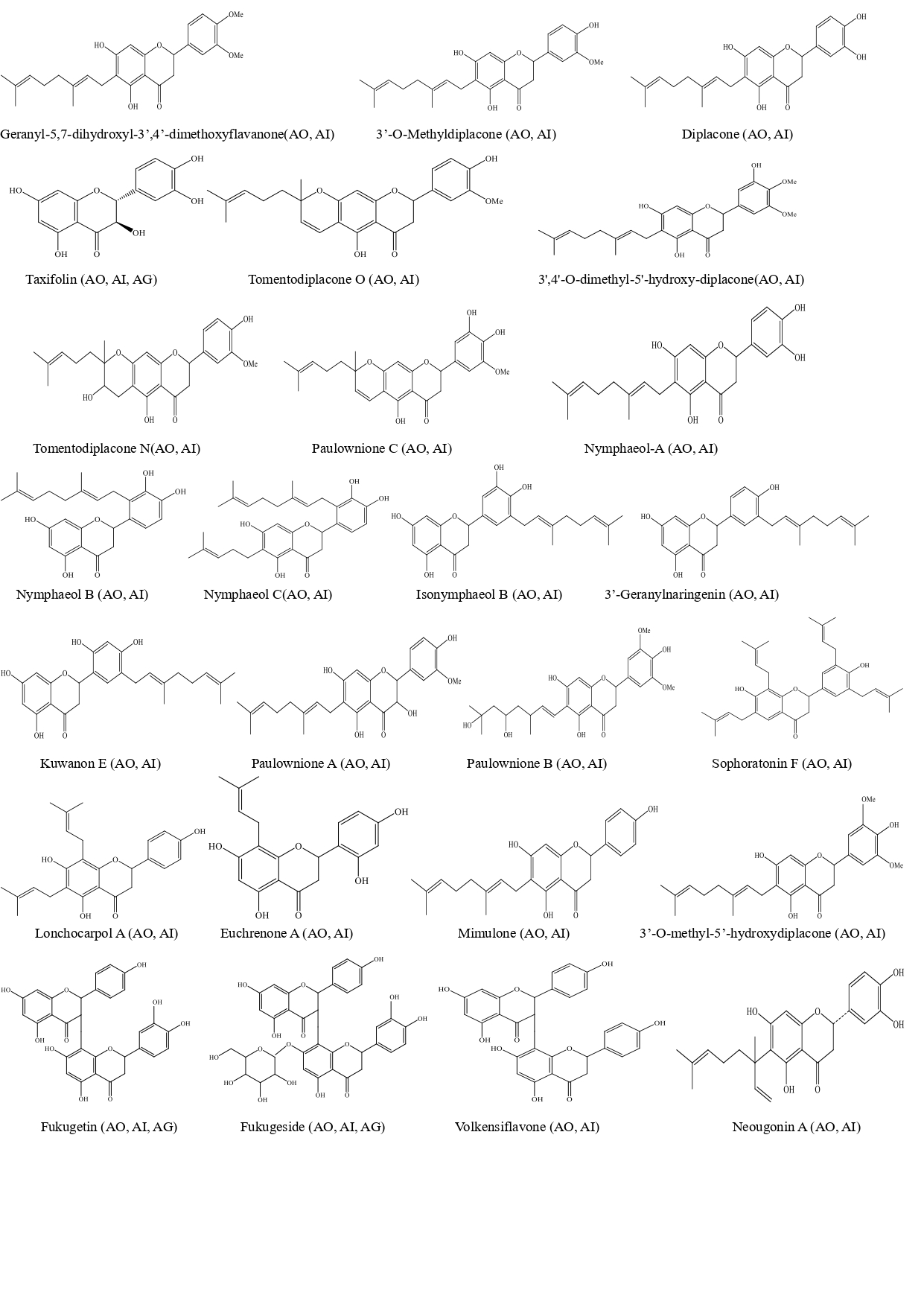


Figure S3. Chemical structure of flavanones with antioxidant, anti-inflammatory, and analgesic abilities.

*Note: AO=Antioxidant ability; AI=Anti-inflammatory ability; AG=Analgesic ability*


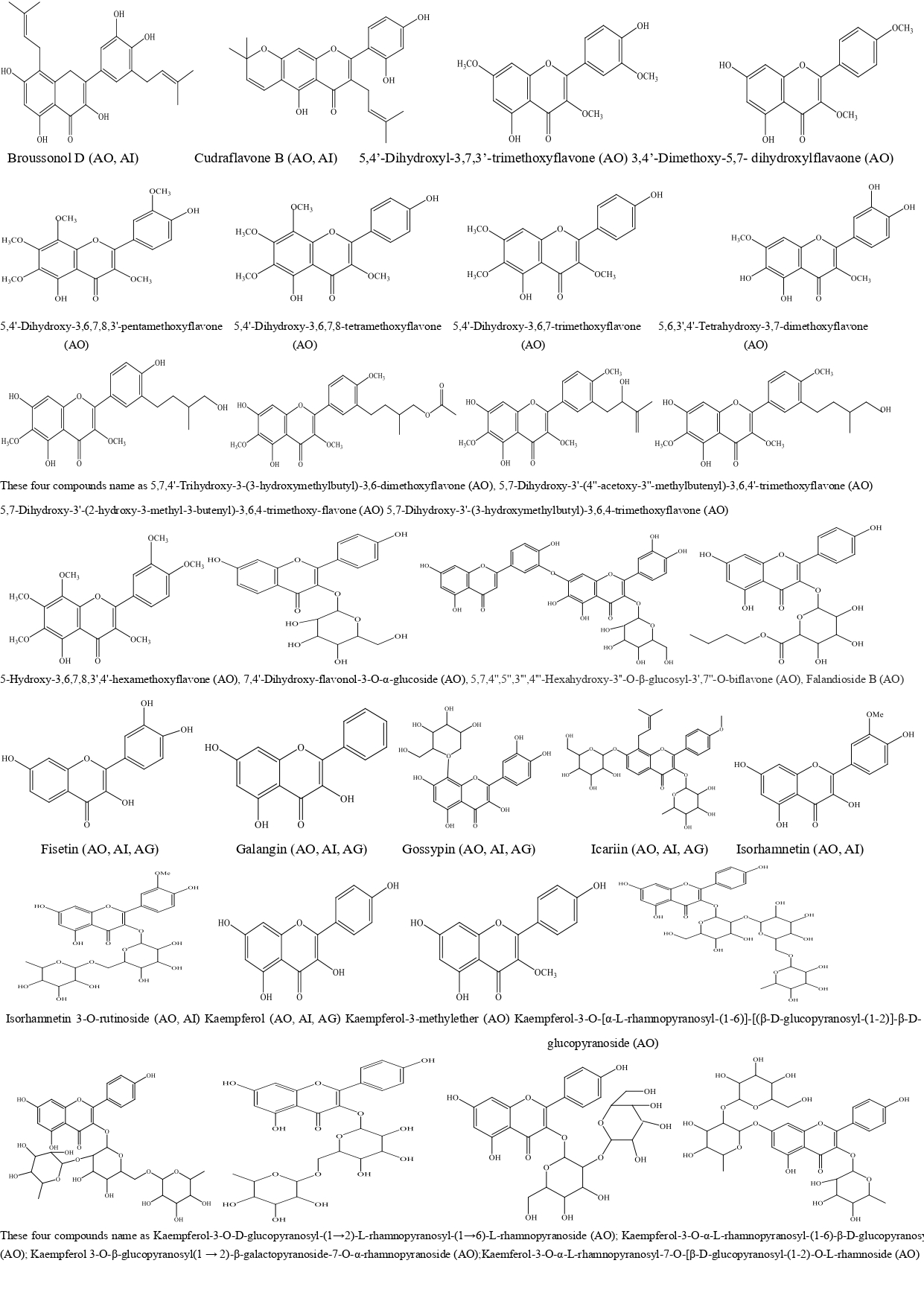


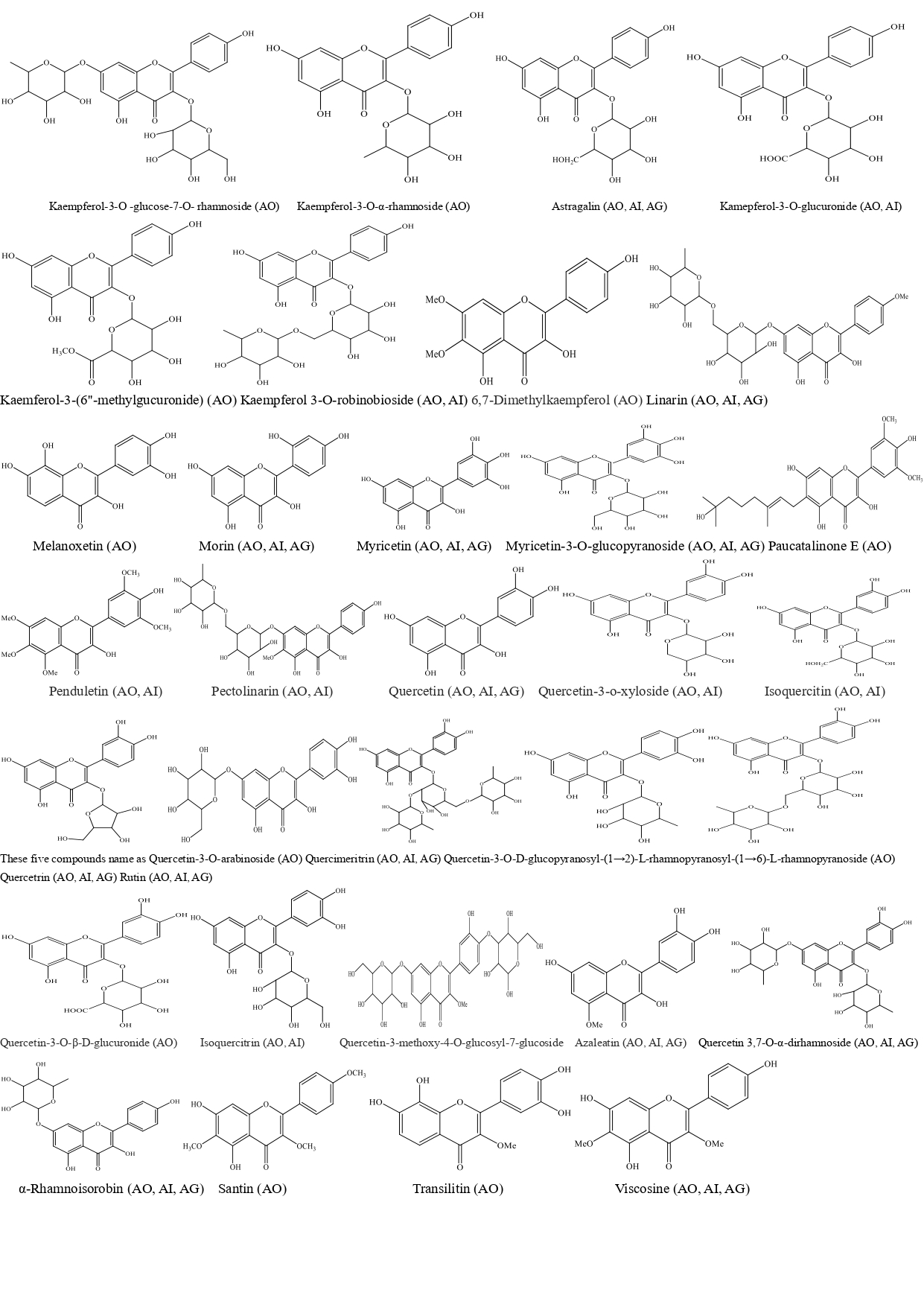


Figure S4. Chemical structure of flavonols with antioxidant, anti-inflammatory, and analgesic abilities.

*Note: AO=Antioxidant ability; AI=Anti-inflammatory ability; AG=Analgesic ability*


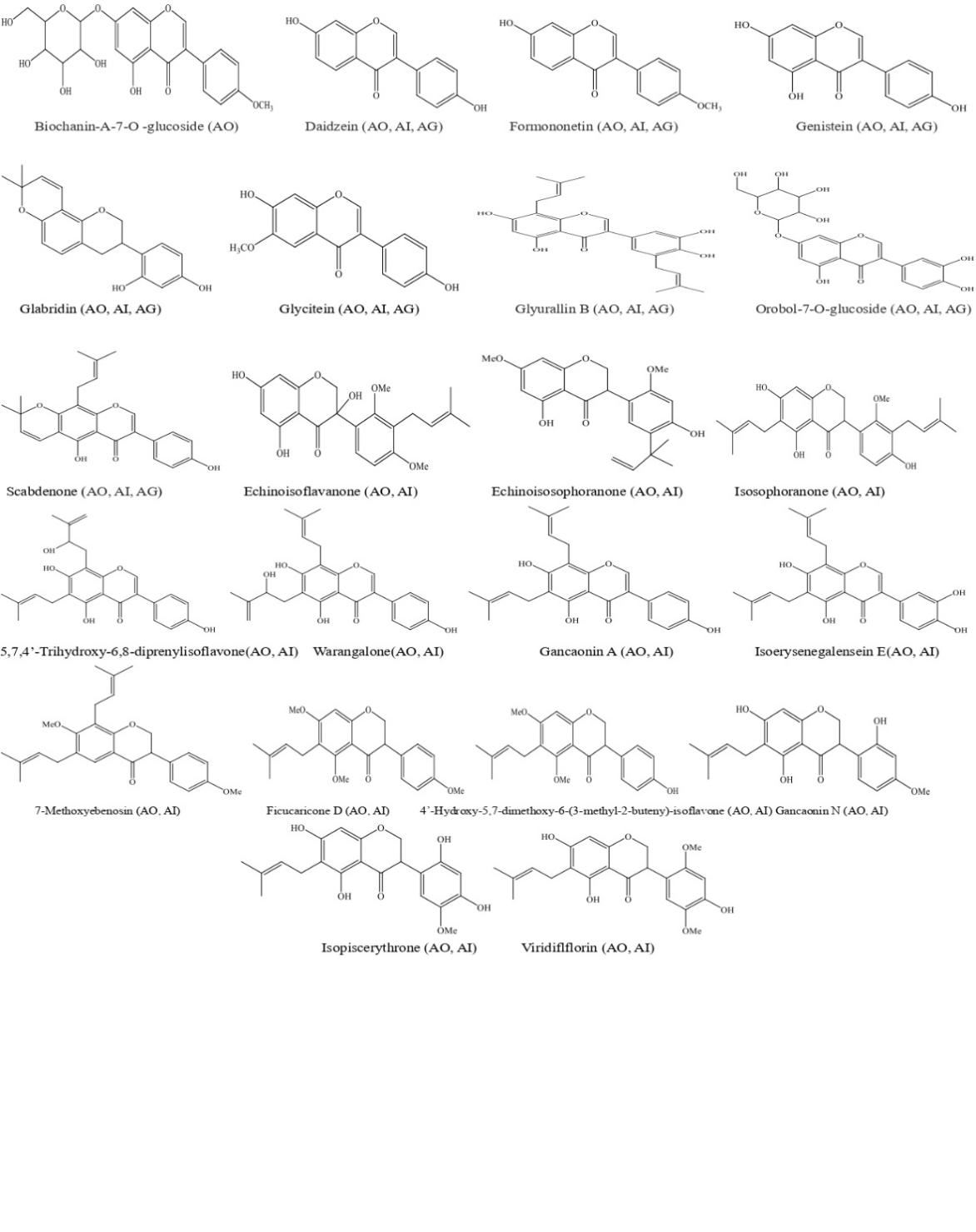


Figure S5. Chemical structure of isoflavones with antioxidant, anti-inflammatory, and analgesic abilities.

*Note: AO=Antioxidant ability; AI=Anti-inflammatory ability; AG=Analgesic ability*


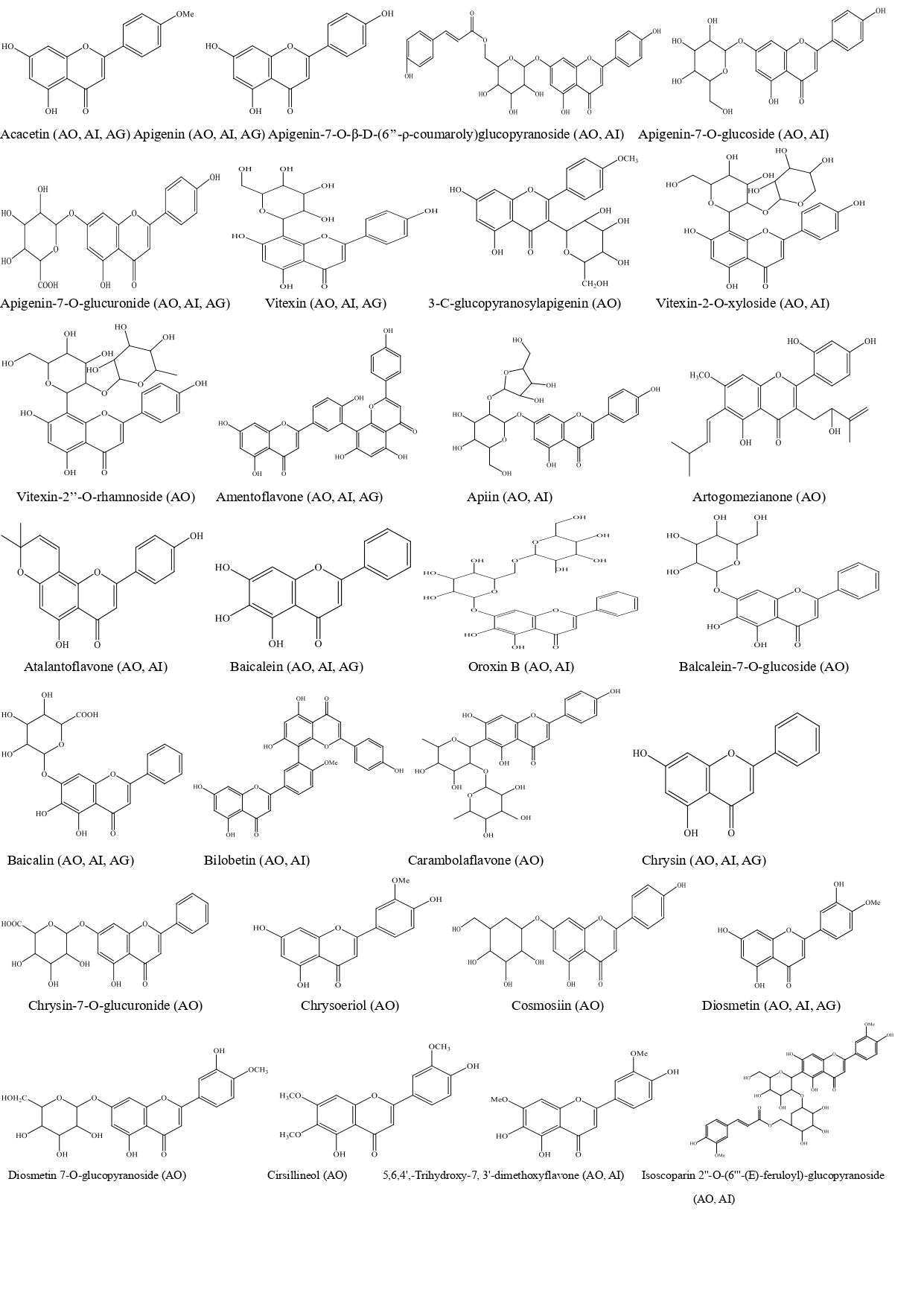


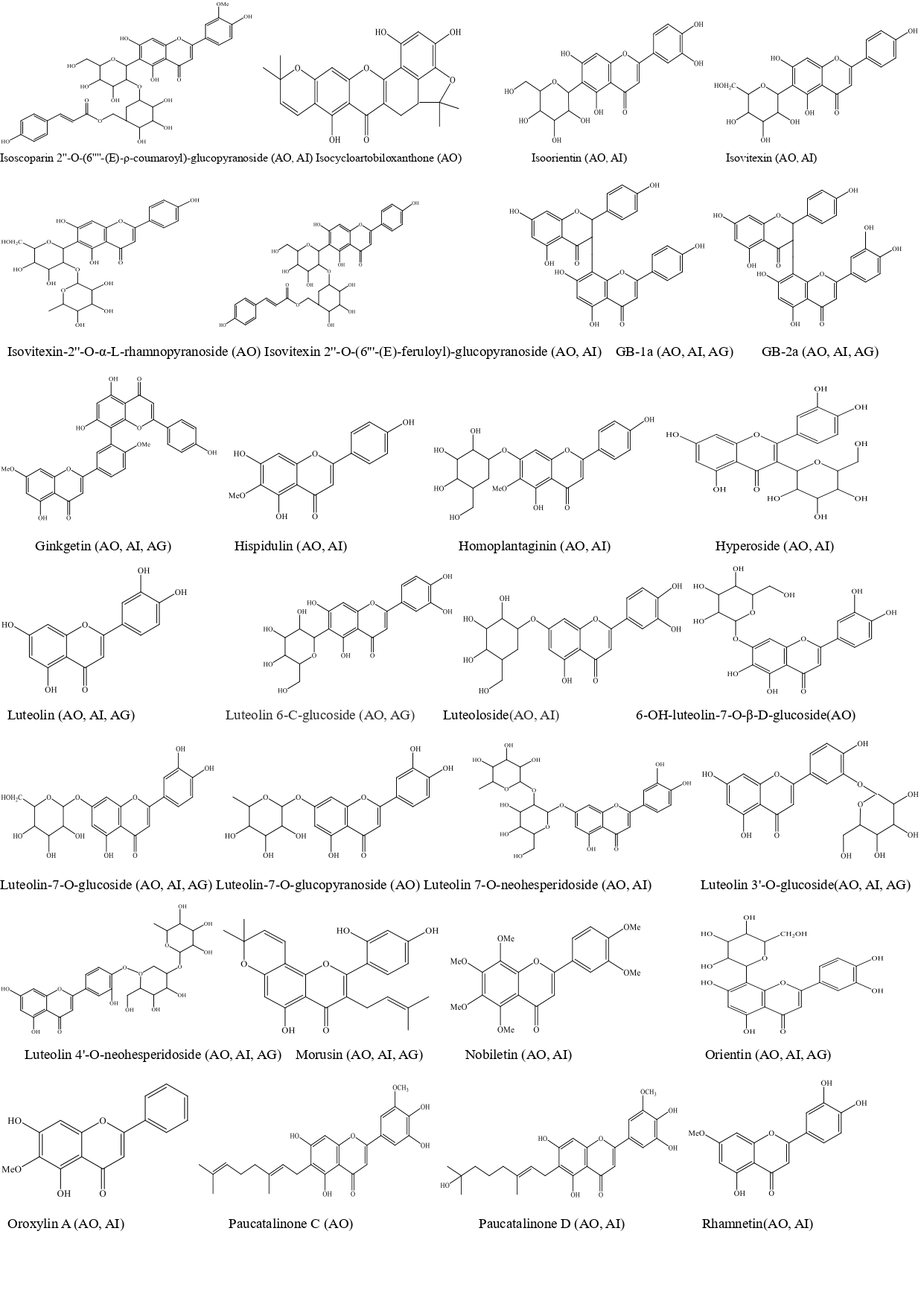


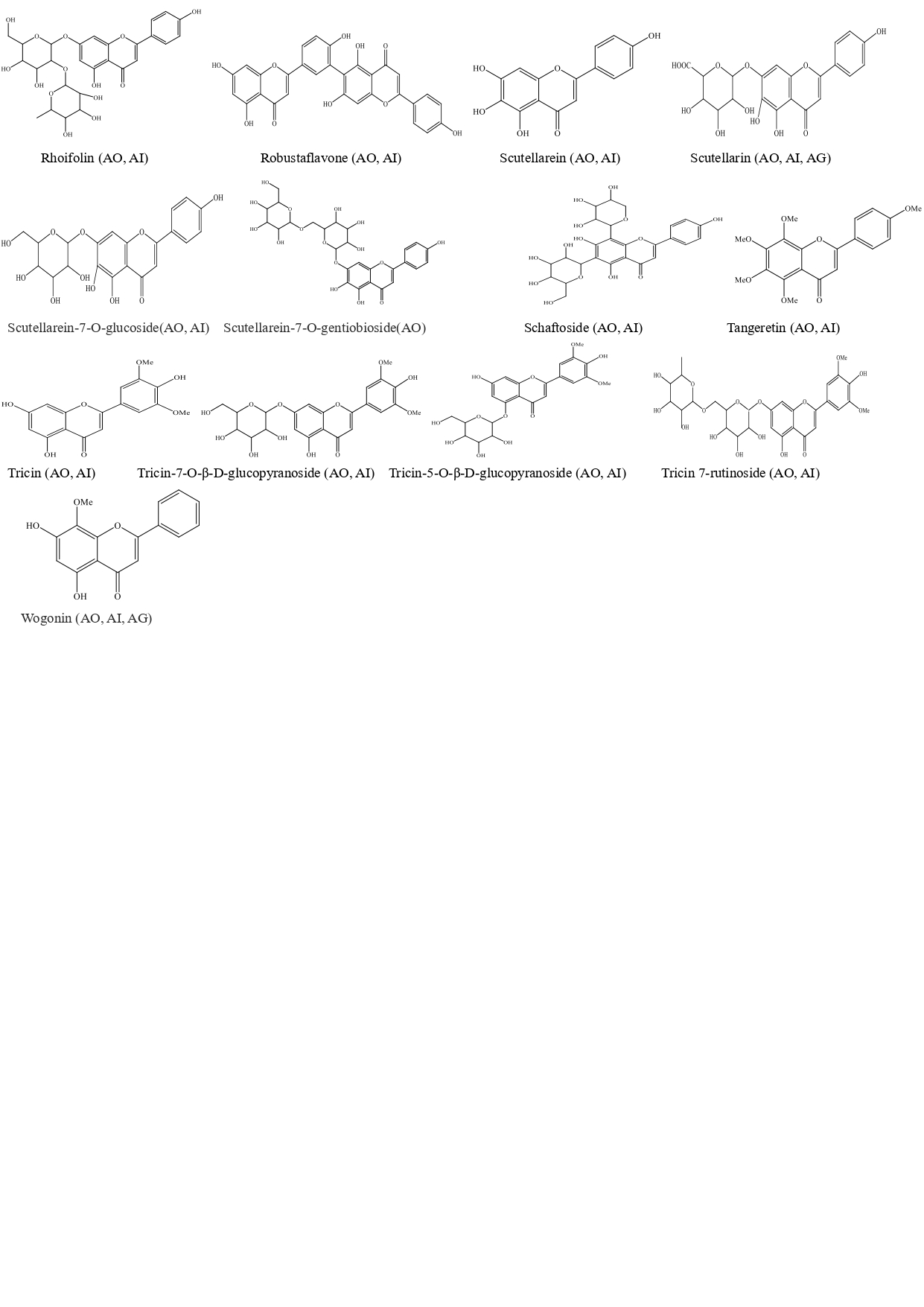


Figure S6. Chemical structure of flavones with antioxidant, anti-inflammatory, and analgesic abilities.

*Note: AO=Antioxidant ability; AI=Anti-inflammatory ability; AG=Analgesic ability*


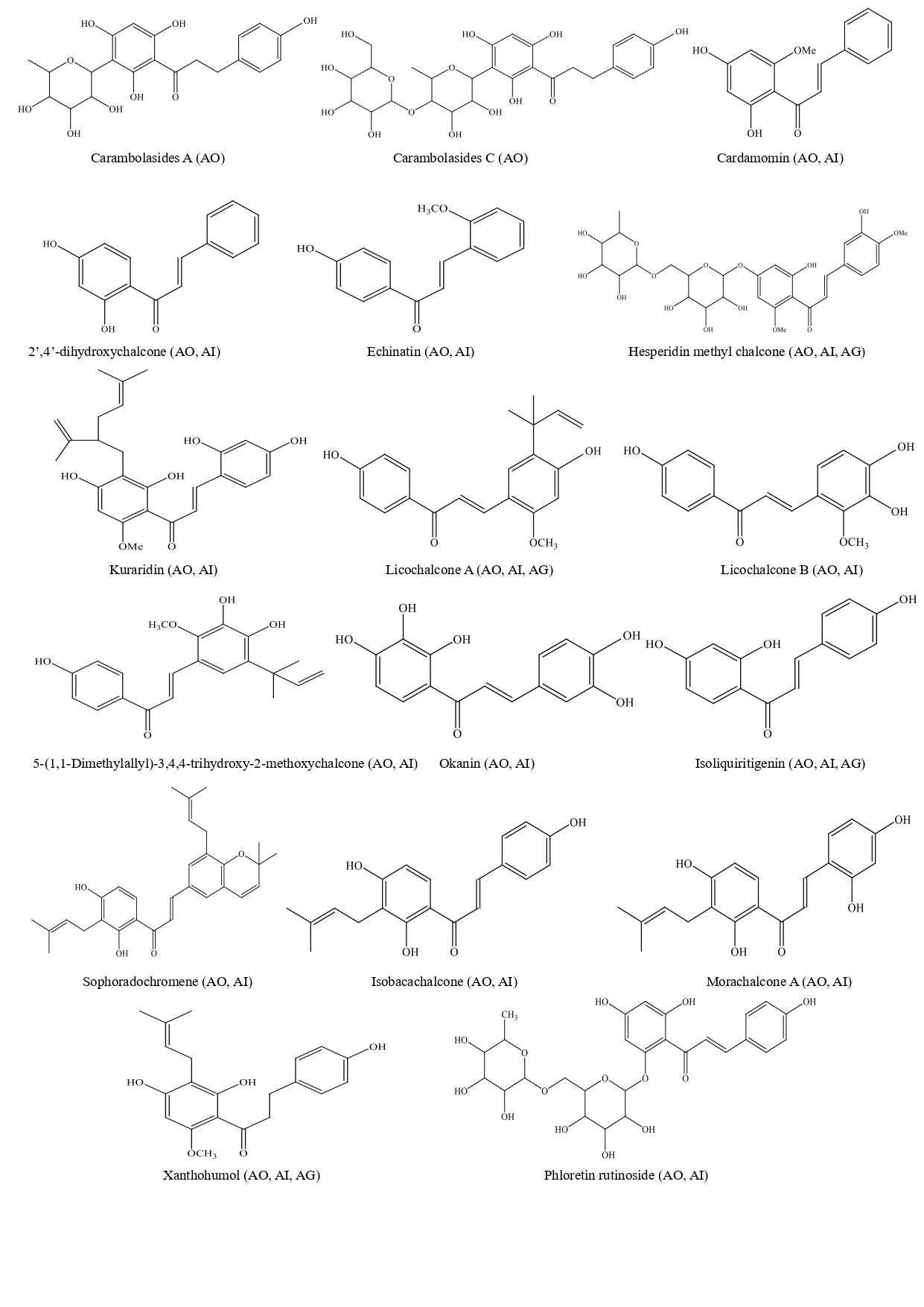


Figure S7. Chemical structure of chalcones with antioxidant, anti-inflammatory, and analgesic abilities.

*Note: AO=Antioxidant ability; AI=Anti-inflammatory ability; AG=Analgesic ability*


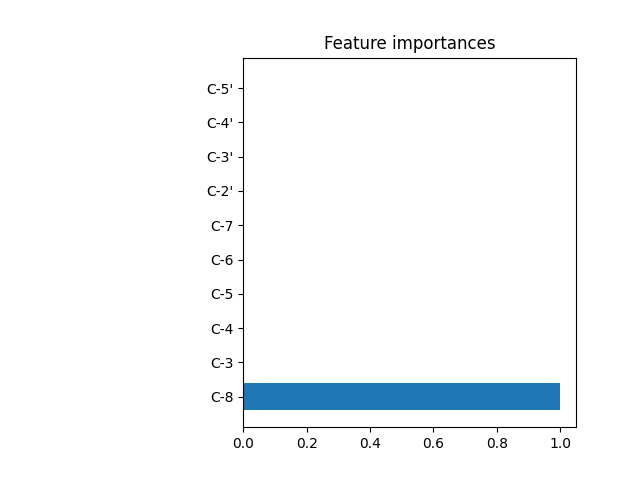

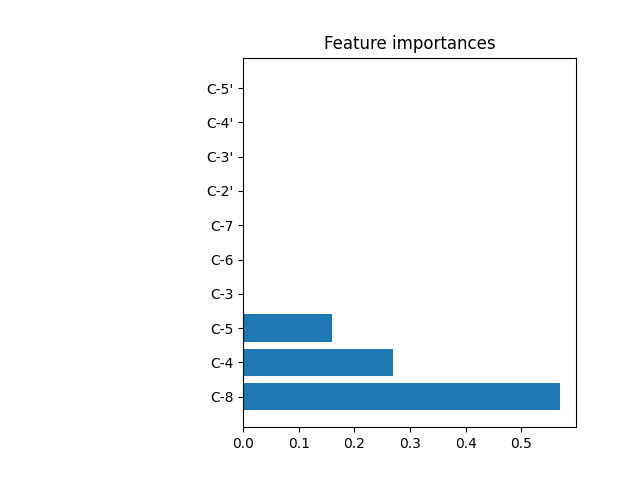

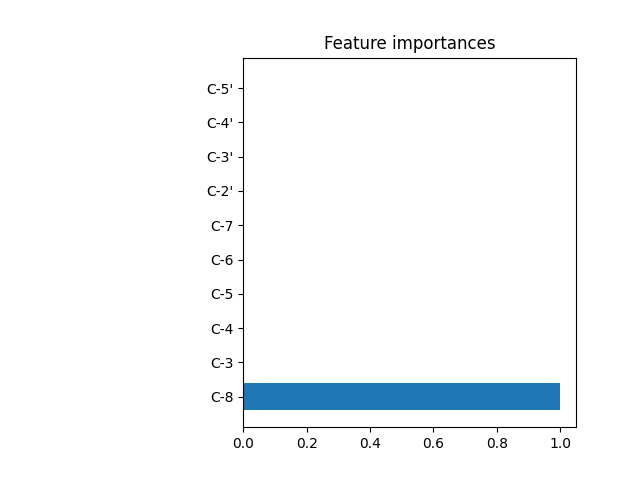

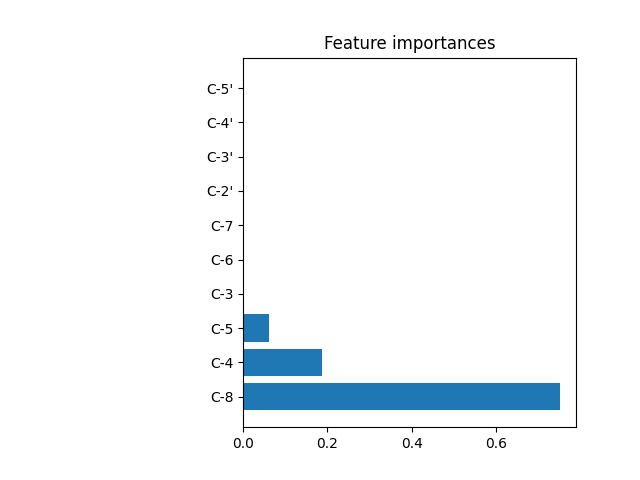


1. Gradient boost classifier (b) Extra tree classifier (c) Extreme gradient boost (d) Random Forest

Figure S8. The importance of organic functional group substitution sites on flavanols with antioxidant, anti-inflammatory and analgesic abilities.


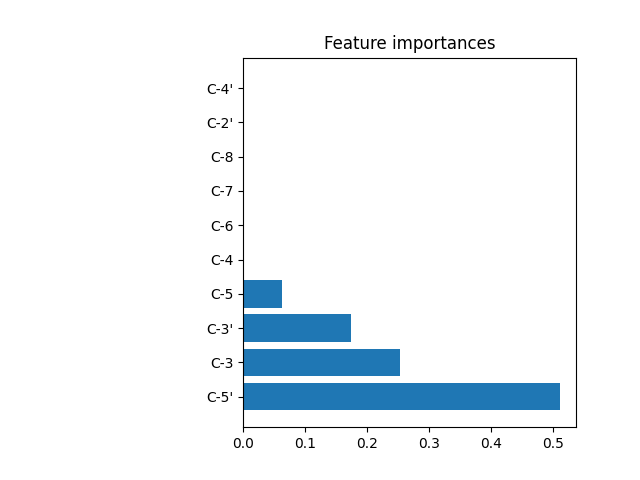

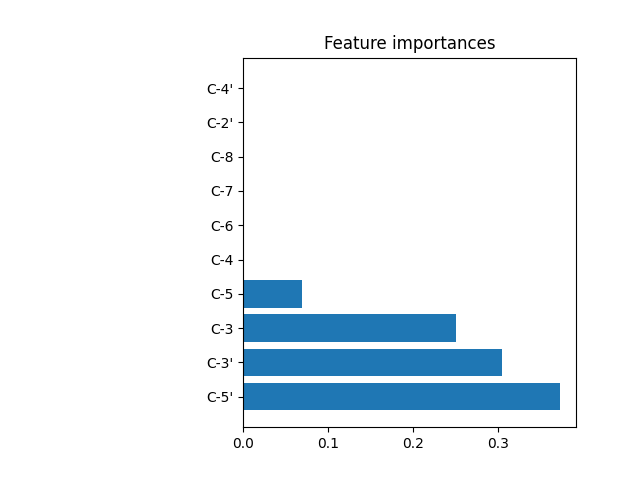

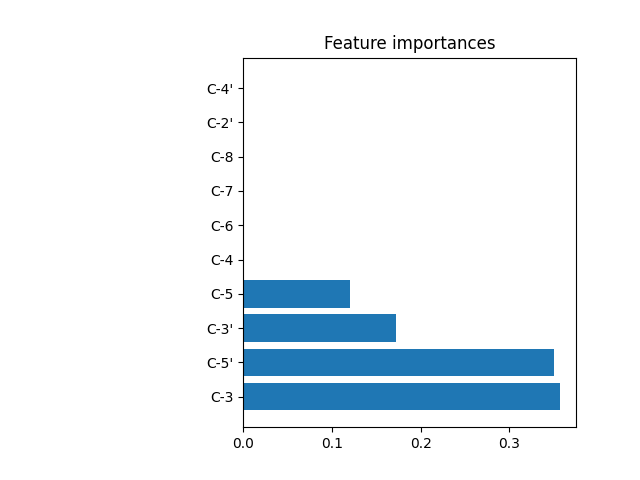

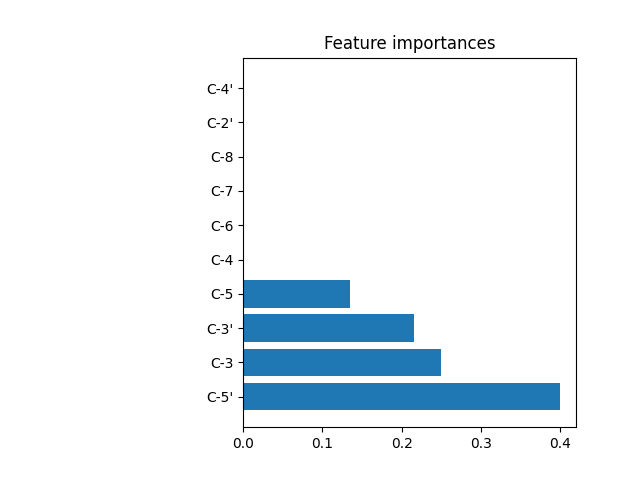


1. Gradient boost classifier (b) Extra tree classifier (c) Extreme gradient boost (d) Random Forest

Figure S9. The importance of organic functional group substitution sites on anthocyanins (and anthocyanidins) with antioxidant, anti-inflammatory and analgesic abilities.


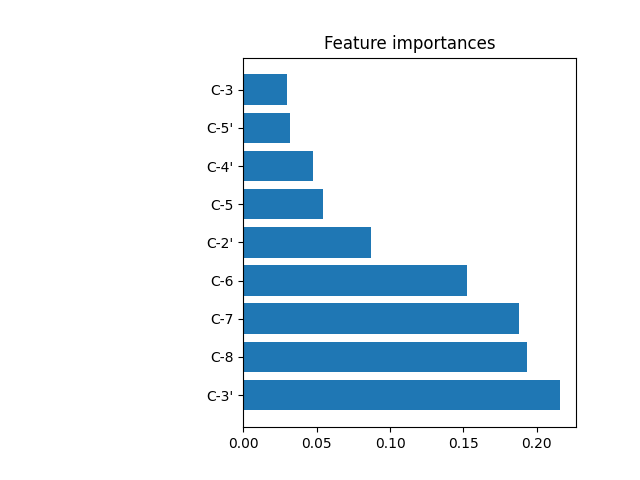

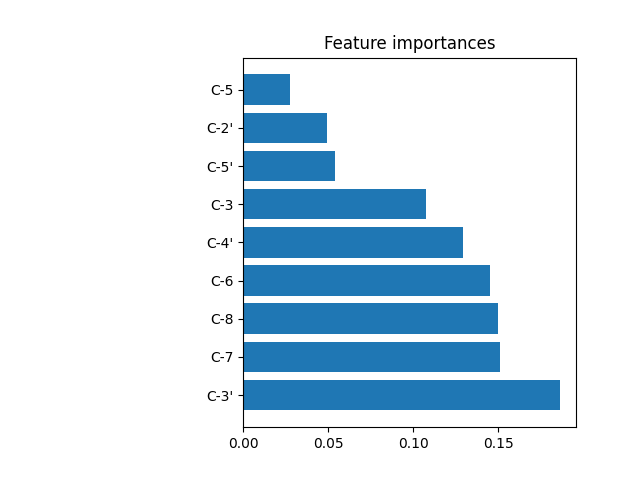

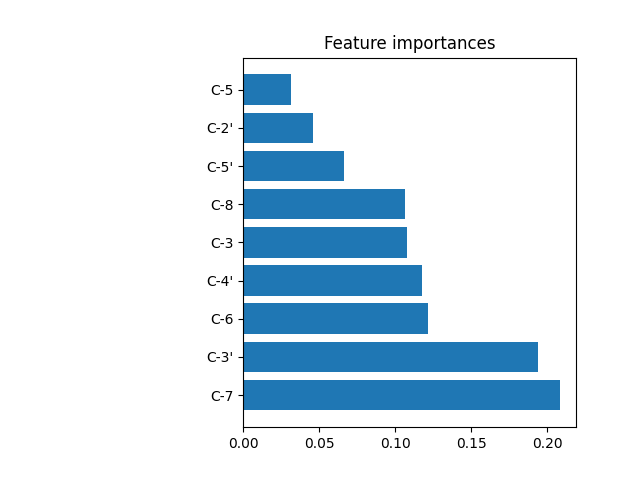

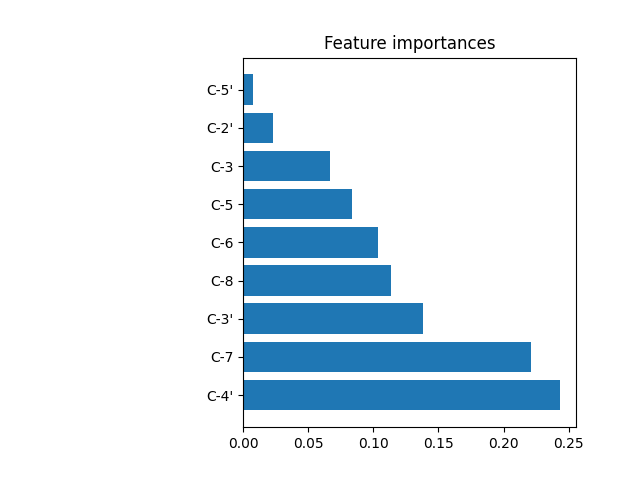


(a) Gradient boost classifier (b) Extra tree classifier (c) Extreme gradient boost (d) Random Forest

Figure S10. The importance of organic functional group substitution sites on flavanones with antioxidant, anti-inflammatory and analgesic abilities.


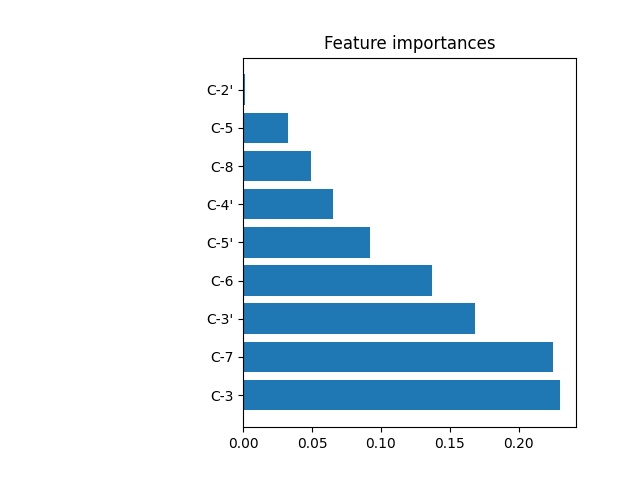

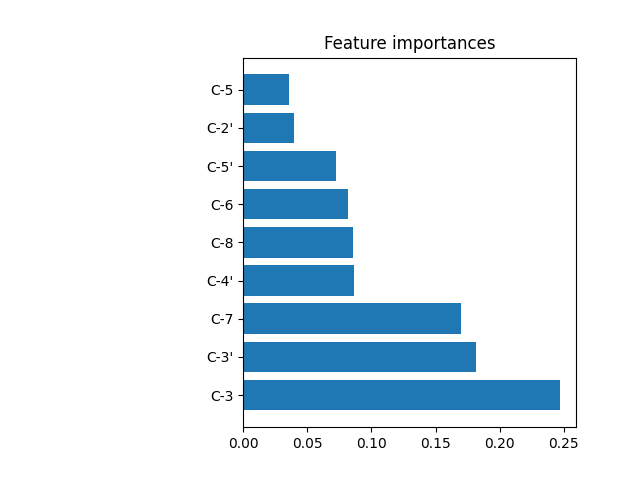

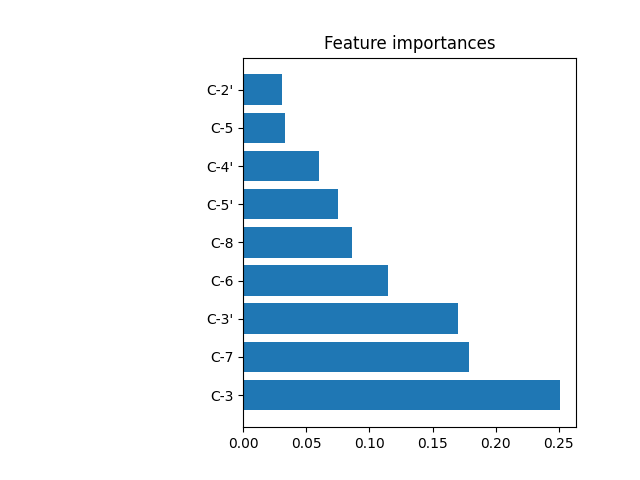

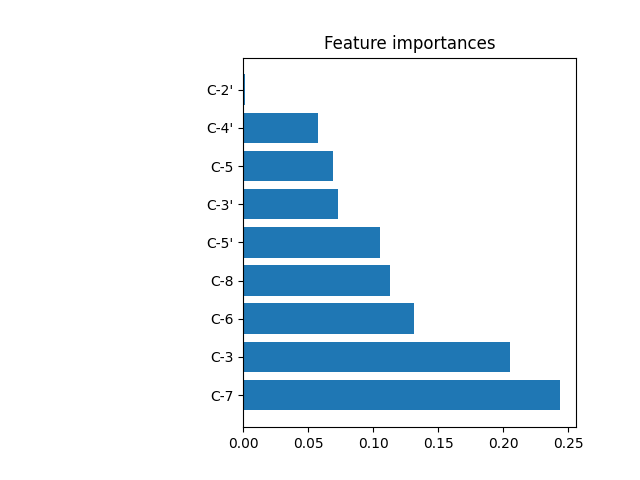


(a) Gradient boost classifier (b) Extra tree classifier (c) Extreme gradient boost (d) Random Forest

Figure S11. The importance of organic functional group substitution sites on flavonols with antioxidant, anti-inflammatory and analgesic abilities.


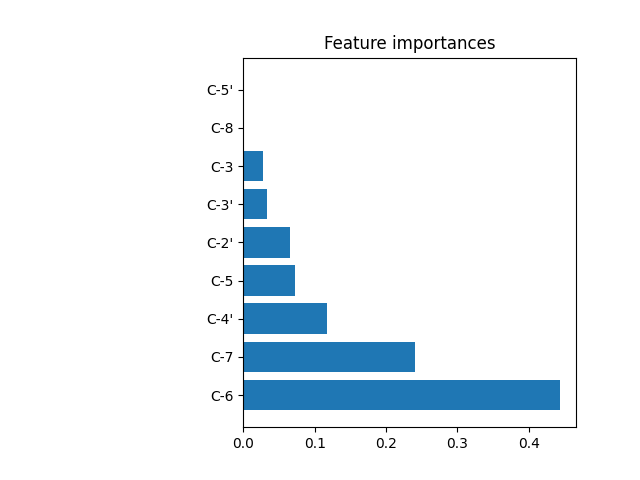

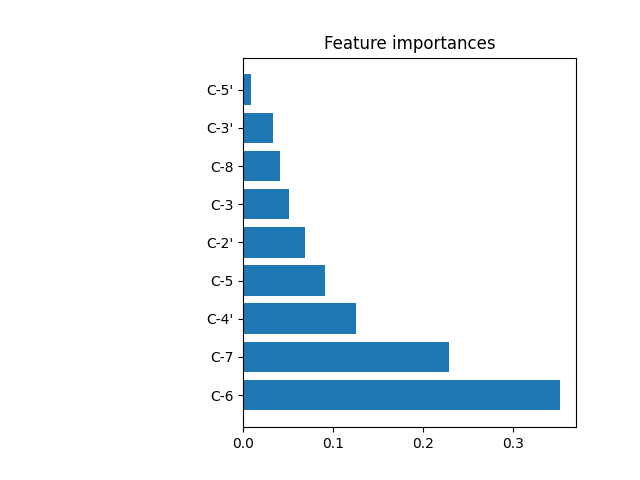

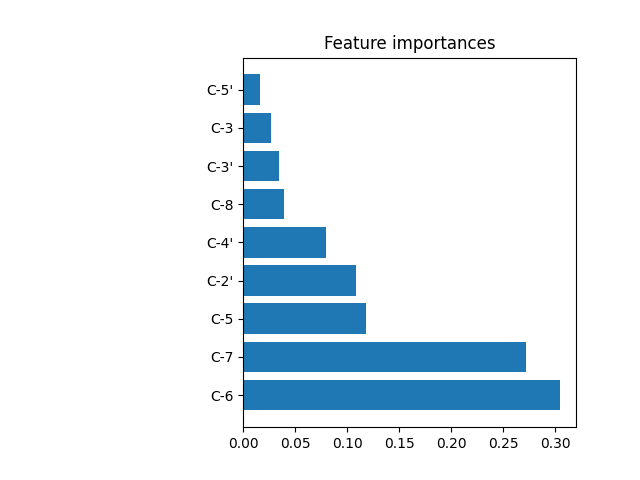

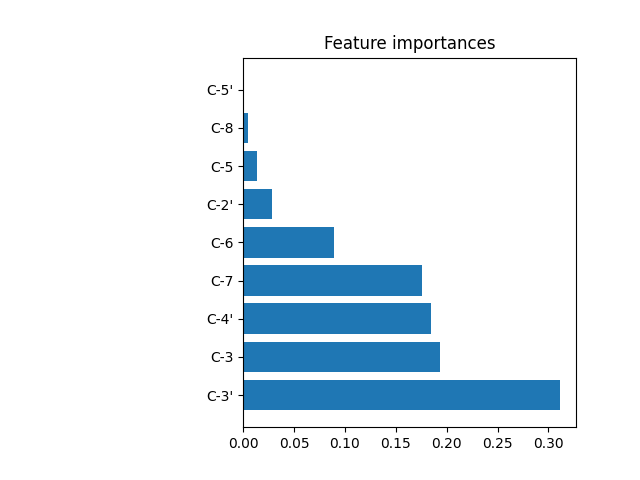


1. Gradient boost classifier (b) Extra tree classifier (c) Extreme gradient boost (d) Random Forest

Figure S12. The importance of organic functional group substitution sites on isoflavones with antioxidant, anti-inflammatory and analgesic abilities.


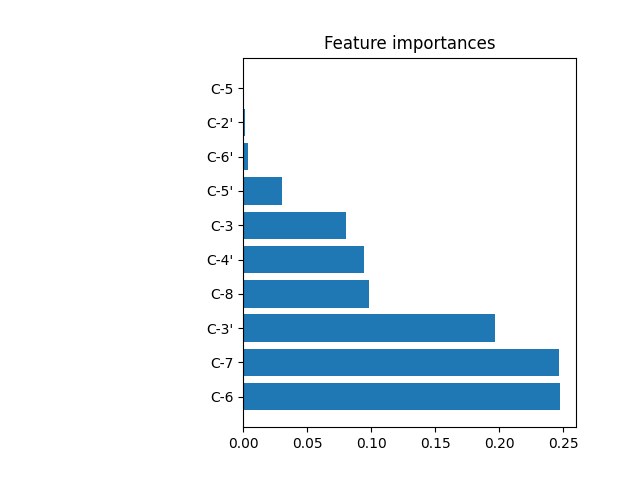

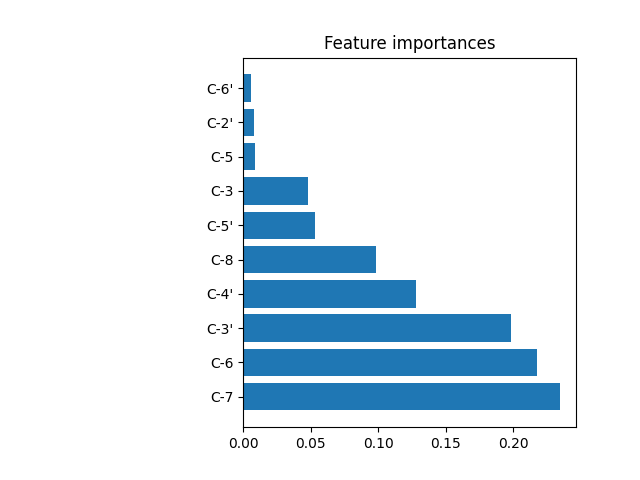

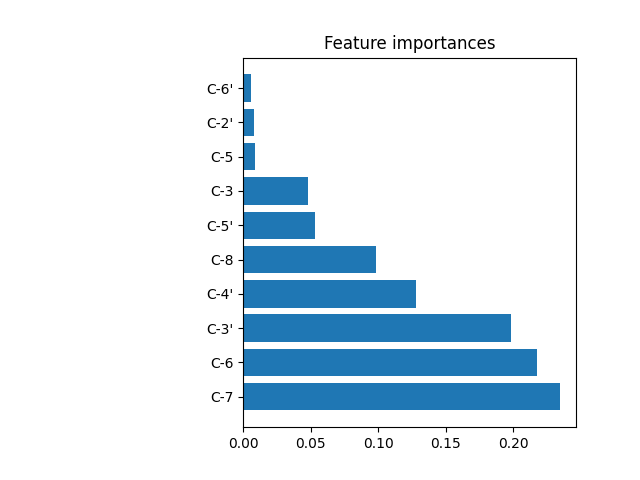

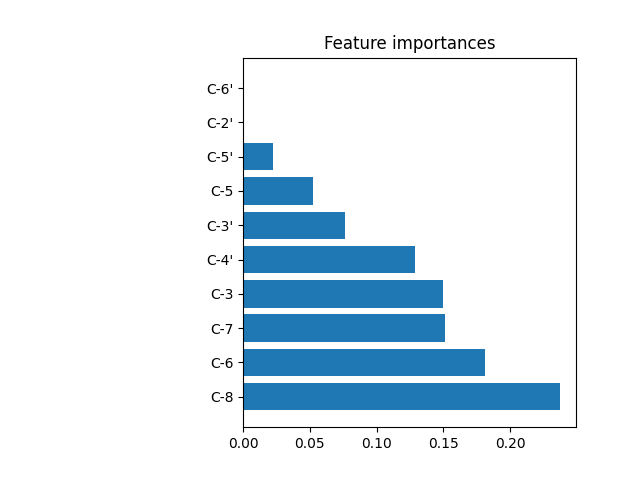


1. Gradient boost classifier (b) Extra tree classifier (c) Extreme gradient boost (d) Random Forest

Figure S13. The importance of organic functional group substitution sites on flavones with antioxidant, anti-inflammatory and analgesic abilities.


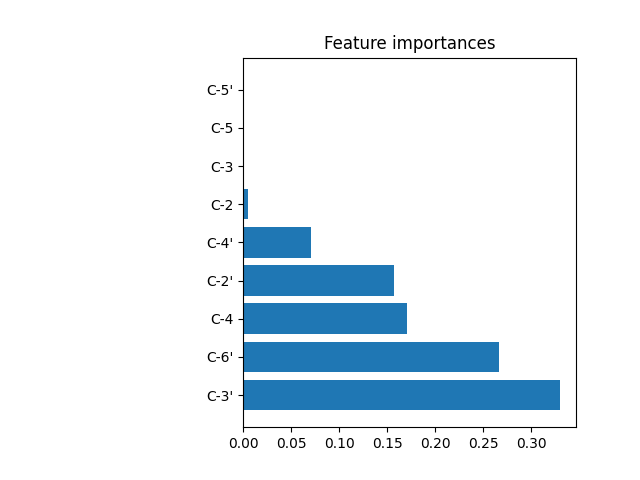

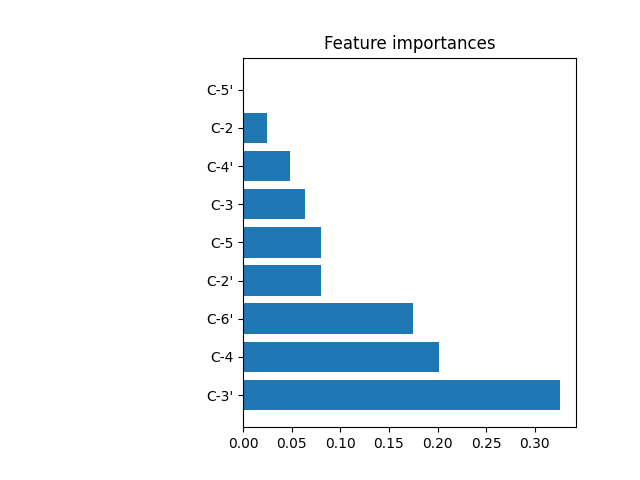

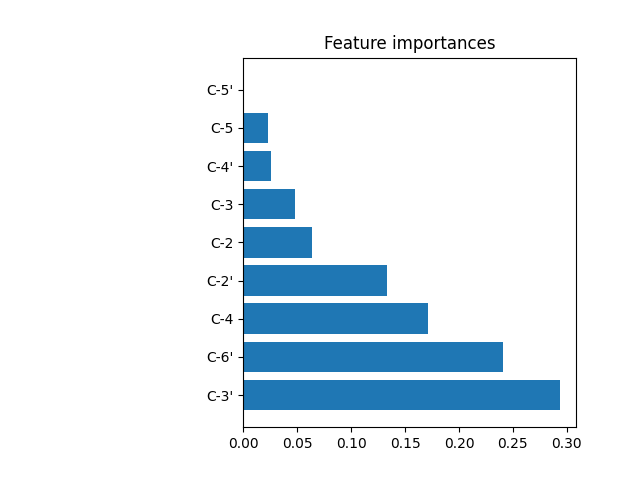

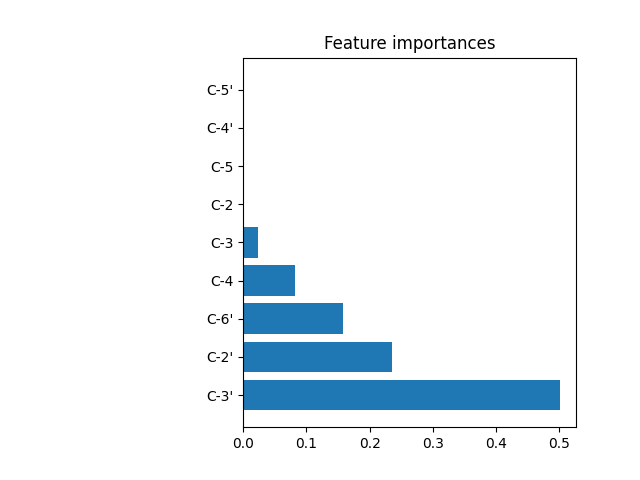


(a) Gradient boost classifier (b) Extra tree classifier (c) Extreme gradient boost (d) Random Forest

Figure S14. The importance of organic functional group substitution sites on chalcones with antioxidant, anti-inflammatory and analgesic abilities.

**References**

Abdallah, H. M., Almowallad, F. M., Esmat, A., Shehata, I. A., & Abdel-Sattar, E. A. (2015). Anti-inflammatory activity of flavonoids from Chrozophora tinctoria. *Phytochemistry Letters*, *13*, 74-80. <https://doi.org/10.1016/j.phytol.2015.05.008>

Adamante, G., de Almeida, A. S., Rigo, F. K., da Silva Silveira, E., Coelho, Y. O., De Prá, S. D. T., & Trevisan, G. (2019). Diosmetin as a novel transient receptor potential vanilloid 1 antagonist with antinociceptive activity in mice. Life sciences, 216, 215-226. <https://doi.org/10.1016/j.lfs.2018.11.029>

Ahmadi, A., & Shadboorestan, A. (2016). Oxidative stress and cancer; the role of hesperidin, a citrus natural bioflavonoid, as a cancer chemoprotective agent. Nutrition and cancer, 68(1), 29-39. <https://doi.org/10.1080/01635581.2015.1078822>

Ahmed, S. I., Hayat, M. Q., Tahir, M., Mansoor, Q., Ismail, M., Keck, K., & Bates, R. B. (2016). Pharmacologically active flavonoids from the anticancer, antioxidant and antimicrobial extracts of Cassia angustifolia Vahl. BMC complementary and alternative medicine, 16(1), 1-9. <https://doi.org/10.1186/s12906-016-1443-z>

Alay, M., Sonmez, M., Sakin, A., Atmaca, M., Süleyman, H., YAZICI, G., ... & Altuner, D. (2022). The effects of taxifolin on neuropathy related with hyperglycemia and neuropathic pain in rats: A biochemical and histopathological evaluation. *Advances in Clinical and Experimental Medicine*, *31*(4). https://doi.org/[10.17219/acem/144002](https://doi.org/10.17219/acem/144002)

Ali, H., Kabir, N., Shah, M. R., Muhammad, A., Ali, S., Mehmood, S., & Jahan, A. (2016). Hepatoprotective activity of viscosine is mediated by attenuation of hepatic macrophages and iNOS expression in CCl4-intoxicated rats. Toxicology Research, 5(6), 1688-1698. <https://doi.org/10.1039/c6tx00165c>

Amanat, S., Shal, B., Seo, E. K., Ali, H., & Khan, S. (2022). Icariin attenuates cyclophosphamide-induced cystitis via down-regulation of NF-кB and up-regulation of Nrf-2/HO-1 signaling pathways in mice model. *International Immunopharmacology*, *106*, 108604.

<https://doi.org/10.1016/j.intimp.2022.108604>

An, J., Deed, R. C., Kilmartin, P. A., & Yu, W. (2023). Could collected chemical parameters Be utilized to build soft sensors capable of predicting the provenance, vintages, and price points of New Zealand Pinot noir wines simultaneously?. Foods, 12(2), 323. <https://doi.org/10.3390/foods12020323>

Anu Aravind, A. P., Asha, K. R. T., & Rameshkumar, K. B. (2016). Phytochemical analysis and antioxidant potential of the leaves of Garcinia travancorica Bedd. Natural product research, 30(2), 232-236. <https://doi.org/10.1080/14786419.2015.1043551>

Anuradha, R., & Sukumar, D. (2013). In vitro anti-inflammatory compound quercimeritrin isolated from tithonia diversifolia flowers by hrbc membrane stabilization. *World J Pharm Res*, *3*(1), 426-431. <https://wjpr.s3.ap-south-1.amazonaws.com/article_issue/138874376611%20WJPR%20706.pdf>

Aswar, M., Kute, P., Mahajan, S., Mahajan, U., Nerurkar, G., & Aswar, U. (2014). Protective effect of hesperetin in rat model of partial sciatic nerve ligation induced painful neuropathic pain: evidence of anti-inflammatory and anti-oxidative activity. Pharmacology Biochemistry and Behavior, 124, 101-107. <https://doi.org/10.1016/j.pbb.2014.05.013>

Bartella, L., Mazzotti, F., Talarico, I. R., De Luca, G., Santoro, I., Prejanò, M., & Di Donna, L. (2022). Structural Characterization of Peripolin and Study of Antioxidant Activity of HMG Flavonoids from Bergamot Fruit. Antioxidants, 11(10), 1847. <https://doi.org/10.3390/antiox11101847>

Basu, P., & Basu, A. (2020). In vitro and in vivo effects of flavonoids on peripheral neuropathic pain. *Molecules*, *25*(5), 1171. <https://doi.org/10.3390/molecules25051171>

Bi, H., Sun, Z., Chu, Q., Li, L., Guan, X., Zhou, Y., & Li, Z. (2019). Analgesic effects of astilbin partially via calcium channels through regulation on CaMKII. Food and Agricultural Immunology, 30(1), 309-319. <https://doi.org/10.1080/09540105.2019.1580677>

Borghi, S. M., Carvalho, T. T., Staurengo-Ferrari, L., Hohmann, M. S., Pinge-Filho, P., Casagrande, R., & Verri Jr, W. A. (2013). Vitexin inhibits inflammatory pain in mice by targeting TRPV1, oxidative stress, and cytokines. Journal of natural products, 76(6), 1141-1149. <https://doi.org/10.1021/np400222v>

Bouyahya, A., Guaouguaou, F. E., El Omari, N., El Menyiy, N., Balahbib, A., El-Shazly, M., & Bakri, Y. (2022). Anti-inflammatory and analgesic properties of Moroccan medicinal plants: Phytochemistry, in vitro and in vivo investigations, mechanism insights, clinical evidences and perspectives. Journal of Pharmaceutical Analysis, 12(1), 35-57. <https://doi.org/10.1016/j.jpha.2021.07.004>

Calderon-Rivera, A., Loya-Lopez, S., Gomez, K., & Khanna, R. (2022). Plant and fungi derived analgesic natural products targeting voltage-gated sodium and calcium channels. Channels, 16(1), 198-215. <https://doi.org/10.1080/19336950.2022.2103234>

Carbonneau, M. A., Cisse, M., Mora-Soumille, N., Dairi, S., Rosa, M., Michel, F., & Dangles, O. (2014). Antioxidant properties of 3-deoxyanthocyanidins and polyphenolic extracts from Côte d’Ivoire’s red and white sorghums assessed by ORAC and in vitro LDL oxidisability tests. Food chemistry, 145, 701-709. <https://doi.org/10.1016/j.foodchem.2013.07.025>

Chan, B. C. L., Barua, N., Lau, C. B. S., Leung, P. C., Fung, K. P., & Ip, M. (2023). Enhancing Antibiotics Efficacy by Combination of Kuraridin and Epicatechin Gallate with Antimicrobials against Methicillin-Resistant Staphylococcus aureus. Antibiotics, 12(1), 117. <https://doi.org/10.3390/antibiotics12010117>

Chen, G. L., Fan, M. X., Wu, J. L., Li, N., & Guo, M. Q. (2019). Antioxidant and anti-inflammatory properties of flavonoids from lotus plumule. Food chemistry, 277, 706-712. <https://doi.org/10.1016/j.foodchem.2018.11.040>

Chen, L., Teng, H., Jia, Z., Battino, M., Miron, A., Yu, Z., & Xiao, J. (2018). Intracellular signaling pathways of inflammation modulated by dietary flavonoids: The most recent evidence. Critical reviews in food science and nutrition, 58(17), 2908-2924. <https://doi.org/10.1080/10408398.2017.1345853>

Chen, M., Zhao, Y., Chen, F., Zhao, Z., Yu, S., & Abbasi, A. M. (2015). An HPLC-DPPH method for antioxidant activity from sugarcane molasses. *Sugar Industry/Zuckerindustrie*, *140*(10), 632-639. <https://www.cabdirect.org/cabdirect/abstract/20153345185>

Chen, S., Xiong, J., Zhan, Y., Liu, W., & Wang, X. (2015). Wogonin inhibits LPS-induced inflammatory responses in rat dorsal root ganglion neurons via inhibiting TLR4–MyD88–TAK1-mediated NF-κB and MAPK signaling pathway. Cellular and molecular neurobiology, 35, 523-531. <https://doi.org/10.1007/s10571-014-0148-4>

Chen, X. M., Tait, A. R., & Kitts, D. D. (2017). Flavonoid composition of orange peel and its association with antioxidant and anti-inflammatory activities. Food chemistry, 218, 15-21. <https://doi.org/10.1016/j.foodchem.2016.09.016>

Chen, X., Mukwaya, E., Wong, M. S., & Zhang, Y. (2014). A systematic review on biological activities of prenylated flavonoids. *Pharmaceutical biology*, *52*(5), 655-660. <https://doi.org/10.3109/13880209.2013.853809>

Chen, Y., Zheng, Y., Zhou, Z., & Wang, J. (2018). Baicalein alleviates tubular-interstitial nephritis in vivo and in vitro by down-regulating NF-κB and MAPK pathways. Brazilian Journal of Medical and Biological Research, 51. <https://doi.org/10.1590/1414-431X20187476>

Cherng, C. H., Lee, K. C., Chien, C. C., Chou, K. Y., Cheng, Y. C., Hsin, S. T., ... & Wong, C. S. (2014). Baicalin ameliorates neuropathic pain by suppressing HDAC1 expression in the spinal cord of spinal nerve ligation rats. Journal of the Formosan Medical Association, 113(8), 513-520. <https://doi.org/10.1016/j.jfma.2013.04.007>

Chopade, A. R., Sayyad, F. J., & Pore, Y. V. (2015). Molecular docking studies of phytocompounds from the phyllanthus species as potential chronic pain modulators. Scientia pharmaceutica, 83(2), 243-267. <https://doi.org/10.3797/scipharm.1408-10>

Choucry, M. A., Shalabi, A. A., El Halawany, A. M., El-Sakhawy, F. S., Zaiter, A., Morita, H., & Abdel-Sattar, E. (2021). New pregnane glycosides isolated from Caralluma hexagona lavranos as inhibitors of α-glucosidase, pancreatic lipase, and advanced glycation end products formation. ACS omega, 6(29), 18881-18889. <https://doi.org/10.1021/acsomega.1c02056>

Choy, K. W., Murugan, D., Leong, X. F., Abas, R., Alias, A., & Mustafa, M. R. (2019). Flavonoids as natural anti-inflammatory agents targeting nuclear factor-kappa B (NFκB) signaling in cardiovascular diseases: A mini review. *Frontiers in pharmacology*, *10*, 1295. <https://doi.org/10.3389/fphar.2019.01295>

Chu, X., Ci, X., Wei, M., Yang, X., Cao, Q., Guan, M., & Deng, X. (2012). Licochalcone a inhibits lipopolysaccharide-induced inflammatory response in vitro and in vivo. *Journal of agricultural and food chemistry*, *60*(15), 3947-3954. <https://doi.org/10.1021/jf2051587>

Cialdella-Kam, L., Nieman, D. C., Knab, A. M., Shanely, R. A., Meaney, M. P., Jin, F., & Ghosh, S. (2016). A mixed Flavonoid-Fish oil supplement induces immune-enhancing and anti-inflammatory transcriptomic changes in adult obese and overweight women—A randomized controlled trial. Nutrients, 8(5), 277. <https://doi.org/10.3390/nu8050277>

Daveri, E., Cremonini, E., Mastaloudis, A., Hester, S. N., Wood, S. M., Waterhouse, A. L., & Oteiza, P. I. (2018). Cyanidin and delphinidin modulate inflammation and altered redox signaling improving insulin resistance in high fat-fed mice. Redox Biology, 18, 16-24. <https://doi.org/10.1016/j.redox.2018.05.012>

Denaro, M., Smeriglio, A., & Trombetta, D. (2021). Antioxidant and anti-inflammatory activity of citrus flavanones mix and its stability after in vitro simulated digestion. *Antioxidants*, *10*(2), 140. <https://doi.org/10.3390/antiox10020140>

Deng, Y., Ma, J., Weng, X., Wang, Y., Li, M., Yang, T., & Shang, J. (2021). Kaempferol-3-o-glucuronide ameliorates non-alcoholic steatohepatitis in high-cholesterol-diet-induced larval zebrafish and hepg2 cell models via regulating oxidation stress. *Life*, *11*(5), 445. <https://doi.org/10.3390/life11050445>

De Spirt, S., Eckers, A., Wehrend, C., Micoogullari, M., Sies, H., Stahl, W., & Steinbrenner, H. (2016). Interplay between the chalcone cardamonin and selenium in the biosynthesis of Nrf2-regulated antioxidant enzymes in intestinal Caco-2 cells. *Free Radical Biology and Medicine*, *91*, 164-171. <https://doi.org/10.1016/j.freeradbiomed.2015.12.011>

Dinda, B., SilSarma, I., Dinda, M., & Rudrapaul, P. (2015). Oroxylum indicum (L.) Kurz, an important Asian traditional medicine: from traditional uses to scientific data for its commercial exploitation. Journal of ethnopharmacology, 161, 255-278. <https://doi.org/10.1016/j.jep.2014.12.027>

Dogan, Z., Telli, G., Tel, B. C., & Saracoglu, I. (2022). Scutellaria brevibracteata Stapf and active principles with anti-inflammatory effects through regulation of NF-κB/COX-2/iNOS pathways. Fitoterapia, 158, 105159. <https://doi.org/10.1016/j.fitote.2022.105159>

Dong, X., Huang, Y., Wang, Y., & He, X. (2019). Anti-inflammatory and antioxidant jasmonates and flavonoids from lychee seeds. Journal of Functional Foods, 54, 74-80. <https://doi.org/10.1016/j.jff.2018.12.040>

Eom, S., Lee, B. B., Lee, S., Park, Y., Yeom, H. D., Kim, T. H., & Lee, J. H. (2021). Antioxidative and analgesic effects of naringin through selective inhibition of transient receptor potential vanilloid member 1. Antioxidants, 11(1), 64. <https://doi.org/10.3390/antiox11010064>

Filannino, P., Cavoski, I., Thlien, N., Vincentini, O., De Angelis, M., Silano, M., & Di Cagno, R. (2016). Lactic acid fermentation of cactus cladodes (Opuntia ficus-indica L.) generates flavonoid derivatives with antioxidant and anti-inflammatory properties. PLoS One, 11(3), e0152575. <https://doi.org/10.1371/journal.pone.0152575>

Feng, J. H., Lee, H. J., Kim, S. B., Jung, J. S., Lim, S. S., & Suh, H. W. (2019). Antinociceptive effect of single components isolated from Agrimonia pilosa Ledeb. Extract. Scientia Pharmaceutica, 87(3), 18. <https://doi.org/10.3390/scipharm87030018>

Fernández, J., Silván, B., Entrialgo-Cadierno, R., Villar, C. J., Capasso, R., Uranga, J. A., & Abalo, R. (2021). Antiproliferative and palliative activity of flavonoids in colorectal cancer. *Biomedicine & Pharmacotherapy*, *143*, 112241. <https://doi.org/10.1016/j.biopha.2021.112241>

Furusawa, J. I., Funakoshi-Tago, M., Mashino, T., Tago, K., Inoue, H., Sonoda, Y., & Kasahara, T. (2009). Glycyrrhiza inflata-derived chalcones, Licochalcone A, Licochalcone B and Licochalcone D, inhibit phosphorylation of NF-κB p65 in LPS signaling pathway. *International immunopharmacology*, *9*(4), 499-507. <https://doi.org/10.1016/j.intimp.2009.01.031>

Fu, Y., Chen, J., Li, Y. J., Zheng, Y. F., & Li, P. (2013). Antioxidant and anti-inflammatory activities of six flavonoids separated from licorice. *Food chemistry*, *141*(2), 1063-1071. <https://doi.org/10.1016/j.foodchem.2013.03.089>

Ge, H., Guan, S., Shen, Y., Sun, M., Hao, Y., He, L., & Gao, Y. (2019). Dihydromyricetin affects BDNF levels in the nervous system in rats with comorbid diabetic neuropathic pain and depression. Scientific reports, 9(1), 14619. <https://doi.org/10.1038/s41598-019-51124-w>

Ginwala, R., Bhavsar, R., Chigbu, D. G. I., Jain, P., & Khan, Z. K. (2019). Potential role of flavonoids in treating chronic inflammatory diseases with a special focus on the anti-inflammatory activity of apigenin. Antioxidants, 8(2), 35. <https://doi.org/10.3390/antiox8020035>

Gutierrez-Zepeda, A., Santell, R., Wu, Z., Brown, M., Wu, Y., Khan, I., ... & Luo, Y. (2005). Soy isoflavone glycitein protects against beta amyloid-induced toxicity and oxidative stress in transgenic Caenorhabditis elegans. BMC neuroscience, 6(1), 1-9. https://bmcneurosci.biomedcentral.com/articles/10.1186/1471-2202-6-54

Habtemariam, S. (2023). The Molecular Pharmacology of Phloretin: Anti-Inflammatory Mechanisms of Action. *Biomedicines*, *11*(1), 143. <https://doi.org/10.3390/biomedicines11010143>

Hagenacker, T., Hillebrand, I., Wissmann, A., Büsselberg, D., & Schäfers, M. (2010). Anti-allodynic effect of the flavonoid myricetin in a rat model of neuropathic pain: involvement of p38 and protein kinase C mediated modulation of Ca2+ channels. European Journal of Pain, 14(10), 992-998. <https://doi.org/10.1016/j.ejpain.2010.04.005>

Hanáková, Z., Hosek, J., Kutil, Z., Temml, V., Landa, P., Vanek, T., & Smejkal, K. (2017). Anti-inflammatory activity of natural geranylated flavonoids: cyclooxygenase and lipoxygenase inhibitory properties and proteomic analysis. *Journal of natural products*, *80*(4), 999-1006. <https://doi.org/10.1021/acs.jnatprod.6b01011>

He, M., Min, J. W., Kong, W. L., He, X. H., Li, J. X., & Peng, B. W. (2016). A review on the pharmacological effects of vitexin and isovitexin. Fitoterapia, 115, 74-85. <https://doi.org/10.1016/j.fitote.2016.09.011>

Hernández-Rodríguez, P., Baquero, L. P., & Larrota, H. R. (2019). Flavonoids: Potential therapeutic agents by their antioxidant capacity. In Bioactive compounds (pp. 265-288). Woodhead Publishing. <https://doi.org/10.1016/B978-0-12-814774-0.00014-1>

Hou, Y., Li, G., Wang, J., Pan, Y., Jiao, K., Du, J., ... & Li, N. (2017). Okanin, effective constituent of the flower tea Coreopsis tinctoria, attenuates LPS-induced microglial activation through inhibition of the TLR4/NF-κB signaling pathways. *Scientific reports*, *7*(1), 45705. <https://doi.org/10.1038/srep45705>

Huang, W. Y., Liu, Y. M., Wang, J., Wang, X. N., & Li, C. Y. (2014). Anti-inflammatory effect of the blueberry anthocyanins malvidin-3-glucoside and malvidin-3-galactoside in endothelial cells. Molecules, 19(8), 12827-12841. <https://doi.org/10.3390/molecules190812827>

Hyun, S. K., Lee, W. H., Jeong, D. M., Kim, Y., & Choi, J. S. (2008). Inhibitory effects of kurarinol, kuraridinol, and trifolirhizin from Sophora flavescens on tyrosinase and melanin synthesis. Biological and Pharmaceutical Bulletin, 31(1), 154-158. <https://doi.org/10.1248/bpb.31.154>

Huyut, Z., Beydemir, Ş., & Gülçin, İ. (2017). Antioxidant and antiradical properties of selected flavonoids and phenolic compounds. Biochemistry research international, 2017. <https://doi.org/10.1155/2017/7616791>

Issac, P. K., Guru, A., Velayutham, M., Pachaiappan, R., Arasu, M. V., Al-Dhabi, N. A., & Arockiaraj, J. (2021). Oxidative stress induced antioxidant and neurotoxicity demonstrated in vivo zebrafish embryo or larval model and their normalization due to morin showing therapeutic implications. *Life sciences*, *283*, 119864. <https://doi.org/10.1016/j.lfs.2021.119864>

Jantas, D., Malarz, J., Le, T. N., & Stojakowska, A. (2021). Neuroprotective properties of kempferol derivatives from Maesa membranacea against oxidative stress-induced cell damage: An association with cathepsin D inhibition and PI3K/Akt activation. International Journal of Molecular Sciences, 22(19), 10363. <https://doi.org/10.3390/ijms221910363>

Jiang, H., Zhan, W. Q., Liu, X., & Jiang, S. X. (2008). Antioxidant activities of extracts and flavonoid compounds from Oxytropis falcate Bunge. *Natural product research*, *22*(18), 1650-1656. <https://doi.org/10.1080/14786410701875686>

Jiang, L., Wu, Q., Yang, T., & Yang, N. (2016). Xanthotoxol attenuates neuropathic pain in a rat model of chronic constriction injury. *Int J Clin Exp Med*, *9*(11), 21556-21561. https://e-century.us/files/ijcem/9/11/ijcem0018049.pdf

Jia, Y., He, W., Zhang, H., He, L., Wang, Y., Zhang, T., & Qian, Y. (2020). Morusin ameliorates IL-1β-induced chondrocyte inflammation and osteoarthritis via NF-κB signal pathway. Drug Design, Development and Therapy, 1227-1240. https://doi.org/10.2147/dddt.s244462

Jo, A., Yoo, H. J., & Lee, M. (2019). Robustaflavone isolated from Nandina domestica using bioactivity-guided fractionation downregulates inflammatory mediators. Molecules, 24(9), 1789. <https://doi.org/10.3390/molecules24091789>

Jucá, M. M., Cysne Filho, F. M. S., de Almeida, J. C., Mesquita, D. D. S., Barriga, J. R. D. M., Dias, K. C. F., & Vasconcelos, S. M. M. (2020). Flavonoids: biological activities and therapeutic potential. Natural product research, 34(5), 692-705. <https://doi.org/10.1080/14786419.2018.1493588>

Kang, M. R., Park, K. H., Oh, S. J., Yun, J., Lee, C. W., Lee, M. Y., & Kang, J. S. (2015). Cardiovascular protective effect of glabridin: Implications in LDL oxidation and inflammation. International Immunopharmacology, 29(2), 914-918. <https://doi.org/10.1016/j.intimp.2015.10.020>

Kashino, Y., Murota, K., Matsuda, N., Tomotake, M., Hamano, T., Mukai, R., & Terao, J. (2015). Effect of processed onions on the plasma concentration of quercetin in rats and humans. *Journal of Food Science*, *80*(11), H2597-H2602. <https://doi.org/10.1111/1750-3841.13079>

Kiani, R., Arzani, A., & Mirmohammady Maibody, S. A. M. (2021). Polyphenols, flavonoids, and antioxidant activity involved in salt tolerance in wheat, Aegilops cylindrica and their amphidiploids. *Frontiers in plant science*, *12*, 646221. <https://doi.org/10.3389/fpls.2021.646221>

Kim, M. H., Kwon, S. Y., Woo, S. Y., Seo, W. D., & Kim, D. Y. (2021). Antioxidative effects of chrysoeriol via activation of the Nrf2 signaling pathway and modulation of mitochondrial function. Molecules, 26(2), 313. <https://doi.org/10.3390/molecules26020313>

Kim, S. J., Um, J. Y., Hong, S. H., & Lee, J. Y. (2011). Anti-inflammatory activity of hyperoside through the suppression of nuclear factor-κB activation in mouse peritoneal macrophages. The American journal of Chinese medicine, 39(01), 171-181. <https://doi.org/10.1142/S0192415X11008737>

Kirschweng, B., Bencze, K., Sárközi, M., Hégely, B., Samu, G., Hári, J., & Pukánszky, B. (2016). Melt stabilization of polyethylene with dihydromyricetin, a natural antioxidant. *Polymer Degradation and Stability*, *133*, 192-200. <https://doi.org/10.1016/j.polymdegradstab.2016.08.016>

Khan, A., Ikram, M., Hahm, J. R., & Kim, M. O. (2020). Antioxidant and anti-inflammatory effects of citrus flavonoid hesperetin: Special focus on neurological disorders. Antioxidants, 9(7), 609. <https://doi.org/10.3390/antiox9070609>

Khan, J., Deb, P. K., Priya, S., Medina, K. D., Devi, R., Walode, S. G., & Rudrapal, M. (2021). Dietary flavonoids: Cardioprotective potential with antioxidant effects and their pharmacokinetic, toxicological and therapeutic concerns. Molecules, 26(13), 4021. <https://doi.org/10.3390/molecules26134021>

Komakech, R., Kim, Y. G., Matsabisa, G. M., & Kang, Y. (2019). Anti-inflammatory and analgesic potential of Tamarindus indica Linn.(Fabaceae): a narrative review. Integrative Medicine Research, 8(3), 181-186. <https://doi.org/10.1016/j.imr.2019.07.002>

Kupeli, E., Orhan, I., Toker, G., & Yesilada, E. (2006). Anti-inflammatory and antinociceptive potential of Maclura pomifera (Rafin.) Schneider fruit extracts and its major isoflavonoids, scandenone and auriculasin. *Journal of ethnopharmacology*, *107*(2), 169-174. <https://doi.org/10.1016/j.jep.2006.02.021>

Kut, K., Bartosz, G., Soszyński, M., & Sadowska-Bartosz, I. (2022). Antioxidant properties of hispidulin. Natural Product Research, 36(24), 6401-6404. <https://doi.org/10.1080/14786419.2022.2032050>

Lai, H. C., Lu, C. H., Wong, C. S., Lin, B. F., Chan, S. M., Kuo, C. Y., & Wu, Z. F. (2018). Baicalein attenuates neuropathic pain and improves sciatic nerve function recovery in rats with partial sciatic nerve transection. Journal of the Chinese Medical Association, 81(11), 955-963. <https://doi.org/10.1016/j.jcma.2018.03.014>

Lee, E. J., Kim, S. Y., Hyun, J. W., Min, S. W., Kim, D. H., & Kim, H. S. (2010). Glycitein inhibits glioma cell invasion through down-regulation of MMP-3 and MMP-9 gene expression. Chemico-biological interactions, 185(1), 18-24. <https://doi.org/10.1016/j.cbi.2010.02.037>

Lee, J., Choi, J. W., Sohng, J. K., Pandey, R. P., & Park, Y. I. (2016). The immunostimulating activity of quercetin 3-O-xyloside in murine macrophages via activation of the ASK1/MAPK/NF-κB signaling pathway. International Immunopharmacology, 31, 88-97. <https://doi.org/10.1016/j.intimp.2015.12.008>

Lee, J. W., Kim, N. H., Kim, J. Y., Park, J. H., Shin, S. Y., Kwon, Y. S., & Chun, W. (2013). Aromadendrin inhibits lipopolysaccharide-induced nuclear translocation of NF-κB and phosphorylation of JNK in RAW 264.7 macrophage cells. Biomolecules & Therapeutics, 21(3), 216. https://doi.org/[10.4062/biomolther.2013.023](https://doi.org/10.4062%2Fbiomolther.2013.023)

Lee, L. S., Choi, E. J., Kim, C. H., Sung, J. M., Kim, Y. B., Seo, D. H., & Park, J. D. (2016). Contribution of flavonoids to the antioxidant properties of common and tartary buckwheat. Journal of Cereal Science, 68, 181-186. <https://doi.org/10.1016/j.jcs.2015.07.005>

Lee, W., Ku, S. K., & Bae, J. S. (2014). Vascular barrier protective effects of orientin and isoorientin in LPS-induced inflammation in vitro and in vivo. *Vascular Pharmacology*, *62*(1), 3-14. <https://doi.org/10.1016/j.vph.2014.04.006>

Li, J., Tong, D., Liu, J., Chen, F., & Shen, Y. (2016). Oroxylin A attenuates cigarette smoke-induced lung inflammation by activating Nrf2. International immunopharmacology, 40, 524-529. <https://doi.org/10.1016/j.intimp.2016.10.011>

Lin, H. Y., Chang, T. C., & Chang, S. T. (2018). A review of antioxidant and pharmacological properties of phenolic compounds in Acacia confusa. Journal of traditional and complementary medicine, 8(4), 443-450. <https://doi.org/10.1016/j.jtcme.2018.05.002>

Li, P., Yu, C., Zeng, F. S., Fu, X., Yuan, X. J., Wang, Q., & Sun, Q. S. (2021). Licochalcone A attenuates chronic neuropathic pain in rats by inhibiting microglia activation and inflammation. *Neurochemical research*, *46*, 1112-1118. <https://doi.org/10.1007/s11064-021-03244-x>

Liu, C., Liu, H., Zhang, F., Zheng, J., & Wei, F. (2019). Scutellarin Mitigates Cancer-Induced Bone Pain by Suppressing CaMKII/CREB Pathway in Rat Models. Current Topics in Nutraceutical Research, 17(3). <https://doi.org/10.37290/ctnr2641-452x.17:249-253>

Liu, L., Zuo, Z., Lu, S., Liu, A., & Liu, X. (2017). Naringin attenuates diabetic retinopathy by inhibiting inflammation, oxidative stress and NF-κB activation in vivo and in vitro. Iranian journal of basic medical sciences, 20(7), 813. https://doi.org/[10.22038/IJBMS.2017.9017](https://doi.org/10.22038/ijbms.2017.9017)

Lim, E. Y., Lee, C., & Kim, Y. T. (2022). The antinociceptive potential of Camellia japonica leaf extract, (−)-epicatechin, and rutin against chronic constriction injury-induced neuropathic pain in rats. Antioxidants, 11(2), 410. <https://doi.org/10.3390/antiox11020410>

Li, Q., Tian, Z., Wang, M., Kou, J., Wang, C., Rong, X., & Pang, X. (2019). Luteoloside attenuates neuroinflammation in focal cerebral ischemia in rats via regulation of the PPARγ/Nrf2/NF-κB signaling pathway. *International Immunopharmacology*, *66*, 309-316. <https://doi.org/10.1016/j.intimp.2018.11.044>

Li, X., Xu, Y., Li, H., Jia, L., Wang, J., Liang, S., & Asakawa, T. (2021). Verification of pain-related neuromodulation mechanisms of icariin in knee osteoarthritis. Biomedicine & Pharmacotherapy, 144, 112259. <https://doi.org/10.1016/j.biopha.2021.112259>

Luzzi, R., Guimarães, C. L., Verdi, L. G., Simionatto, E. L., Delle Monache, F., Yunes, R. A., & Cechinel-Filho, V. (1997). Isolation of biflavonoids with analgesic activity from Rheedia gardneriana leaves. Phytomedicine, 4(2), 141-144. <https://doi.org/10.1016/S0944-7113(97)80060-8>

Makanjuola, S. B., Ogundaini, A. O., Ajonuma, L. C., & Dosunmu, A. (2018). Apigenin and apigeninidin isolates from the Sorghum bicolor leaf targets inflammation via cyclooxygenase‐2 and prostaglandin‐E2 blockade. International journal of rheumatic diseases, 21(8), 1487-1495. <https://doi.org/10.1111/1756-185X.13355>

Mamdouh, M. A., & Monira, A. A. E. K. (2004). The influence of naringin on the oxidative state of rats with streptozotocin-induced acute hyperglycaemia. Zeitschrift für Naturforschung C, 59(9-10), 726-733. <https://doi.org/10.1515/znc-2004-9-1018>

Mangla, B., Kohli, K., & Rabiu, S. (2021). Review of medicinal uses, phytochemistry, pharmacological properties, extraction methods and toxicology of Lannea microcarpa (African Grapes). Current Traditional Medicine, 7(1), 125-137. <https://doi.org/10.2174/2215083805666190626095609>

Mansoor, H. U. H., Ahmed, A., & Rasool, F. (2023). The inhibitory potential of chemical constituents of Ficus carica targeting interleukin‐6 (IL‐6) mediated inflammation. Cell Biochemistry and Function. <https://doi.org/10.1002/cbf.3813>

Ma, Z., Ji, W., Fu, Q., & Ma, S. (2013). Formononetin inhibited the inflammation of LPS-induced acute lung injury in mice associated with induction of PPAR gamma expression. *Inflammation*, *36*, 1560-1566. <https://link.springer.com/article/10.1007/s10753-013-9700-5>

Mencherini, T., Cau, A., Bianco, G., Loggia, R. D., Aquino, R. P., & Autore, G. (2007). An extract of Apium graveolens var. dulce leaves: Structure of the major constituent, apiin, and its anti‐inflammatory properties. Journal of pharmacy and pharmacology, 59(6), 891-897. <https://doi.org/10.1211/jpp.59.6.0016>

Michel, T. K., Ottoh, A. A., Chukwunonye, U. C. E., Obodoike, E. C., Christopher, O., & Mmaduakolam, I. M. (2016). Bio-flavonoids and Garcinoic acid from Garcinia kola seeds with promising anti-inflammatory potentials. Pharmacognosy Journal, 8(1), 56. <https://doi.org/10.5530/pj.2016.1.12>

Mondal, A., Maity, T. K., & Bishayee, A. (2019). Analgesic and anti-inflammatory activities of quercetin-3-methoxy-4′-glucosyl-7-glucoside isolated from Indian medicinal plant Melothria heterophylla. *Medicines*, *6*(2), 59. <https://doi.org/10.3390/medicines6020059>

Moscatelli, V., Hnatyszyn, O., Acevedo, C., Megías, J., Alcaraz, M. J., & Ferraro, G. (2006). Flavonoids from Artemisia copa with anti-inflammatory activity. Planta medica, 72(01), 72-74. <https://doi.org/10.1055/s-2005-873177>

Mottaghipisheh, J., Taghrir, H., Boveiri Dehsheikh, A., Zomorodian, K., Irajie, C., Mahmoodi Sourestani, M., & Iraji, A. (2021). Linarin, a glycosylated flavonoid, with potential therapeutic attributes: A comprehensive review. Pharmaceuticals, 14(11), 1104. <https://doi.org/10.3390/ph14111104>

Nagula, R. L., & Wairkar, S. (2019). Recent advances in topical delivery of flavonoids: A review. Journal of controlled release, 296, 190-201. <https://doi.org/10.1016/j.jconrel.2019.01.029>

Negm, W. A., El-Kadem, A. H., Hussein, I. A., & Alqahtani, M. J. (2022). The Mechanistic Perspective of Bilobetin Protective Effects against Cisplatin-Induced Testicular Toxicity: Role of Nrf-2/Keap-1 Signaling, Inflammation, and Apoptosis. Biomedicines, 10(5), 1134. <https://doi.org/10.3390/biomedicines10051134>

Nile, S. H., Keum, Y. S., Nile, A. S., Jalde, S. S., & Patel, R. V. (2018). Antioxidant, anti‐inflammatory, and enzyme inhibitory activity of natural plant flavonoids and their synthesized derivatives. Journal of biochemical and molecular toxicology, 32(1), e22002. <https://doi.org/10.1002/jbt.22002>

Ninfali, P., Antonini, E., Frati, A., & Scarpa, E. S. (2017). C‐glycosyl flavonoids from Beta vulgaris cicla and betalains from Beta vulgaris rubra: antioxidant, anticancer and antiinflammatory activities—A review. Phytotherapy Research, 31(6), 871-884. <https://doi.org/10.1002/ptr.5819>

Oracz, J., Kowalski, S., Żyżelewicz, D., Kowalska, G., Gumul, D., Kulbat-Warycha, K., & Areczuk, A. (2023). The Influence of Microwave-Assisted Extraction on the Phenolic Compound Profile and Biological Activities of Extracts from Selected Scutellaria Species. Molecules, 28(9), 3877. <https://doi.org/10.3390/molecules28093877>

Park, C. M., & Song, Y. S. (2019). Luteolin and luteolin-7-O-glucoside protect against acute liver injury through regulation of inflammatory mediators and antioxidative enzymes in GalN/LPS-induced hepatitic ICR mice. *Nutrition research and practice*, *13*(6), 473-479. <https://doi.org/10.4162/nrp.2019.13.6.473>

Park, M. Y., Ha, S. E., Kim, H. H., Bhosale, P. B., Abusaliya, A., Jeong, S. H., & Kim, G. S. (2022). Scutellarein inhibits LPS-induced inflammation through NF-κB/MAPKs signaling pathway in RAW264. 7 cells. Molecules, 27(12), 3782. <https://doi.org/10.3390/molecules27123782>

Parlar, A., Arslan, S. O., & Çam, S. A. (2020). Glabridin alleviates inflammation and nociception in rodents by activating BKCa channels and reducing NO levels. Biological and Pharmaceutical Bulletin, 43(5), 884-897. <https://doi.org/10.1248/bpb.b20-00038>

Peng, L., Wen, L., Shi, Q. F., Gao, F., Huang, B., Meng, J., & Wang, C. M. (2020). Scutellarin ameliorates pulmonary fibrosis through inhibiting NF-κB/NLRP3-mediated epithelial–mesenchymal transition and inflammation. Cell death & disease, 11(11), 978. <https://doi.org/10.1038/s41419-020-03178-2>

Pinho-Ribeiro, F. A., Zarpelon, A. C., Mizokami, S. S., Borghi, S. M., Bordignon, J., Silva, R. L., & Verri Jr, W. A. (2016). The citrus flavonone naringenin reduces lipopolysaccharide-induced inflammatory pain and leukocyte recruitment by inhibiting NF-κB activation. The Journal of nutritional biochemistry, 33, 8-14. <https://doi.org/10.1016/j.jnutbio.2016.03.013>

Rabidas, S. S., Prakash, C., Tyagi, J., Suryavanshi, J., Kumar, P., Bhattacharya, J., & Sharma, D. (2023). A Comprehensive Review on Anti-Inflammatory Response of Flavonoids in Experimentally-Induced Epileptic Seizures. *Brain Sciences*, *13*(1), 102. <https://doi.org/10.3390/brainsci13010102>

Ramesh, M., Rao, Y. N., Rao, A. A., Prabhakar, M. C., Rao, C. S., Muralidhar, N., & Reddy, B. M. (1998). Antinociceptive and anti-inflammatory activity of a flavonoid isolated from Caralluma attenuata. Journal of ethnopharmacology, 62(1), 63-66. <https://doi.org/10.1016/S0378-8741(98)00048-8>

Ramešová, Š., Degano, I., & Sokolová, R. (2017). The oxidative decomposition of natural bioactive compound rhamnetin. Journal of Electroanalytical Chemistry, 788, 125-130. <https://doi.org/10.1016/j.jelechem.2017.01.054>

Ribeiro, D., Freitas, M., Lima, J. L., & Fernandes, E. (2015). Proinflammatory pathways: the modulation by flavonoids. Medicinal research reviews, 35(5), 877-936. <https://doi.org/10.1002/med.21347>

Rho, H. S., Ghimeray, A. K., Yoo, D. S., Ahn, S. M., Kwon, S. S., Lee, K. H., & Cho, J. Y. (2011). Kaempferol and kaempferol rhamnosides with depigmenting and anti-inflammatory properties. *Molecules*, *16*(4), 3338-3344. <https://doi.org/10.3390/molecules16043338>

Sanghyun, L., Mi, J. C., Ji, M. C., Sullim, L., Hyun, Y. K., & Eun, J. C. (2012). Flavonoids from Taraxacum coreanum protect from radical-induced oxidative damage. *Journal of Medicinal Plants Research*, *6*(40), 5377-5384. <https://doi.org/10.5897/jmpr12.750>

Sauer, R. S., Krummenacher, I., Bankoglu, E. E., Yang, S., Oehler, B., Schöppler, F., & Rittner, H. L. (2021). Stabilization of delphinidin in complex with sulfobutylether-β-cyclodextrin allows for antinociception in inflammatory pain. Antioxidants & Redox Signaling, 34(16), 1260-1279. <https://doi.org/10.1089/ars.2019.7957>

Schempp, H., Vogel, S., Hückelhoven, R., & Heilmann, J. (2010). Re-evaluation of superoxide scavenging capacity of xanthohumol. *Free radical research*, *44*(12), 1435-1444. <https://doi.org/10.3109/10715762.2010.515216>

Seo, J. Y., Pandey, R. P., Lee, J., Sohng, J. K., Namkung, W., & Park, Y. I. (2019). Quercetin 3-O-xyloside ameliorates acute pancreatitis in vitro via the reduction of ER stress and enhancement of apoptosis. Phytomedicine, 55, 40-49. <https://doi.org/10.1016/j.phymed.2018.07.011>

Shimoda, K., Hamada, H., & Hamada, H. (2011). Synthesis of xylooligosaccharides of daidzein and their anti-oxidant and anti-allergic activities. International Journal of Molecular Sciences, 12(9), 5616-5625. <https://doi.org/10.3390/ijms12095616>

Shi, S., Li, J., Zhao, X., Liu, Q., & Song, S. J. (2021). A comprehensive review: Biological activity, modification and synthetic methodologies of prenylated flavonoids. *Phytochemistry*, *191*, 112895. <https://doi.org/10.1016/j.phytochem.2021.112895>

Shi, Y., Wu, D., Sun, Z., Yang, J., Chai, H., Tang, L., & Guo, Y. (2012). Analgesic and uterine relaxant effects of isoliquiritigenin, a flavone from Glycyrrhiza glabra. *Phytotherapy Research*, *26*(9), 1410-1417. <https://doi.org/10.1002/ptr.3715>

Silva, B., Biluca, F. C., Gonzaga, L. V., Fett, R., Dalmarco, E. M., Caon, T., & Costa, A. C. O. (2021). In vitro anti-inflammatory properties of honey flavonoids: A review. Food Research International, 141, 110086. <https://doi.org/10.1016/j.foodres.2020.110086>

Someya, S., Yoshiki, Y., & Okubo, K. (2002). Antioxidant compounds from bananas (Musa Cavendish). Food chemistry, 79(3), 351-354. <https://doi.org/10.1016/S0308-8146(02)00186-3>

Song, B., Shen, X., Tong, C., Zhang, S., Chen, Q., Li, Y., & Li, S. (2023). Gossypin: A flavonoid with diverse pharmacological effects. Chemical Biology & Drug Design, 101(1), 131-137. <https://doi.org/10.1111/cbdd.14152>

Soromou, L. W., Chu, X., Jiang, L., Wei, M., Huo, M., Chen, N., & Deng, X. (2012). In vitro and in vivo protection provided by pinocembrin against lipopolysaccharide-induced inflammatory responses. International immunopharmacology, 14(1), 66-74. <https://doi.org/10.1016/j.intimp.2012.06.009>

Spagnuolo, C., Moccia, S., & Russo, G. L. (2018). Anti-inflammatory effects of flavonoids in neurodegenerative disorders. European journal of medicinal chemistry, 153, 105-115. <https://doi.org/10.1016/j.ejmech.2017.09.001> Sun, Y., Liu, S., Yang, S., Chen, C., Yang, Y., Lin, M., & Chen, N. (2022). Mechanism of dihydromyricetin on inflammatory diseases. Frontiers in Pharmacology, 12, 794563. <https://doi.org/10.3389/fphar.2021.794563>

Tatli, I. I., Akdemir, Z. S., Yesilada, E., & Küpeli, E. (2008). Anti-inflammatory and antinociceptive potential of major phenolics from Verbascum salviifolium Boiss. Zeitschrift für Naturforschung C, 63(3-4), 196-202. <https://doi.org/10.1515/znc-2008-3-406>

Toker, G., Küpeli, E., Memisoğlu, M., & Yesilada, E. (2004). Flavonoids with antinociceptive and anti-inflammatory activities from the leaves of Tilia argentea (silver linden). *Journal of ethnopharmacology*, *95*(2-3), 393-397. <https://doi.org/10.1016/j.jep.2004.08.008>

Tsai, K. C., Zhang, Y. X., Kao, H. Y., Fung, K. M., & Tseng, T. S. (2022). Pharmacophore-driven identification of human glutaminyl cyclase inhibitors from foods, plants and herbs unveils the bioactive property and potential of Azaleatin in the treatment of Alzheimer's disease. Food & Function, 13(24), 12632-12647. <https://doi.org/10.1039/d2fo02507h>

Vendidandala, N. R., Yin, T. P., Nelli, G., Pasupuleti, V. R., Nyamathulla, S., & Mokhtar, S. I. (2021). Gallocatechin‑silver nanoparticle impregnated cotton gauze patches enhance wound healing in diabetic rats by suppressing oxidative stress and inflammation via modulating the Nrf2/HO-1 and TLR4/NF-κB pathways. Life sciences, 286, 120019. <https://doi.org/10.1016/j.lfs.2021.120019>

Vezza, T., Rodríguez-Nogales, A., Algieri, F., Utrilla, M. P., Rodriguez-Cabezas, M. E., & Galvez, J. (2016). Flavonoids in inflammatory bowel disease: a review. Nutrients, 8(4), 211. <https://doi.org/10.3390/nu8040211>

Wang, J., Fang, J., Wei, L., Zhang, Y., Deng, H., Guo, Y., & Meng, Y. (2019). Decrease of microbial community diversity, biogenic amines formation, and lipid oxidation by phloretin in Atlantic salmon fillets. *Lwt*, *101*, 419-426. <https://doi.org/10.1016/j.lwt.2018.11.039>

Wang, L., Wang, N., Zhao, Q., Zhang, B., & Ding, Y. (2019). Pectolinarin inhibits proliferation, induces apoptosis, and suppresses inflammation in rheumatoid arthritis fibroblast‐like synoviocytes by inactivating the phosphatidylinositol 3 kinase/protein kinase B pathway. Journal of cellular biochemistry, 120(9), 15202-15210. <https://doi.org/10.1002/jcb.28784>

Wang, M., Cai, X., Wang, Y., Li, S., Wang, N., Sun, R., & Liu, S. (2021). Astragalin alleviates neuropathic pain by suppressing P2X4-mediated signaling in the dorsal root ganglia of rats. Frontiers in Neuroscience, 14, 570831. <https://doi.org/10.3389/fnins.2020.570831>

Wang, S. H., Liang, C. H., Liang, F. P., Ding, H. Y., Lin, S. P., Huang, G. J., & Juang, S. H. (2016). The inhibitory mechanisms study of 5, 6, 4′-trihydroxy-7, 3′-dimethoxyflavone against the LPS-induced macrophage inflammatory responses through the antioxidant ability. Molecules, 21(2), 136. <https://doi.org/10.3390/molecules21020136>

Wang, W., Yue, R. F., Jin, Z., He, L. M., Shen, R., Du, D., & Tang, Y. Z. (2020). Efficiency comparison of apigenin-7-O-glucoside and trolox in antioxidative stress and anti-inflammatory properties. Journal of Pharmacy and Pharmacology, 72(11), 1645-1656. <https://doi.org/10.1111/jphp.13347>

Wang, X. S., Guan, S. Y., Liu, A., Yue, J., Hu, L. N., Zhang, K., & Liu, S. B. (2019). Anxiolytic effects of Formononetin in an inflammatory pain mouse model. *Molecular brain*, *12*, 1-12. https://link.springer.com/article/10.1186/s13041-019-0453-4

Wang, Y., Li, C., Xing, J., Zhu, Y., Sun, M., Yin, S., & Liu, S. (2023). Neohesperidin alleviates the neuropathic pain behavior of rats by downregulating the P2X4 receptor. Neurochemical Research, 48(3), 781-790. <https://doi.org/10.21203/rs.3.rs-2015859/v1>

Wójciak, M., Feldo, M., Borowski, G., Kubrak, T., Płachno, B. J., & Sowa, I. (2022). Antioxidant potential of diosmin and diosmetin against oxidative stress in endothelial cells. Molecules, 27(23), 8232. <https://doi.org/10.3390/molecules27238232>

Wu, X., Li, X., Wang, W., Shan, Y., Wang, C., Zhu, M., & Li, X. (2020). Integrated metabolomics and transcriptomics study of traditional herb Astragalus membranaceus Bge. var. mongolicus (Bge.) Hsiao reveals global metabolic profile and novel phytochemical ingredients. BMC genomics, 21(10), 1-16. <https://doi.org/10.1186/s12864-020-07005-y>

Wu, Y. P., Liang, X., Liu, X. Y., Zhong, K., Gao, B., Huang, Y. N., & Gao, H. (2015). Cedrus deodara pine needle as a potential source of natural antioxidants: Bioactive constituents and antioxidant activities. *Journal of functional foods*, *14*, 605-612. <https://doi.org/10.1016/j.jff.2015.02.023>

Xiao, X., Wang, X., Gui, X., Chen, L., & Huang, B. (2016). Natural flavonoids as promising analgesic candidates: a systematic review. Chemistry & biodiversity, 13(11), 1427-1440. <https://doi.org/10.1002/cbdv.201600060> Xing, B., Feng, N., Zhang, J., Li, Y., Hou, X., Wu, H., & Han, G. (2022). Pinocembrin relieves hip fracture-induced pain by repressing spinal substance P signaling in aged rats. *Journal of neurophysiology*, *127*(2), 397-404. <https://doi.org/10.1152/jn.00517.2021>

Xu, G., Fu, S., Zhan, X., Wang, Z., Zhang, P., Shi, W., & Xiao, X. (2021). Echinatin effectively protects against NLRP3 inflammasome–driven diseases by targeting HSP90. JCI insight, 6(2). <https://doi.org/10.1172/jci.insight.134601>

Xu, T., Wang, C., Jiang, S., Yang, T., & Wu, X. (2022). Glycosylation of luteolin in hydrophilic organic solvents and structure–antioxidant relationships of luteolin glycosides. *RSC advances*, *12*(28), 18232-18237.https://doi.org/10.1039/d2ra03300c

Xu, W., Lu, H., Yuan, Y., Deng, Z., Zheng, L., & Li, H. (2022). The antioxidant and anti-inflammatory effects of flavonoids from propolis via Nrf2 and NF-κB pathways. Foods, 11(16), 2439. <https://doi.org/10.3390/foods11162439>

Yamaguchi, M., Kinouchi, R., Morizumi, S., Shimazu, Y., & Takeda, M. (2021). Local administration of genistein as a local anesthetic agent inhibits the trigeminal nociceptive neuronal activity in rats. Brain Research Bulletin, 172, 120-128. <https://doi.org/10.1016/j.brainresbull.2021.04.015>

Yang, X., Jiang, Y., Yang, J., He, J., Sun, J., Chen, F., & Yang, B. (2015). Prenylated flavonoids, promising nutraceuticals with impressive biological activities. *Trends in Food Science & Technology*, *44*(1), 93-104. <https://doi.org/10.1016/j.tifs.2015.03.007>

Yang, Z., Nakabayashi, R., Mori, T., Takamatsu, S., Kitanaka, S., & Saito, K. (2016). Metabolome analysis of Oryza sativa (rice) using liquid chromatography-mass spectrometry for characterizing organ specificity of flavonoids with anti-inflammatory and anti-oxidant activity. Chemical and Pharmaceutical Bulletin, 64(7), 952-956. <https://doi.org/10.1248/cpb.c16-00180>

Yao, Z., Wang, L., Cai, D., Jiang, X., Sun, J., Wang, Y., & Bai, W. (2021). Warangalone induces apoptosis in HeLa cells via mitochondria‐mediated endogenous pathway. *eFood*, *2*(5), 259-270. <https://doi.org/10.53365/efood.k/145663>

Yoo, Y. M., Nam, J. H., Kim, M. Y., Choi, J., & Park, H. J. (2008). Pectolinarin and pectolinarigenin of Cirsium setidens prevent the hepatic injury in rats caused by D-galactosamine via an antioxidant mechanism. *Biological and Pharmaceutical Bulletin*, *31*(4), 760-764. <https://doi.org/10.1248/bpb.31.760>

Zada Khan, A., Muhammad, A., Anis, I., Iqbal, Z., Raza Shah, M., Khan, I., & Khan, A. (2014). Antinociceptive Potential of Viscosine Isolated form Dodonaea viscosa in Animal Models. *Journal of the Chemical Society of Pakistan*, *36*(6). <https://jcsp.org.pk/issueDetail.aspx?aid=3c908da8-1e94-46d8-b405-0ae0c8440a20>

Zafar, S., Luo, Y., Zhang, L., Li, C. H., Khan, A., Khan, M. I., & Khan, S. (2023). Daidzein attenuated paclitaxel-induced neuropathic pain via the down-regulation of TRPV1/P2Y and up-regulation of Nrf2/HO-1 signaling. *Inflammopharmacology*, 1-16. https://link.springer.com/article/10.1007/s10787-023-01225-w

Zeinali, M., Rezaee, S. A., & Hosseinzadeh, H. (2017). An overview on immunoregulatory and anti-inflammatory properties of chrysin and flavonoids substances. Biomedicine & Pharmacotherapy, 92, 998-1009. <https://doi.org/10.1016/j.biopha.2017.06.003>

Zielonka, J., Gębicki, J., & Grynkiewicz, G. (2003). Radical scavenging properties of genistein. Free Radical Biology and Medicine, 35(8), 958-965. <https://doi.org/10.1016/S0891-5849(03)00472-6>

Zhao, J., Wang, Z., Xu, D., & Sun, X. (2021). Identification of antidiabetic components from Cyclocarya paliurus. *Food Bioscience*, *44*, 101429. <https://doi.org/10.1016/j.fbio.2021.101429>

Zhang, H., & Tsao, R. (2016). Dietary polyphenols, oxidative stress and antioxidant and anti-inflammatory effects. Current Opinion in Food Science, 8, 33-42. <https://doi.org/10.1016/j.cofs.2016.02.002>

Zhang, J., Yang, S., Chen, F., Li, H., & Chen, B. (2017). Ginkgetin aglycone ameliorates LPS-induced acute kidney injury by activating SIRT1 via inhibiting the NF-κB signaling pathway. Cell & Bioscience, 7(1), 1-10. <https://doi.org/10.1186/s13578-017-0173-3>

Zhang, M. R., Jiang, K., Yang, J. L., & Shi, Y. P. (2020). Flavonoids as key bioactive components of Oxytropis falcata bunge, a traditional anti-inflammatory and analgesic Tibetan medicine. *Natural product research*, *34*(23), 3335-3352. <https://doi.org/10.1080/14786419.2019.1574786>

Zhang, L., Zhang, X., Zhang, C., Bai, X., Zhang, J., Zhao, X., & Zhao, Y. (2016). Nobiletin promotes antioxidant and anti-inflammatory responses and elicits protection against ischemic stroke in vivo. *Brain research*, *1636*, 130-141. <https://doi.org/10.1016/j.brainres.2016.02.013>

ZHANG, S. L., Peng, D. E. N. G., Xu, Y. C., Lü, S. W., & Wang, J. J. (2016). Quantification and analysis of anthocyanin and flavonoids compositions, and antioxidant activities in onions with three different colors. Journal of integrative agriculture, 15(9), 2175-2181. <https://doi.org/10.1016/S2095-3119(16)61385-0>

Zhang, T. T., Wang, M., Yang, L., Jiang, J. G., Zhao, J. W., & Zhu, W. (2015). Flavonoid glycosides from Rubus chingii Hu fruits display anti-inflammatory activity through suppressing MAPKs activation in macrophages. Journal of Functional Foods, 18, 235-243. <https://doi.org/10.1016/j.jff.2015.07.006>

Zhao, L., Chen, S., Liu, T., Wang, X., Huang, H., & Liu, W. (2019). Callistephin enhances the protective effects of isoflurane on microglial injury through downregulation of inflammation and apoptosis. Molecular Medicine Reports, 20(1), 802-812. <https://doi.org/10.3892/mmr.2019.10282>

Zhao, X., Chen, R., Shi, Y., Zhang, X., Tian, C., & Xia, D. (2020). Antioxidant and anti-inflammatory activities of six flavonoids from Smilax glabra Roxb. Molecules, 25(22), 5295. <https://doi.org/10.3390/molecules25225295>

Zhu, G. F., Guo, H. J., Huang, Y. A. N., Wu, C. T., & Zhang, X. F. (2015). Eriodictyol, a plant flavonoid, attenuates LPS‑induced acute lung injury through its antioxidative and anti‑inflammatory activity. Experimental and therapeutic medicine, 10(6), 2259-2266. <https://doi.org/10.3892/etm.2015.2827>

Zhu, M. Z., Wu, W., Jiao, L. L., Yang, P. F., & Guo, M. Q. (2015). Analysis of flavonoids in lotus (Nelumbo nucifera) leaves and their antioxidant activity using macroporous resin chromatography coupled with LC-MS/MS and antioxidant biochemical assays. Molecules, 20(6), 10553-10565. <https://doi.org/10.3390/molecules200610553>

Zoofishan, Z., Hohmann, J., & Hunyadi, A. (2018). Phenolic antioxidants of Morus nigra roots, and antitumor potential of morusin. *Phytochemistry Reviews*, *17*, 1031-1045. <https://doi.org/10.1007/s11101-018-9565-1>
